# Germline-related molecular phenotype in Metazoa: conservation and innovation highlighted by comparative transcriptomics

**DOI:** 10.1186/s13227-022-00207-3

**Published:** 2023-01-30

**Authors:** Giovanni Piccinini, Liliana Milani

**Affiliations:** Department of Biological, Geological, and Environmental Sciences, University of Bologna, Bologna, Italy

**Keywords:** Germline multipotency program, RNA-Seq, Multicellularity, PriSCs

## Abstract

**Background:**

In Metazoa, the germline represents the cell lineage devoted to the transmission of genetic heredity across generations. Its functions intuitively evoke the crucial roles that it plays in organism development and species evolution, and its establishment is tightly tied to animal multicellularity itself. The molecular toolkit expressed in germ cells has a high degree of conservation between species, and it also shares many components with the molecular phenotype of some animal totipotent cell lineages, like planarian neoblasts and sponge archaeocytes. The present study stems from these observations and represents a transcriptome-wide comparative analysis between germline-related samples of 9 animal species (7 phyla), comprehending also totipotent lineages classically considered somatic.

**Results:**

Differential expression analyses were performed for each species between germline-related and control somatic tissues. We then compared the different germline-related transcriptional profiles across the species without the need for an a priori set of genes. Through a phylostratigraphic analysis, we observed that the proportion of phylum- and Metazoa-specific genes among germline-related upregulated transcripts was lower than expected by chance for almost all species. Moreover, homologous genes related to proper DNA replication resulted the most common when comparing the considered species, while the regulation of transcription and post-transcriptional mechanisms appeared more variable, showing shared upregulated functions and domains, but very few homologous whole-length sequences.

**Conclusions:**

Our wide-scale comparative analysis mostly confirmed previous molecular characterizations of specific germline-related lineages. Additionally, we observed a consistent signal throughout the whole data set, therefore comprehending both canonically defined germline samples (germ cells), and totipotent cell lineages classically considered somatic (neoblasts and archaeocytes). The phylostratigraphic analysis supported the less probable involvement of novel molecular factors in the germline-related transcriptional phenotype and highlighted the early origin of such cell programming and its conservation throughout evolution. Moreover, the fact that the mostly shared molecular factors were involved in DNA replication and repair suggests how fidelity in genetic material inheritance is a strong and conserved driver of germline-related molecular phenotype, while transcriptional and post-transcriptional regulations appear differently tuned among the lineages.

**Supplementary Information:**

The online version contains supplementary material available at 10.1186/s13227-022-00207-3.

## Background

Many features related to obligate multicellularity already evolved in the last common ancestor of Metazoa, since they are present in all extant species. The so-called Urmetazoa were most likely bacterivorous multicellular organisms with a proto-epithelium including collar cells, able to differentiate cells in various somatic states and in anisogamic germ cells [1, 2]. Indeed, inseparably tied to the diversification of cell lineages is the existence of some cells that retain the whole potential of the organism cell states and that are devoted to the transmission of the genetic heredity across generations, i.e. the germ cells, whose lineage is called the germline. This cell lineage was a key feature for the evolution of multicellularity in Metazoa because it allowed cells within the same organism to cover diversified roles without the burden of transmission of the genome to the progeny. Some authors argued that this separation of roles, or rather the loss of totipotency in most differentiated cells, was itself the first and necessary step which allowed for the wide adaptive diversification of the somatic lineages observed in animals [3]. Indeed, once the germline is established, all somatic cells of the organism become evolutionary dead-end, and any newly arisen mutation is doomed to be extinguished with the death of the individual. Germ cells, on the other hand, are kept in a totipotent state, representing the cross-generation carriers of genetic inheritance.

With the advent of modern molecular technologies, various molecular factors and networks involved in differentiation and specification of the germline were identified for a still expanding number of species, allowing to better define and delineate a phenotype that represents one of the most ancestral metazoan features. Among the most interesting observations about the molecular profiles of germ cells is the shared expression across animals of a highly conserved gene set [4–6]. Transcription and expression of some of these genes have been observed in virtually all animals in which molecular germline characterization has been performed (see references in [6, 7]) and are usually associated with post-transcriptional regulatory activities.

For instance, the most known and phylogenetically conserved germline markers, that are *vasa*, *piwi*, and *nanos*, all show RNA-binding activities. The *vasa* gene encodes for a DEAD-box RNA helicase associated to germline specification and differentiation in virtually all animals, with functions spanning from translational activation to chromatin condensation (functions reviewed in [8]); *piwi*, that encodes for a protein of the Argonaute family, is strictly related to the Metazoa-specific piRNA-mediated RNA silencing, mostly involved in germline-specific retrotransposon silencing [9–11]; *nanos* homologues, on the other hand, encode for a diverse set of proteins with a widely conserved C-terminal zinc-finger domain (CCHC type) that mediates RNA-binding activity controlling mRNA translation fates [12, 13].

Many other molecular factors have been associated to germ cell specification/differentiation in different animals through the years, and most of them are associated to RNA regulation (for a review on the molecular machinery of germline specification see [4]), such as Boule, Pumilio, the Tudor protein family, germ-cell-less, and Bruno [14–18]. Among all these genes, however, *vasa*, *nanos*, and *piwi* are those that are mostly shared in the germline of different Metazoa, making them quasi-universal markers of germ cells for almost all differentiation stages. Most other factors are indeed transcribed and expressed in specific germ cell stages, and/or they have not been associated to germline functions in all animals (see for instance the summary tables of germline determinants in: [4–7]). Moreover, while the evolution of most germline-associated genes predated the separation of the animal lineage from other eukaryotes, *vasa*, *nanos*, and *piwi* (together with some strictly germline-related Tudor proteins) are thought to be specific metazoan innovations. Indeed, so far orthologues have not been found in other eukaryotic lineage, differently from other germline determinants that have been annotated in other holozoan (e.g. *bruno*, *pumilio*, and *boule*; [6, 19]).

Many germ cell molecular determinants were observed as expressed also in some animal multipotent cell lineages that have also somatic fates. For instance, cases of embryonic cells with mixed somatic–germ fates were reported expressing such genetic factors, whose presence indeed preceded the actual determination of strict germ cell fate (e.g. the small micromere lineage of *Strongylocentrotus purpuratus* [20]; the 4d lineage in mollusc embryos [21]; the cells of the mesodermal posterior growth zone in annelid embryos [22]). Moreover, the expression of germline determinants was observed as not limited to embryonic stem cells, but as present also in adult stem cells of different animal lineages that share extensive regenerative capabilities: stem cells involved in posterior elongation during post-caudal regeneration in annelids [23], multipotent regenerative interstitial cells of Hydrozoa [24], totipotent archaeocytes and choanocytes in sponges [6, 19, 25], neoblasts in free-living flatworms (reviewed in [26]) and acoels [27], stem cells of blood vessel epithelia in tunicates [28], and others.

Altogether, these observations suggest a broad molecular similarity between germ cells and stem cells, leading to theorizing the germline multipotency program (GMP), a genetic toolkit that operates both in germline and somatic multipotent stem cell lineages and that is fundamental for establishing and maintaining multipotency [5]. Later, Solana synthesized two centuries of germline-associated morphological and molecular studies by proposing the definition of primordial stem cells (PriSCs; [29]), that are highly conserved stem cells that include all stages that exist between the zygote and the first specified cells with exclusive germ cell fate (i.e. primordial germ cells, or PGCs). The author proposed these PriSCs, despite their mixed germline-somatic potential, to be included into the germline, that would then comprise all cells potentially capable of producing a germ cell, solving theoretical controversies regarding the continuity of the germline throughout generations raised by classical definitions of germline. Accordingly, all aforementioned examples of stem cells with both somatic and germline potential can be considered PriSCs, establishing a continuity from zygote to germ cells and collecting within the same definition, and perhaps within the same homologous lineage, totipotent cell lineages.

In the present analysis we aimed to explore the transcriptional signatures of germline-related tissue/cell lineages in different animals by taking advantage of high-throughput RNA-Seq experiments, that provide snapshots of the overall transcriptional profile of the samples, allowing for investigations without the need to determine an a priori set of germline determinants. We used online-available experimental data to retrieve all the RNA-Seq experiments that fit the established features of having enough samples size to assess transcript abundance, and of having control somatic samples produced within the same experiment. We performed species-specific differential expression (DE) analyses, and we then checked whether there were homologous genes upregulated in the germline-related samples for most of the species, to retrieve a common transcriptional signal that could have emerged despite the data set heterogeneity. We indeed observed an overall shared enrichment toward DNA proper replication, both for co-upregulated homologues and co-enriched Gene Ontology (GO) terms and InterProScan (IPR) codes. Moreover, using reference proteomic data from other animals, we looked into the upregulated germline-related transcripts to get hints on how many of them were lineage-specific innovations. We observed that in germline-related samples there was a general enrichment toward the upregulation of anciently derived genes.

## Results

### Differential expression of germline-related transcripts: upregulation is biased toward phylogenetically conserved genes

**Fig. 1:**
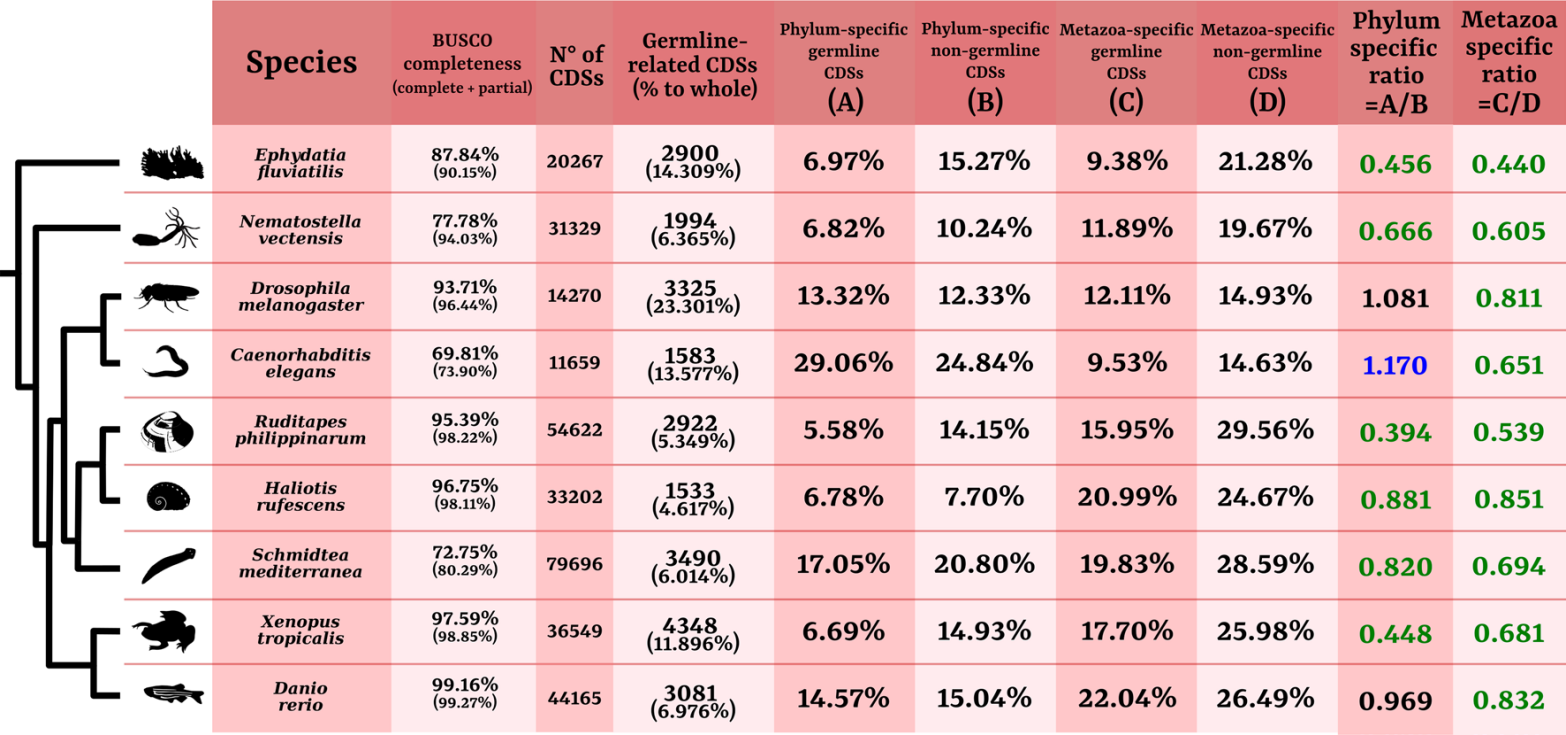
Transcriptomic statistics. Phylogenetic relationships between the species are schematized on the left (referring to [33]). BUSCO completeness is calculated on the whole transcriptome. N° of CDSs represents the number of transcripts for which an ORF could be extracted, i.e. coding sequences. Germline-related CDSs correspond to the number of ORF-containing transcripts that were upregulated in germline-related samples (the percentage is calculated on the whole set of ORF-containing transcripts). Phylum-specific germline CDSs corresponds to the percentage of upregulated germline-related CDSs for which not even one homologous sequence could be found outside the belonging phylum. Phylum-specific non-germline CDSs is the same percentage calculated on all other transcripts (non-germline ones). Metazoa-specific germline CDSs is the percentage of germline-related CDSs that had homologues in at least two animal phyla (therefore, excluding the phylum-specific ones), but no homologues shared with unicellular Holozoa. Metazoa-specific non-germline CDSs is the same percentage calculated on all other transcripts (non-germline ones). Phylum-specific ratio is calculated as column A over column B. Metazoa-specific ratio is calculated as column C over column D. Phylum- and Metazoa-specific ratio significantly lower than 1 are depicted in green; those higher are depicted in blue (statistical significance assessed with odds ratio tests)

RNA-Seq reads were retrieved by NCBI selecting experiments that included germline-related samples, somatic control samples, and at least two biological replicates per condition. The final dataset comprehended 9 species covering 7 phyla (Table 1; Fig. 1): *Ephydatia fluviatilis* (Porifera), *Nematostella vectensis* (Cnidaria), *Caenorhabditis elegans* (Nematoda), *Danio rerio* (Chordata), *Xenopus tropicalis* (Chordata), *Drosophila melanogaster* (Arthropoda), *Ruditapes philippinarum* (Mollusca), *Haliotis rufescens* (Mollusca), and *Schmidtea mediterranea* (Platyhelminthes). Due to the lack of high-quality genomes for some of the species considered, all transcriptomes were assembled de novo to standardize the methods across the data set (see “Methods” section for details; species-specific transcriptomic statistics are available in Additional file 1). The BUSCO quality check of the filtered assemblies revealed high levels of completeness for most of the filtered transcriptomes, with a proportion of complete + partial core genes always higher than 94% (Fig. 1; see “Methods” for assembly details).

**Table 1:** Sample composition for the 9 species included in the data set

Species	Phylum	BioProject (NCBI database)	Germline-related samples (n° replicates)	Control somatic samples (n° replicates)
*Caenorhabditis elegans*	Nematoda	PRJNA392422	Embryonic primordial germ cells (3)	Embryonic somatic cells (3)
*Danio rerio*	Chordata	PRJEB30097	Gonads (2)	Livers (2)
*Drosophila melanogaster*	Arthropoda	PRJNA388952	Gonads (4)	Genitalia (4)
*Ephydatia fluviatilis*	Porifera	PRJNA244851	Archaeocytes (2)	Mixed differentiated cells (2)
*Haliotis rufescens*	Mollusca	PRJNA488641	Gonads (2)	Mantles (2)
*Nematostella vectensis*	Cnidaria	PRJNA667495	Gonads (3)	Muscles and mesenterial filaments (6)
*Ruditapes philippinarum*	Mollusca	PRJNA672267	Gonads (8)	Mantles (8)
*Schmidtea mediterranea*	Platyhelminthes	PRJNA503908	Neoblasts (3)	Mixed differentiated cells (3)
*Xenopus tropicalis*	Chordata	PRJNA381064	Gonads (2)	Hearts and livers (4)

The number of replicates for each sample represents biological replicates

*S. mediterranea*, *C. elegans*, and *E. fluviatilis* had lower completeness statistics but nevertheless not so low to invalidate subsequent analyses (complete + partial: 80.29%, 73.90%, and 90.15%, respectively). Lower values for these 3 species could be due to lineage-specific diversification: indeed, BUSCO results for the online-available proteomes obtained from genome annotations of *S. mediterranea, C. elegans*, and the *E. fluviatilis* congeneric species *E. muelleri* are similar, if not lower, to our results (complete + partial: 81.13%, 80.61%, and 77.36%, respectively; for the proteomes used, refer to Additional file 2: Table S1). Our BUSCO results are therefore most likely due to a combination of this and the sample types of the RNA-Seq experiments, since these three specific samples were cell populations, and not tissues as for the other 6 species (see Table 1). Indeed, it is more likely to miss transcription of some core gene in cell populations rather than in pools of different tissues that comprise diverse cell lineages and stages. However, the levels of completeness were still relatively high, and the lower levels might have brought the subsequent analyses toward false negatives rather than false positives, therefore not invalidating the obtained result but at most limiting the detection power.

We were interested in protein-coding genes only, therefore we considered for the subsequent analyses only transcripts that included a predicted open reading frame (ORF), i.e. coding sequences (CDSs). From now on, when we refer to “transcripts” we mean ORF-including transcripts, i.e. those supposedly belonging to protein-coding genes, and when we refer to “translated transcriptome” we intend the translated ORFs.

On average, ~ 10% of each species transcriptome was upregulated in germline-related samples with respect to somatic controls (twice as transcribed, *p*-value < 10^−3^; see “Methods” section; Fig. 1). An interesting observation was represented by the percentages of phylum-specific germline-related CDSs (i.e. CDSs that did not share homology with any other sequence outside the belonging phylum; phylostratigraphic analysis performed with 111 additional holozoan proteomes, see “Methods” section for details). Indeed, such statistics differed widely between species, passing from 5.6% for the bivalve mollusc *R. philippinarum*, to roughly 29% in the nematode *C. elegans* (overall mean of 11.9%, with 64.8% coefficient of variability; Fig. 1).

By calculating also the phylum-specific percentage of non-upregulated genes (i.e. the rest of the translated transcriptome), and comparing it with the germline-related percentage, we assessed whether there was any over-representation of intra-phyletic or inter-phyletic homology in the germline sequence subsets (see “Methods” for ratio calculation: phylum-specific percentage of germline-upregulated genes/phylum-specific percentage of non-upregulated genes; Fig. 1).

- If the ratio was lower than 1, then it would mean that it was more likely for a germline-related upregulated CDS to share homology with at least another sequence of another phylum, i.e. germline-upregulated transcripts were depauperated of lineage-specific CDSs.
- On the contrary, a ratio higher than 1 meant that the germline-related upregulated subset had a higher proportion of phylum-specific CDSs with respect to the rest of the translated transcriptome, i.e. germline-upregulated transcripts were enriched for lineage-specific CDSs.

Two thirds of the data set had a ratio lower than 1 (significant on a odds ratio test), indicating a bias toward germline-related upregulation of shared inter-phyletic genes (ratios are summarized in Fig. 1). However, 2 species, namely *D. melanogaster* and *D. rerio*, did not display any evident bias in the germline-related samples, neither toward phylogenetically conserved genes, nor toward clade-specific ones. *C. elegans*, on the other hand, displayed the opposite trend, with a higher percentage of phylum-specific germline-related transcripts with respect to the rest of the transcriptome (1.17 phylum-specific ratio).

An analogous signal that we observed in all 9 species was represented by the Metazoa-specific percentage ratios calculated for all those sequences whose homologues were shared by at least two phyla. If these sequences did not have any homologue outside Metazoa (8 species covering the 4 major unicellular Holozoa taxonomic groups were included in the phylostratigraphic analysis), they were considered as Metazoa-specific. We could observe that the percentage of these Metazoa-specific CDSs was lower in germline-upregulated transcripts with respect to the rest of the translated transcriptome for all species, meaning that it was more likely for a holozoan-shared CDSs to be differentially transcribed in germline-related samples.

### Shared germline-related homologous sequences: high representativeness of DNA replication-related genes

Given the heterogeneity of the data set, instead of concentrating on the species-specific results, we focused on the strongest signals that emerged in the different DE analyses and compared them across the samples (all species-specific results are nevertheless accessible in Additional file 1).

**Fig. 2:**
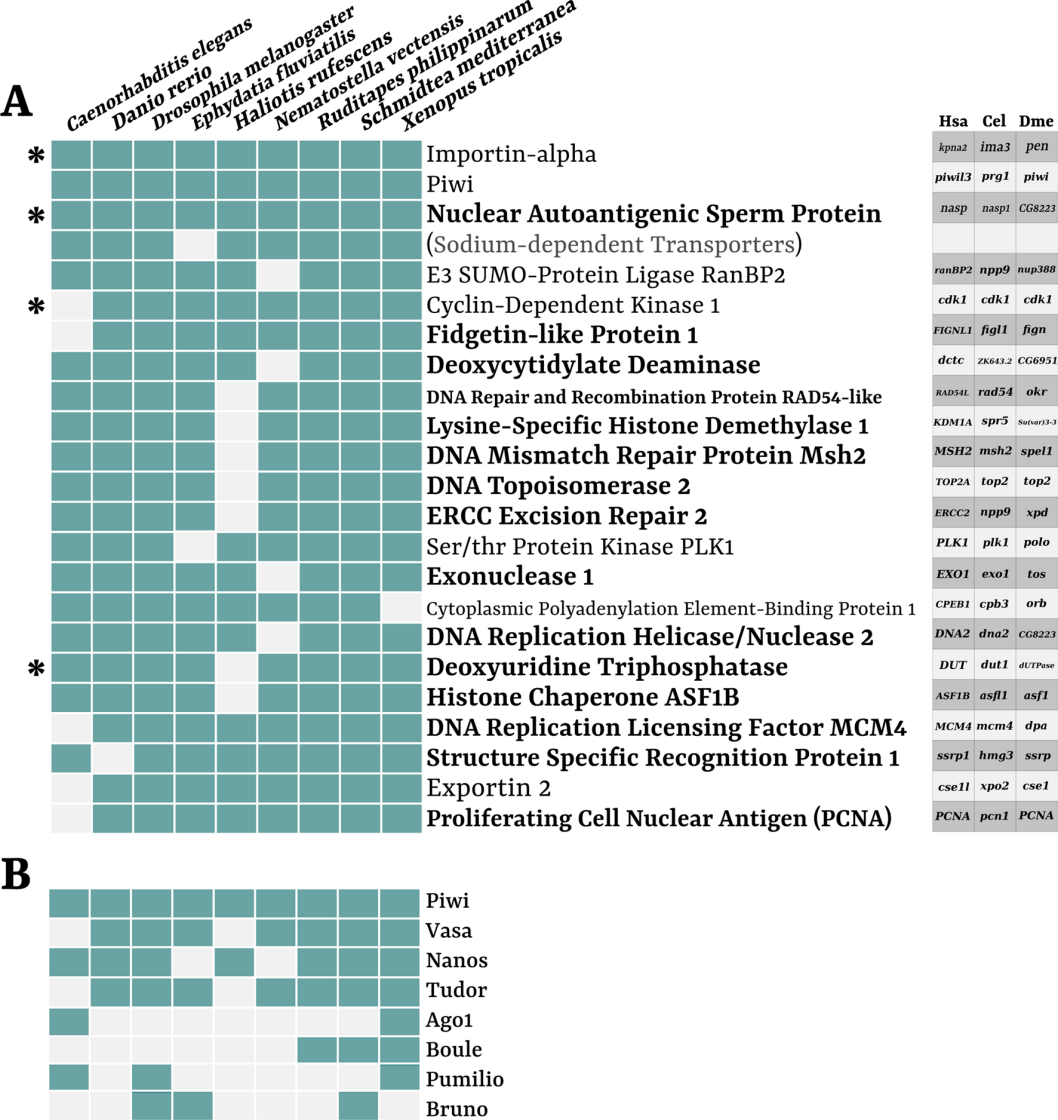
Upregulated germline-related OrthoGroups (OGs) shared by 8 species or more. **A** The table represents presence (light blue) or absence (light grey) in different species (columns) of germline-related differentially transcribed genes belonging to different OGs (rows). On the right of each row is reported the annotation of proteins encoded by genes included in the respective OGs: bold names represent proteins associated to DNA-related activities; Sodium-dependent transporters is included between parentheses because it represents a protein family, since the corresponding OG was a large cluster of homology, and not a defined orthology group. On the right, a table summarizes the gene nomenclature in three model species (Hsa: *Homo sapiens*; Cel: *C. elegans*; Dme: *D. melanogaster*). Asterisks on the left are associated to those genes whose transcription was upregulated in germline-related samples in 8 or more species also with other more stringent logFC cut-offs and DESeq2/edgeR intersection (see “Methods”). **B** The lower table represents absence/presence in the species (columns refers to upper table A) of transcripts of genes commonly associated to GMP (row names are gene products). Excluding Piwi (present also in the upper table A), only Nanos, Vasa, and Tudor were upregulated in a conspicuous number of species in our data set (see Additional file 2: Fig. S1 for GMP domain enrichment)

To observe whether there were any homologous CDSs upregulated in different species of our data set, we constructed clusters of homology for the whole translated transcriptomes of our 9 species. CDSs of different species were considered co-upregulated in germline-related samples when they were significantly upregulated and belonged to the same OrthoGroup (OG). We identified 3794 OGs that included germline-related CDSs upregulated in germline-related samples of at least two species of our data set. Out of these, 3 OGs were upregulated in all species (Fig. 2). These OGs included homologues encoding for Importin-alpha (one of the two subunits of importin, involved in protein import inside the nucleus, but also in centrosome duplication and mitotic spindle dynamics), the Nuclear Autoantigenic Sperm Protein (NASP, a histone-binding protein involved in DNA replication-dependent nucleosome assembly), and Piwi (the already cited nearly ubiquitous germline marker with a central role in the piRNA pathway of retrotransposon silencing).

We also specifically annotated the content for the 20 OGs with germline-related upregulated sequences shared by all but one species (i.e. 8 species, with a variable missing one; Fig. 2A). These OGs included 14 genes whose products have activities directly related to DNA (especially proper DNA replication; subsequent protein names refer to vertebrate nomenclature, see Fig. 2A for other model species): Fidgetin-like Protein 1 (FIGNL1), Deoxycytidylate Deaminase (DCTD), DNA Repair and Recombination Protein Rad54-like, Lysine-specific Histone Demethylase 1 (KDM1A/B), DNA Mismatch Repair Protein MSH2, DNA Topoisomerase 2 (TOP2), ERCC Excision Repair 2 (ERCC2), Exonuclease 1 (EXO1), DNA Replication Helicase/Nuclease 2 (DNA2), Deoxyuridine Triphosphatase (DUT), Histone Chaperone ASF1B, DNA Replication Licensing Factor MCM4, Structure Specific Recognition Protein 1 (SSRP1), and Proliferating Cell Nuclear Antigen (PCNA). Moreover, 5 of these gene products were involved in DNA repair mechanisms (see “Discussion” and Fig. 2A). Other OGs co-upregulated in 8 species included 2 transcripts encoding for proteins related to the nuclear pore (E3 SUMO-protein Ligase RanBP2, and Exportin 2), 2 transcripts involved in the regulation of the mitotic phase of the cell cycle (Cyclin-dependent Kinase 1, and Ser/thr Protein Kinase PLK1), and the mRNA regulator CEPB1 (Fig. 2A). By iterating the DE analyses with more stringent cut-offs (see “Methods” section), we observed that most of these genes were still co-upregulated in most species, therefore suggesting the observation robustness (see Additional file 1).

The remaining 8-species OG represented a noisy large homology cluster, where only few sequences were actually upregulated in germline-related samples (approximately 1/10 of the CDSs included in that OG). OrthoFinder homology inference is, indeed, prone to collapsing within the same OG different genes belonging to the same gene family, or that simply share some specific domains. This happens especially when domains are common in the proteome, in multiple copies within the same proteins, and follow complex pattern of acquisition/loss in the proteome, reflecting a network-like homology of conserved protein regions. For this reason, a clear whole-length homology could not be retrieved for the germline-related subset included in such cluster.

We also looked specifically for GMP-associated genes that previous studies reported as expressed in germline/multipotent cell lineages (see “Background”). These genes were namely *piwi*, *ago1, vasa*, *boule*, *nanos*, *pumilio*, *bruno*, and *tudor* (referring to *D. melanogaster* nomenclature), and we identified their belonging OGs based on the *D. melanogaster* sequences. With the exclusion of *piwi* (already cited since it was included in the OGs shared by all species), the only GMP genes that were upregulated in a conspicuous number of species were *vasa, nanos,* and *tudor* (shared by 7 species out of 9; Fig. 2B). The other ones were shared only by 2 to 3 species. The situation slightly improved when considering the representative domains of the proteins instead of the full-length homologous sequences (Additional file 2: Fig. S1). For instance, most domains and motifs associated to *vasa* were enriched in the germline-related samples of all species; and the RNA recognition motif, present in both *bruno* and *boule*, was enriched in the germline-related samples of two-thirds of the data set (Additional file 2: Fig. S1), while the homologous whole-length CDSs of their belonging genes were much less represented (Fig. 2B).

We then looked at all other OGs that contained sequences upregulated in at least 2 species. The 2-species combinations (i.e. OGs upregulated in 2 species only) were the predominant ones, significantly deviating from the expected random distribution: they corresponded to 1803 of the 3794 germline-related OGs (Additional file 2: Fig. S2). Of these 2-species combinations, those that displayed a higher degree of positive deviation from random expectations were the couples *Danio–Xenopus* (Chordata), *Drosophila–Xenopus*, *Haliotis–Ruditapes* (Mollusca), and *Ephydatia–Nematostella* (two early branching non-Bilateria species), therefore reflecting a weak phylogenetic signal. Interestingly, out of all the combinations of 3 or more species, those that displayed a positive deviation from expected values were 6-, 7-, 8-, and 9-species combinations, while the 3-, 4-, and 5-species combinations had negative deviations, hence they were represented in lower numbers with respect to random distributions.

### DNA-related functions and domains are enriched in germline-related samples

We annotated domains and GO terms for all CDSs of our dataset and performed for each species an enrichment analysis to highlight over-represented GO terms within germline-related upregulated sets of transcripts. We then compared the results obtained across the different species. Given the diversity of our data set and the non-specific nature of GO terms, we decided to look in a comparative manner only the strongest signals emerged: only those GO terms that were annotated in germline-related transcripts at least twice as much as randomly expected, and we focused on those that were shared by at least 6 species. Moreover, to test the robustness of the analysis, we iterated the enrichment with different algorithms and considering progressively stringent cut-offs for the upstream DE analyses (see “Methods” section).

**Fig. 3:**
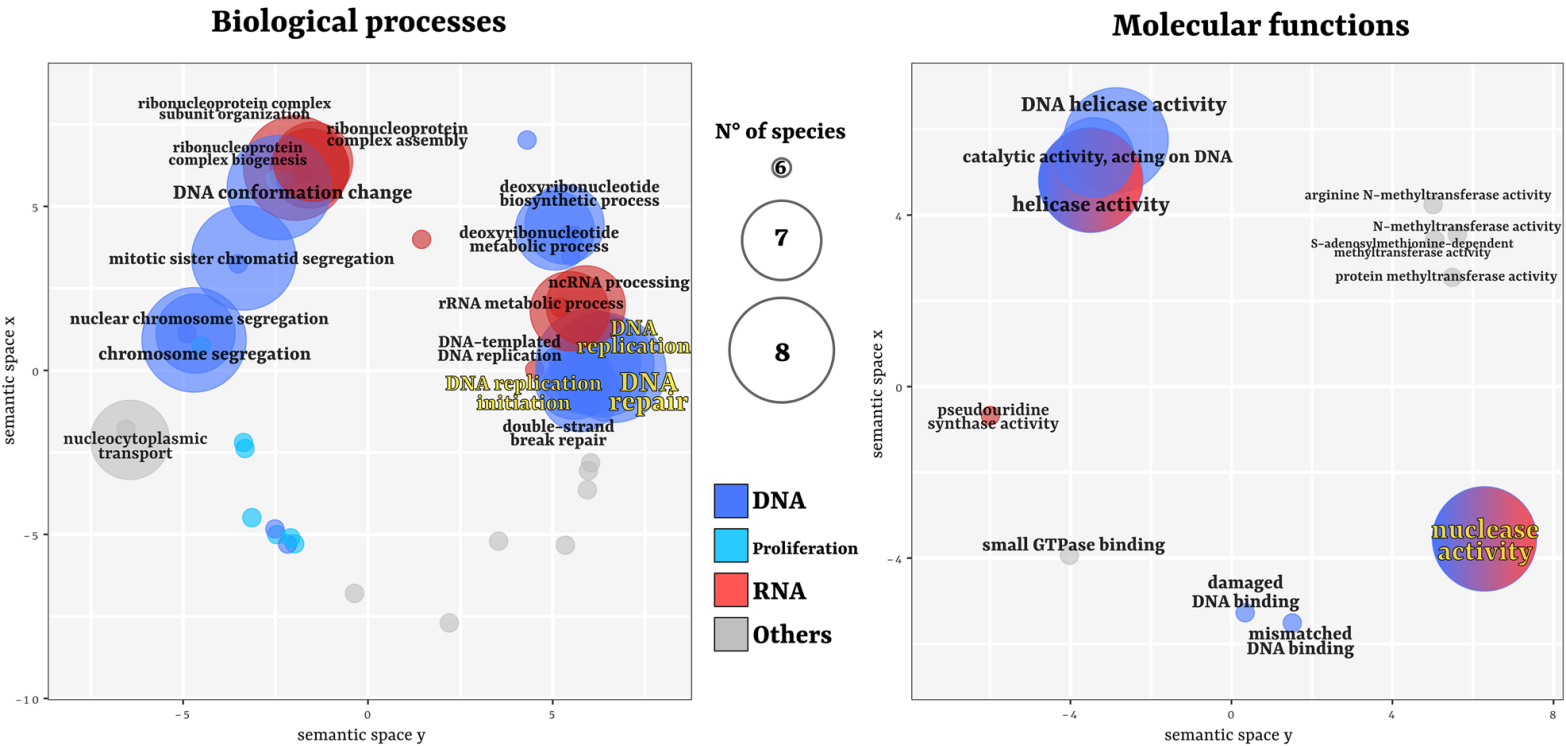
Co-enriched germline-related GO terms shared by 6 or more species. The semantic plot on the left corresponds to GO terms that define biological processes, and names are explicit for those present in 7 species or more. The semantic plot on the right corresponds to GO terms that define molecular functions. For the full set of GO terms, see Additional file 2: Fig. S3, S4 and Additional file 1. The size of the circles is scaled with the number of species that share that specific term in their germline-related samples (size legend in the middle). Terms highlighted in yellow are those that are co-enriched in 6 species or more consistently throughout all different logFC cut-offs, all topGO algorithms, and considering both the union and intersection of DESeq2 and edgeR DE results

We could observe many GO terms significantly associated to germline-related samples shared by at least two-thirds of the data set (results shown in Fig. 3, split in Biological Processes and Molecular Functions; for complete list and presence/absence in the species refer to Additional file 2: Figs. S3, S4; for extensive species-specific results refer to Additional file 1). Altogether, approximately half of these co-enriched GO terms were related to proliferative processes, with a high representativeness of direct DNA-related functions (34 out of 69 terms for the biological process category, 10 out of 18 for the molecular function one). No GO terms were enriched in all 9 species, and those terms co-enriched in 8 species were almost all related to DNA replication (except for the RNA-related “ribonucleoprotein complex biogenesis”, and “nuclease activity” and “helicase activity” that can be associated to both DNA and RNA). Among those co-enriched in 7 species, despite the usual DNA replication terms, also more ncRNA-related terms were present, together with DNA repair-associated ones. Moreover, the only 3 GO terms that were enriched in more than two-thirds of the data set consistently with all different algorithms and DE analyses cut-offs were “DNA replication”, “DNA replication initiation”, “DNA repair”, and “Nuclease activity”, thus representing the most robust signals (Fig. 3).

**Fig. 4:**
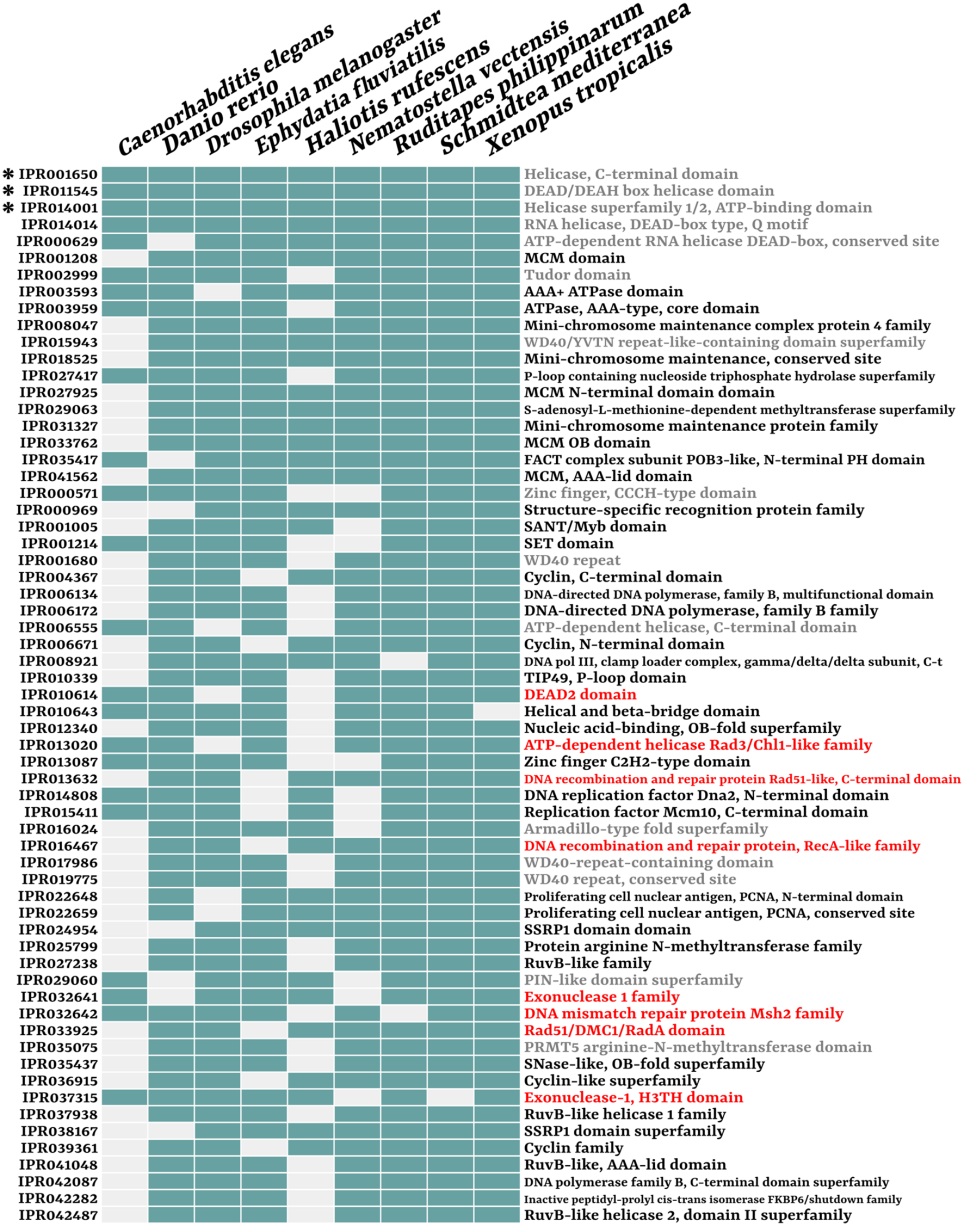
Co-enriched germline-related IPR codes in 7 or more species. For IPR codes shared by 6 species, see Additional file 2: Fig. S5. The table represents presence (light blue) or absence (light grey) in different species (columns) of germline-related enriched IPR codes (rows). On the right of each row is reported the description of the respective IPR codes: bold names represent codes associated to direct DNA-related or proliferative activities; names in red refer to codes associated to DNA repair mechanisms. Many of the codes in the present table refer to genes that were also detected among the co-upregulated OGs (see Fig. 2A). However, despite being partially overlapping, the analysis was independent and revealed additional signals to the whole-length sequence homology one (see for instance all codes enriched in 9 species). Asterisks refer to IPR codes that are enriched in 8 or more species considering all iterations of the species-specific DE analyses with different cut-offs

These results were coherent with the partially overlapping, but nevertheless independent, analysis on IPR codes, that are annotation codes corresponding to both domains, motifs, and protein families (Fig. 4 and Additional file 2: Fig. S5; for species-specific results see Additional file 1). Indeed, out of 173 IPR codes over-represented (odds ratio test; see “Methods” section) in the germline-related samples of more than two-thirds of the data set, 92 were associated to DNA-related activities, of which 66 directly associated to DNA replication. Moreover, there was also a relatively high amount of over-represented IPR codes linked to DNA repair (19 codes, nearly 11% of the total) and, interestingly, the mechanisms to which they were related to were both replication-dependent (e.g. domains and protein families of Rad50, Rad51, RecA, and others, involved in double-strand break repair, base excision repair, and recombination-related repair) and replication-free ones (e.g. domains associated to TFIIH subunits, ERCC4, and others, involved in nucleotide excision repair).

Besides DNA-associated functions, among other GO terms and IPR codes over-represented in the germline-related samples of most species there was a high proportion of RNA regulation signals (11 biological process GO terms, 3 molecular function GO terms, and 47 IPR codes). The mechanisms involved ranged from ribonucleoprotein complex assembly to mRNA binding and translational regulation. Contrarily to DNA replication, that was also highly represented in the co-upregulated homologues (see previous “Results” section; Fig. 2A), RNA-related biological processes were mostly represented by more general signals like GO terms and IPR codes.

## Discussion

### Considerations about the reliability of the samples and the analytical approach

This data set was extremely heterogeneous in sample composition (see Table 1), an unavoidable flaw of using online-available data that were not originally intended for such comparative analyses. However, not only having such a wide variety of animals and samples is otherwise very difficult to plan in a dedicated experiment, but we are also convinced of the reliability of our analysis, despite its inherent limits. We believe that both the fact that the somatic controls belonged to different non-homologous tissues, and the fact that what we call germline-related samples were whole gonads for some species and cell populations for others, did not compromise the principles of the analysis, but rather its power. The heterogeneous nature of our data set might have prevented a strong signal to emerge, but if something could be observed, it meant that a shared signal was indeed present in the common denominator of all samples, that is the germline. In other words, we are convinced that our study was not subjected to the risk of observing false positives, but, rather, to the risk of having a great number of false negatives. Indeed, by considering only transcripts upregulated twice in germline-related samples (with *p*-values lower than 10^−3^) and GO terms enriched in twice as much transcripts as expected (together with progressively stringent cut-off iterations), we observed only the strongest signals. In this way we should have overcome spurious results related to non-homology across control somatic tissues. Moreover, we limited our discussions on molecular signals that were shared by most of the species (8 or more species for co-upregulated genes; 6 or more species for GO terms and IPR codes).

Indeed, we could assess the shared presence of GMP genes in the subset of germline-related upregulated transcripts (Fig. 2). The 4 more characterized genes, that are *piwi*, *vasa*, *nanos*, and *tudor*, could be found in almost all samples (in all 9 species the former, in 7 species the others), comprising the non-classical germline lineages, i.e. archaeocytes and neoblasts, that are associated to the same genetic programming of germ cells, as previously said [5, 29]. In the species in which these key genes were not upregulated, the transcripts were present in the germline-related samples, but nevertheless with transcription levels comparable to somatic controls (except for *C. elegans tudor* homologue, that was missing in the OG). However, for *H. rufescens*, *vasa* and *tudor* homologues were more transcribed in the germline-related samples, but below significance cut-offs. Since *H. rufescens* was one of the most frequently lacking species in the co-upregulated (Fig. 2A) and co-enriched GO/IPR analyses (Fig. 4; Additional file 2: Figs. S3, S4, S5), we checked also for the transcription levels of other co-upregulated genes. Indeed, in most of the 7 cases were *H. rufescens* was missing a co-upregulated homologue (Fig. 2A), the gene was more transcribed in the germline-related samples, but below significance. The reasons behind this might be found in the RNA-Seq experiment itself, whose low sample size (both in number of replicates and in library size) potentially weakened the resolution of the DE analysis (see Additional file 1 for evaluations of the species-specific DE analysis). This, however, was not an unbearable issue for the present work since, as it is conceived, it inflated the number of false negatives, but not that of false positives (therefore not invalidating the obtained results).

Therefore, the presence of the GMP signature genes adds solidity to our approach, that, despite sample heterogeneity, was able to retrieve features common across the species considered, and that were in line with previous work. This allowed us to discuss other transcriptional results that did not comprehend a priori characterization of known genes.

### Germline-related genes are more frequently conserved across Metazoa

An interesting signal that we retrieved by assessing the percentages of clade-specific genes transcribed in our data set species was the fact that, for many of them, genes upregulated in germline-related samples were more conserved across Metazoa than expected by chance (Fig. 1). On average, ~ 88% of each species subset of germline-related transcripts had homologues in at least another metazoan phylum. However, this percentage has no meaning if not compared with the phylum-specificity percentage of all other non-germline transcripts. Indeed, when comparing the two percentages, it came out clear that there was indeed a bias toward cross-phyla genes in germline-related samples (Fig. 1). The ratio between those two percentages, that we called phylum-specific ratio, was below 1 in most species, suggesting that, for a newly arisen lineage-specific gene, it is less likely to be involved in genetic pathways associated to the germline. Coherently, when considering only the species of our data set, the number of co-upregulated OGs comprising 2, 6, 7, 8, and 9 species deviated positively from random distributions, while OGs comprising 3, 4, and 5 species deviated negatively (Additional file 2: Fig. S2). Most of the shared transcriptional combinations were those shared by 2 species only, that partially reflected phylogenetic relationships (among the highest deviations were the couples *Danio–Xenopus*, *Ruditapes–Haliotis*, and *Ephydatia–Clytia*). However, excluding combinations of 2 species only, it is interesting to notice that the combinations comprising more species (6 to 9) were more frequent with respect to random expectations than those comprising less species (3 to 5).

The only opposite signal was represented by the higher than 1 phylum-specific ratio of *C. elegans*. One explanation could lie in the nature of its specific biological samples, that are cells from early stages of embryo development. In a previous work, by comparing developmental stages of species belonging to 10 phyla, it has been suggested how the earliest stages of development have a greater proportion of co-expressed genes between species belonging to different phyla: the so-called inverse hourglass model of development [30]. Coherently, in embryos of different spiralian phyla, early stages of development share a higher transcription of phylogenetically older genes with respect to mid stages [31]. These observations might explain the case of *C. elegans* in the present work. Our transcriptome was assembled with reads belonging exclusively to samples in early stages of embryo development. This could have caused an overall bias toward genes shared by multiple phyla, that might have weighted more on the non-germline genes (considering that they are more numerous and involved in more aspects of embryo development). This bias might have covered in *C. elegans* the signal observed in other data set species, resulting in a significantly positive phylum-specific ratio. This hypothesis to interpret the outlier *C. elegans* should be, however, properly tested by comparing germline–soma phylostratigraphic data in other species and other developmental stages.

When considering genes shared by multiple phyla, the Metazoa-specific ratio is lower than 1 for all 9 data set species (Fig. 1). This indicates in germline-related samples an enrichment for the upregulation of genes that share homology outside Metazoa, i.e. with at least one of the unicellular Holozoa species included in our data set. The germline, considered in its wide meaning as any cell that can produce a germ cell (see “Background” and [29]), is one of the most shared cell lineages that can be found in animals. Regardless of whether germline establishment was the adaptive driver of multicellularity [3], or if it was one of the first evolving lineages, it is undoubted that its presence represents a major phenotypic trait shared by all animals, given that their last common ancestor was most likely an oogamic multicellular organism [32]. Our results support both a germline early origin and its conservation throughout animal evolution, since with the phylostratigraphic analysis we observed that newly evolved genes were less likely to get included in such lineage, both considering newly evolved metazoan genes and newly evolved phylum-specific ones.

This signal was particularly strong also for *E. fluviatilis*, the representative of Porifera in our data set. This species belongs to an early-branching metazoan taxon (whether it represents the earliest-branching clade is still a matter of debate [32, 33]) that has been usually associated with ancestral metazoan features. In the present analysis, the germline-related samples considered in this species were archaeocytes, cells proposed as being very similar to the ancestral type of animal stem cells [19]. Archaeocytes are totipotent cells involved both in sponge tissue regeneration, and in sexual and asexual reproduction. Indeed, they can produce both gametes (specifically oocytes) and asexual gemmules, i.e. thousands of packed archaeocytes that are released in the environment where they hatch and give rise to new juvenile individuals [34].

We could observe a very low phylum-specific ratio, suggesting that archaeocytes have indeed a transcriptomic profile that involves more conserved genes that are datable to older evolutionary times. Coherent results were retrieved in a recent work [35]: in the species *Amphimedon queenslandica* (a demosponge like *E. fluviatilis*), they analysed transcriptomes of archaeocytes, choanocytes, and pinacocytes (other two lineages that were proposed as cell states similar to early animal cell lineages) and saw that the percentage of upregulated sponge-specific transcripts was much lower in archaeocytes. Their number were different from ours in absolute values (different species, methods, and tools), but the ratio of that percentage over the sponge-specific percentage of the whole genome as calculated in their work was interestingly similar to our results (0.4). Moreover, also in their analysis the percentage of upregulated pre-metazoan genes was higher in archaeocytes, as in the present study. Lastly, they could also observe strong statistical significance when comparing the archaeocyte transcriptomic profile to that of two holozoan: the choanoflagellate *Salpingoeca rosetta* in the colonial stage (but not in sessile or swimming stage), and the ichthyosporean *Creolimax fragrantissima* in the multinucleate stage (but not in the amoeboid stage). They interpreted all these data as the fact that the ancestral metazoan cell type resembled modern transdifferentiating stem cells [35].

A similar inter-phyletic phylostratigraphic signal was shared by most of the germline-related samples of our data set, including *S. mediterranea* neoblasts. This observation suggests us the possibility to include in a general discussion totipotent lineages as a whole, further providing hints on the similarities between stem and germ cell lineages. Remarkably, in the totality of our data set pre-metazoan genes are more likely involved in germline-related pathways than expected by chance, as highlighted by the Metazoa-specific ratio below 1. Despite the mentioned heterogeneity of the used data set, both in samples (i.e. whole gonads or cell lineages) and stages (i.e. early stages of differentiation or late ones), the same signal was obtained for different species despite their supposedly ancestral or derived state (from cnidarian to molluscs and chordates). For this study we utilized all the suited, online-available experiments, but we would be eager to extend the pipeline to other species as soon as new data will be available and see whether the trend still stand.

### Co-upregulated OGs are coherent with germline-related totipotent processes and biased toward DNA replication and cell cycle progression

When looking at homologous genes that are upregulated in most samples (8–9 species), it is clear how most of them can be collected in DNA-related activities, and especially in DNA replication and DNA repair (Fig. 2A), that represent basic cellular processes associated with proliferation and mitotic/meiotic activity. If considering also nuclear import/export activities and cell cycle progression the bias grows stronger, arriving to comprise 20 out of 23 OGs that were co-upregulated in 8 species or more. Such signal was also very strong when considering shared significantly enriched GO terms (Fig. 3) and IPR codes (Fig. 4).

The over-representation of upregulated DNA replication-associated factors with respect to transcriptional activators and promoters suggests a higher level of conservation across the species of such key cellular process. According to our results, the regulation of transcription appears to be more lineage-specifically tuned and defined, leading, for instance, to the complete lack of any transcription factor in the set of co-upregulated OGs. Germ cell specification and programming has been usually associated to transcriptional repression rather than activation [36, 37]. During the first steps of PGC specification in the embryo of model organisms, the retention from somatic differentiation have been associated to transcriptional repression either globally, like genome-wide repression induced by polar granule component gene (*pgc)* in *D. melanogaster* and *pie-1* in *C. elegans*, or specifically, such as the case of *blimp1* in *Mus musculus* [38, 39]. While these mechanisms are undoubtedly crucial for germline maintenance, they appear to be controlled by different and specific factors, with no homology but with similar function, distinctly tuned in the various organisms. For instance, the aforementioned master transcriptional suppressor *pgc* (upregulated in *D. melanogaster* in the present study) has no homologues outside *Drosophila*, and also sequences of the OG that included *pie-1* were observed as upregulated in the present study, but only for *C. elegans*, *D. rerio*, and *X. tropicalis*.

Once the lineage has been established and germline-specific transcription is activated, the maintenance of germline fate is apparently delegated to other mechanisms, such as chromatin remodelling and, most of all, those based on mRNA processing, i.e. post-transcriptional regulation, including the activity of many GMP genes, usually organized in perinuclear ribonucleoprotein granules [37–39]. The most studied and ubiquitous germline-related genes are indeed associated to post-transcriptional regulation and RNA-binding activities, including all well-known GMP core genes (see “Background”), with the exclusion of germline-specific Tudor proteins (that however, with their protein–protein interaction activities, are nevertheless involved in post-transcriptional regulation by their interactions with Piwi). In our study, the only 4 germline-enriched IPR codes shared by the totality of the data set corresponded to domains or families typical of RNA helicases involved in mRNA homeostasis, such as the nearly ubiquitous germline marker Vasa, that acts in post-transcriptional regulation [8]. However, OGs co-upregualted in 8 or more species (except for *piwi* and *CPEB1*), and more than half of co-enriched IPR codes and GO terms, included almost exclusively replicative signals instead of transcriptional or post-transcriptional RNA regulators.

The subset of upregulated OGs in our data set comprehended indeed many genes that encode for proteins associated to DNA replication rather than other DNA-related activities like transcription. For instance, DCTD and DUT (whose transcripts were upregulated in 8 species) are metabolic enzymes that produce dUMP (from dCMP and dUTP, respectively [40, 41]). This metabolite represents the upstream step of dTMP, a precursor of dTTP, whose metabolic end is represented by the inclusion of a thymine in the DNA molecule. The enrichment of these two genes hints for a bias toward DNA synthesis with respect to RNA synthesis, therefore toward cell replication, and they are essential for proper DNA replication by balancing metabolite composition toward dTTP production and therefore avoiding dUTP mis-incorporation in the DNA molecule [41]. Other co-upregulated gene products are directly involved in DNA replication initiation (SSRP1 and MCM4 [42, 43]), DNA replication progression (DNA2, ASF1B, NASP, and PCNA [44–47]), or chromosome segregation and nuclear division (TOP2, PLK1 [48, 49]; but also importin-alpha [50]).

While RNA processing-related IPR codes and GO terms were enriched in many of the germline-related samples of our data set (e.g. the only 4 IPR codes over-represented in all 9 species; many RNA-binding domains enriched in 6 or more species; “ribonucleoprotein complex biogenesis” shared by 8 species, and so on; see Additional file 2: Figs. S3, S4, S5), whole-length homologues were not, suggesting that what is conserved are the mechanisms, rather than the factors involved (again, with the exception of some classic GMP genes like *piwi*, *vasa*, and *nanos*, and the mRNA regulator *CPEB1*). The classic GMP genes represent the necessary components for such lineages, acting as determinants and regulators of the totipotent state, but the majority of molecular factors of the actual phenotypes could intuitively be genes associated to proliferative signals and cell cycle progression, like those that we observed as more represented in our comparative analyses. To summarize, what we retrieved was that the mostly shared germline-specific upregulated transcripts were almost exclusively involved in DNA replication, while other key mechanisms are indeed enriched, but apparently subjected to a deeper diversification, sometimes species-specific.

### The importance of replication fidelity is reflected by the enrichment of DNA repair mechanisms

Germline-related cell lineages represent crucial units for the organism evolution since they are the carriers of the genetic material in the reproductive/regenerative processes. Direct comparisons between germline and somatic mutation rates in human and mouse revealed that for both species the germline had a number of mutations per base pair per mitosis two order of magnitude lower than the somatic lineage, suggesting adaptive mechanisms to lower the mutation load in germ cells [51]. Indeed, among the products of the few genes co-upregulated in most of our species, there was also enrichment toward factors involved in DNA repair (Fig. 2A): MSH2 is involved in DNA mismatch repair [52], that corrects DNA replication errors that naturally occurs during the process [53], together with EXO1, that however has also excision functions in double-strand break (DSB) repair mechanisms [54]; FIGNL1 and RAD54-like are involved in DSB repair through homologous recombination [55, 56], a repair mechanism more frequent during S/G2 cell cycle phases, i.e. when DNA replicative processes occurs and sister templates are available [57]; lastly, ERCC2 is a core component of the complex TFIIH, that regulates transcription-based DNA repair through nucleotide excision repair (NER), a DNA replication-free repair mechanism [58] (Fig. 2). Moreover, some other previously cited co-upregulated genes code for products also involved in DNA repair, even if not as their primary function: for instance, TOP2 isoforms are contributors to DNA damage response and repair [59], while DNA2 is also involved in crucial steps of DSB repair [60], and PCNA in the initial steps of DNA resynthesis during mismatch repair [61] (Fig. 2A).

Among the different biological and evolutionary mechanisms to lower the mutation load, a higher percentage of transcripts that encode for DNA repair factors should intuitively be promoted when DNA replication fidelity is important. Interestingly, interspecific comparisons between livers of long-living and short-living vertebrates showed that the transcription of DNA repair-associated genes was significantly higher in long-living species, coupling the transcription level to the efficiency of the mechanism [62]. The importance of correct transmission of genetic information across generations, that being the result of sexual or asexual reproduction, or in regenerative processes, is probably the driver of the shared upregulated transcription that we observed in the germline-related samples of the analysed species. Moreover, it was also interesting to notice that many different repair mechanisms were represented, and not necessarily only those coupled to DNA replication, suggesting a general enrichment toward fidelity in genetic information transmission.

This DNA repair-oriented trend was also confirmed by the functional annotation analysis on domains, families, and functions. Indeed, one of the 3 GO terms related to biological processes that were enriched in germline-related samples of 6 species or more with all progressively stringent DE analysis cut-offs was “DNA repair”, that is a general term that comprehends a wide variety of heterogeneous mechanisms. However, GO terms related to different repair strategies were also represented in our analysis and enriched in most species: from those strictly associated to DNA replication, like “mismatch DNA binding” (GO terms enriched in 6 species), to those more usually associated but not restricted to it, like “DSB repair” (enriched in 7 species), to replication-free ones like “NER” (in 6 species).

The general term for “DNA repair” represented one of the most robust signals in germline-related samples, together with other multi-comprehensive terms like “DNA replication”. On this matter, an interesting observation is the fact that the only species not enriched for “DNA replication” was *C. elegans*. Probably one of the strongest possible bias drivers of the present analysis was the lack of a somatic control represented by highly proliferative tissues for all species, implying the possibility that the enrichment toward replicative and proliferative signals observed in germline-related samples was mainly led by the lack of mitotic activity in the controls. The only case for which we could exclude this potential bias was *C. elegans*, where the somatic sample was represented by proliferating embryonic somatic cells. Interestingly, the signals that emerged for this species were mostly in line with all others, supporting the fact that most of the observations could indeed be interpreted in a germline-oriented scenario. Coherently, the GO terms for “DNA replication” was not enriched in *C. elegans*, but nevertheless that for “DNA repair” was, together with all other aforementioned GO terms for different DNA repair mechanisms. Moreover, while for instance *PCNA* was not upregulated in germline-related samples of *C. elegans* (coherently with the high mitotic rate shared by the control somatic samples), all DNA repair-associated genes were (with the exclusion of *FIGNL1*; Fig. 2). This strengthens the suggestion toward a germline-related molecular phenotype biased toward DNA transmission fidelity, that is not entirely interpretable in terms of basic proliferative activities, and that might represent a selected trait to lower the mutation load in a cell type that carries the burden of genetic inheritance through generations.

## Conclusions

By comparing the transcriptional profiles of species from different phyla some conclusions could be drawn. First of all, for the whole data set, based on the estimated level of lineage-specific gene occurrence in each species, the phylostratigraphic analysis revealed that lineage-specific genes are less likely to be included among germline-related upregulated transcripts than expected by chance, both as regards phylum- and Metazoa-specific novelties. This is coherent with previous results on specific totipotent cell types and with the early origin of germline in metazoan multicellularity evolution. Here, however, we also highlighted the shared profiles between germline and some totipotent cell lineages that were formerly considered somatic (i.e. archaeocytes and neoblasts, now considered PriSCs). Transcriptional signals shared between the germline-related samples were oriented not only toward upregulation of proliferative activities (especially DNA replication and cell cycle progress), but also DNA repair, whose correct and proper course is fundamental for the genetic “responsibility” of totipotent lineages, and whose molecular factors are widely conserved across the data set. Instead, signals of either transcriptional or post-transcriptional regulation, that are more usually associated to germ cells, were not massively shared in terms of whole sequence homology, but rather in terms of enriched functions and domains, suggesting shared molecular processes but leaving proper genetic inheritance transmission as the most conserved genetic toolkit.

## Methods

### Data set

All RNA-Seq reads used in the present study were downloaded from the Short Reads Archive of NCBI (https://www.ncbi.nlm.nih.gov/sra). We searched for female germline-related samples (i.e. the lineage that maintains totipotency throughout development [36]) in metazoan RNA-Seq experiments generated through Illumina platforms with the following key-words: oocyte(s), gonad(s), egg(s), germline, germ line, germ cell(s). The search results were then filtered for experiments that included both samples belonging to exclusively germline-related tissues or cells and also any kind of somatic tissue within the same project, and contemporarily for experiments that included at least 2 biological replicates for condition. We then chose the final data set keeping an even representativeness among taxa.

The candidates belonged to 11 species: *E. fluviatilis* (Porifera), *N. vectensis* (Cnidaria), *Brachionus manjavacas* (Rotifera), *C. elegans* (Nematoda), *D. rerio* (Chordata), *X. tropicalis* (Chordata), *D. melanogaster* (Arthropoda), *Penaeus chinensis* (Arthropoda), *R. philippinarum* (Mollusca), *H. rufescens* (Mollusca), and *Eisenia fetida* (Annelida). From these, *E. fetida* (PRJNA304461) was excluded because the germline-related samples were represented by whole bodies enriched for gonads, and not only the specific tissue of interest. *P. chinensis* (PRJNA558194) was excluded during the analyses because an over-representation of stress-related signals emerged during the DE analysis, invalidating the confidence of the samples. Also *B. manjavacas* (PRJNA345262) was excluded in the course of the study because we could not retrieve any germline-related signal out of it. In facts, its samples were constituted by whole bodies against eggs, enriched in transcriptional signals related to the subsequent onset of embryogenesis: indeed, the lack of any conserved and a priori known GMP gene transcription casted shadows on the reliability of such experiment as regards the approaches and aims of the present analysis.

We also decided to include among our samples RNA-Seq reads of *S. mediterranea* neoblasts (and differentiated progeny as somatic control). These cells, together with multipotent cells of other Metazoa, have been associated to the germline since neoblasts express germline-associated signature genes, leading to theorise the existence of the GMP shared by totipotent germ cells (see “Background”). Therefore, the final data set comprehended 9 species covering 7 phyla (Table 1).

### Transcriptome assembly and differential expression

Given that RefSeq genomes were not available for all the species of our data set, we decided to uniform any kind of computational bias among our samples, and we performed a de novo transcriptome assembly for all. Assemblies were performed for each species with Trinity v2.9.0 [63] by pooling all samples together, with default parameters for read normalization. Read quality filter was performed with Trimmomatic v0.39 [64] using a sliding window size of 1/5 of the read length with a cut-off phred score of 28, and excluding all reads shorter than 2/3 of read length.

To reduce complexity, we collapsed transcripts through CD-HIT v4.8.1 [65] at 99% of identity. We then filtered the transcriptomes by keeping exclusively transcripts that had a metazoan best hit as result of a DIAMOND v2.0.6.144 search [66] against the non-redundant protein database of NCBI (10^–5^ e-value cut-off). The completeness of the filtered transcriptomes was evaluated through the BUSCO v5 set of core metazoan orthologues as implemented in the gVolante website (https://gvolante.riken.jp/index.html).

Since we were interested in coding sequences (CDSs) only, we also performed an open reading frame (ORF) prediction through TransDecoder v5.5.0 (https://github.com/TransDecoder), keeping the single best ORF for each transcript. To help inferring the most likely ORF position within the transcript, the software was also fed with a DIAMOND search against Swiss-Prot (10^−5^ e-value cut-off; [67]) and an HMMscan (HMMER v3.2.1 [68]) against Pfam-A [69]. Only transcripts with a predicted ORF were considered for the subsequent analyses.

Transcript quantification was performed for each species with Salmon v1.3.0 [70]. DE analyses were then performed with both DESeq2 [71] and edgeR [72], as implemented in the Trinity utilities package. Transcripts with a log_2_ fold change (logFC) higher than 1 in the germline-related samples (i.e. twice as abundant with respect to the control somatic samples), with a corrected p-value lower than 10^−3^ and significant for at least one analytic tool, were considered as differentially upregulated. To test the robustness of the results, we also iterated the DE analysis with higher logFC cut-off (> 1.5, and > 2) and observed the consistency in all downstream analyses (co-upregulated genes in 8 or more species; co-enriched GO terms and IPR codes in 6 or more species). Moreover, we also reran all comparative analyses by considering the intersection (rather than the union) of DE results obtained with DESeq2 and edgeR. Results presented in the main text refer to logFC > 1 and DESeq2/edgeR union (all comparisons with other cut-offs are present in Additional file 1).

### Phylostratigraphic analysis

For each species set of upregulated transcripts, we wanted to calculate the proportion of sequences that shared homology across Metazoa and the proportion of phylum-specific ones, i.e. a phylostratigraphic analysis of the germline-related upregulated transcriptomes. To do that, we downloaded 111 proteomes from online databases (covering 21 animal phyla, comprehending all those belonging to our data set species, and 4 unicellular holozoan taxonomic groups, i.e. the closest relatives to Metazoa; Additional file 2: Table S1) and ran an homology inference between them and our 9 species translated transcriptomes. The analysis was run with OrthoFinder v2.3.11 [73] with the –ultra-sensitive parameter (highest sensitivity) and all sequences that ended up within the same cluster (OrthoFinder’s OrthoGroups, or OGs) were considered homologous. An upregulated germline-related transcript was considered inter-phyletic when it shared homology with at least another sequence outside the belonging phylum. If a CDS ended up within an OG composed exclusively of intra-phyletic sequences, we considered it phylum-specific. CDSs that shared at least one homologue outside the belonging phylum were considered Metazoa-specific if no sequences of unicellular Holozoa were comprehended in their OG.

To assess whether there was any over-representation of intra-phyletic or inter-phyletic homology in the germline sequence subsets, we also calculated the phylum-specific percentage of non-upregulated genes (i.e. the rest of the translated transcriptome). Then, for each species, we calculated the ratio between the two percentages (phylum-specific percentage of germline-upregulated genes / phylum-specific percentage of non-upregulated genes). For genes that shared homology across phyla, we also calculated a similar ratio for Metazoa-specificity: genes were considered Metazoa-specific if they had no homologues in the 8 unicellular holozoan. For each species we also produced 1000 random sets of genes of sizes equal to those of germline-related upregulated ones, and calculated the phylum- and Metazoa-specifc ratios on them to assess the solidity of the method (summaries are available in Additional file 1).

### Comparative analyses

To observe whether there were any homologous CDSs upregulated in different species of our data set, we first constructed homology clusters for the whole translated transcriptomes of our 9 species. We used OrthoFinder with the same parameters for the phylostratigraphic analysis previously exposed. We used two different OrthoFinder runs for the phylostratigraphy and for the investigation on co-upregulated transcripts because of the different aims of the two analyses. For the former, a higher number of species was fundamental to avoid gene age underestimation. Indeed, one of the risks of phylostratigraphy with BLAST-based methods is the failure to find homologous sequences due to extreme sequence divergence, leading to overestimation of gene novelties. To overcome this, it is necessary to maximize phylogenetic representativeness. On the other hand, by increasing the number of species, OrthoFinder algorithm might more frequently cluster together sequences that share just partial conserved regions, therefore leading to the collapsing of clusters. While this is not a heavy problem for phylostratigraphy, because it would estimate the age of the cluster as that of a combination of specific conserved regions (that is still of biological interest), it is less convenient for the analysis of co-upregulation annotation. By running OrthoFinder on the 9 species of the data set only, we could obtain less noisy OrthoGroups that were more easily annotated and for which we could grasp the biological meaning more straightforwardly.

CDSs of different species were considered co-upregulated in germline-related samples between two species when they were significantly differentially transcribed (see previous "Methods" section) and belonged to the same OG. For OGs that comprehended sequences co-upregulated in most species (8 or more), we specifically annotated the sequence content by BLAST searches based on the sequences of *C. elegans*, *D. rerio*, *D. melanogaster*, and *X. tropicalis*, since for these models the confidences of online annotations are high, and functional data are available. For other OGs, we counted the number of times that all possible combinations of species ended up within the same germline-related OGs. In this way we could count how many times each combination of species had a shared set of germline-related upregulated CDSs, and we calculated the deviation from expected random distributions with the UpSetR R package as implemented online (https://vcg.github.io/upset/ [74]).

We also ran InterProScan v5.45.80 [75] on the whole translated transcriptomes of all 9 species, annotating for each sequence the associated GO terms and IPR codes. We performed a GO term enrichment analysis (topGO package on R [76]) to observe which biological processes and molecular functions were significantly enriched in each species germline-related samples. Results presented refer to the “classic” algorithm of topGO. We also reiterated the enrichment analyses with the “elim” and “weight01” topGO algorithms, that take into account also the hierarchical structure of GO terms (all results are presented in Additional file 1). Only those GO terms that were annotated in germline-related transcripts at least twice as much as randomly expected were considered. We then looked at such germline-related enriched GO terms shared by at least two-thirds of our data set (i.e. at least in 6 species). Visualization of semantically similar GO terms was performed on the ReViGO server with a collapsing threshold SimRel value of 0.9 (http://revigo.irb.hr/ [77]).

A similar, but not overlapping, analysis was performed with IPR codes. For each IPR code of the InterProScan database (that are annotation codes corresponding to both domains, motifs, and protein families), we counted the species-specific number of CDSs respectively annotated in the germline-related subset and in the full translated transcriptome. When an IPR code was annotated exclusively among germline-related CDSs, we considered it as enriched toward germline-related samples. For all other IPR codes, we tested whether they were significantly enriched. We performed an odds ratio test (*odds.ratio* test in R, *questionr* package), that associates a p-value to the comparison of two ratios: the ratio of appearance of each IPR code in germline-related sequences was compared to the ratio in the whole translated transcriptome. A p-value lower than 0.05 for the test meant that the IPR code was enriched in germline-related samples. Comparative analyses were performed considering IPR codes that were over-represented in more than two-thirds of the species.

## Supplementary Information

**Additional file 1: **Zipped directory comprehending more detailed information on the species-specific transcriptomic analyses (transcriptomic statistics, read counts, fasta of upregulated transcripts, GO/IPR enrichment analyses, ratios bootstrap iterations) and extensive results of the comparative analyses (co-upregulated transcripts, co-enriched GO/IPR, comparisons of different cut-off iterations)

**Additional file 2: Table S1.** Species in the data set and accession codes of Genome assemblies. When the proteome was retrieved from an online source different from NCBI, the whole accession link is present. Phyla are in alphabetical order, with the four non-Metazoa phyla at the bottom of the table. **Figure S1.** GMP genes-associated IPR codes in germline-related samples. The table represents presence (light blue) or absence (light grey) in different species (columns) of IPR codes annotated for some representative GMP genes (whose transcript upregulation is depicted in Figure 2 of the main text). Considering associated domains and families rather than whole sequence homology improved the signal: for instance, virtually all domains belonging to vasa are biased in germline-related samples of all species, even if we could not observe vasa homologue upregulation for C. elegans and H. rufescens (Fig. 2 of the main text). **Figure S2.** Counts of co-upregulated OGs for all combinations of species. Each row represents the number of OGs that included upregulated germline-related transcripts in a precise number of species (from 2 to 9). For example, first row: 1803 OGs included germline-related upregulated sequences belonging to 2 species only (counting any possible 2-species combination). On the right the deviation from expected random distributions for the combinations of the corresponding number of species is reported: positive deviation from expectation is depicted in blue, negative deviation in red. For instance: the number of observed co-upregulated OGs in 4 species (any 4 species and only 4 species) was lower than expected; the number of observed co-upregulated OGs in 8 species (any 8 species and only 8 species) was higher than expected. **Figure S3.** Co-enriched GO terms (Biological Processes) in 6 or more species. The table represents presence (light blue) or absence (light grey) in different species (columns) of GO terms enriched in more than 2/3 of the data set. **Figure S4.** Co-enriched GO terms (Molecular Functions) in 6 or more species. The table represents presence (light blue) or absence (light grey) in different species (columns) of GO terms enriched in more than 2/3 of the data set. **Figure S5.** Co-enriched IPR codes in 6 species. The table represents presence (light blue) or absence (light grey) in different species (columns). Code annotation that refer to strictly DNA-related or proliferative activities are highlighted in bold. Codes associated to DNA repair are highlighted in red.

## Data Availability

The datasets used and analysed during the current study are available from the corresponding author on reasonable request.
