# Supplementary material for: Germline-related molecular phenotype in Metazoa: conservation and innovation highlighted by comparative transcriptomics
Source: EvoDevo. 2023 Jan 30;14:2. doi: 10.1186/s13227-022-00207-3 (PMC9885605; doi:10.1186/s13227-022-00207-3)
Supplement: Supplementary file 1 — Additional file 1: Zipped directory comprehending more detailed information on the species-specific transcriptomic analyses (transcriptomic statistics, read counts, fasta of upregulated transcripts, GO/IPR enrichment analyses, ratios bootstrap iterations) and extensive results of the comparative analyses (co-upregulated transcripts, co-enriched GO/IPR, comparisons of different cut-off iterations) [file 13227_2022_207_MOESM1_ESM.zip › SUPPLEMENTARY_MATERIAL/README.docx]

All results present in this directory refer to the logFC>1 cut-off, and considering the union of DESeq2/edgeR. For comprehensive results of more stringent cut-offs, please contact giovanni.piccinini@unibo.it

Comparative analysis (main directory):

- Transcriptomic_statistics.xlsx: summary statistics for all species
- Co-enriched* files: for each GO term or IPR code (each line), in the columns are the species for which that term was significantly enriched in germline-related samples (see Methods for cut-offs)
- Differentially_transcribed_shared_orthologues_logFC1.txt: transcripts upregulated in the germline-related samples of each species ordered by belonging OrthoFinder’s OrthoGroup
- Cross_cutoffs_comparisons.xlsc: summary of the robustness of gene/GO/IPR results through the different cut-offs (see Methods)

Species-specific analyses (in each species directory):

- PCA_logt-counts.pdf: PCA of the log-transformed counts
- DESeq2-edgeR_Venn: Venn diagram showing the relationships between the two methods to calculate differential transcription (DESeq2=blue; edgeR=red)
- Vplot*: Volcano plots for both DE methods to visualize the extent of DE transcripts
- germline_upregulated_transcripts.fasta: fasta file of the upregulated transcripts
- salmon.isoform.counts.matrix*: raw DE outputs of both Methods
- topGO*: topGO results for all algorithms (“classic”, “elim”, “weight01”), for both biological processes and molecular functions
- IPR_counts_odds.txt: IPR counts for whole transcriptome, for the subset of germline-upregulated transcripts, and the odds ratio results.
- 1000sets_ratios: density plots of the phylum-specific ratio and metazoa-specific ratio of 1000 randoms sets of genes of size equal to the subset of germline-related upregulated genes. The red line in each plot represents the ratio of the germline-related upregulated set of gene (those discussed in the main text)
