# Supplementary figures and images for: Germline-related molecular phenotype in Metazoa: conservation and innovation highlighted by comparative transcriptomics
Source: EvoDevo. 2023 Jan 30;14:2. doi: 10.1186/s13227-022-00207-3 (PMC9885605; doi:10.1186/s13227-022-00207-3)

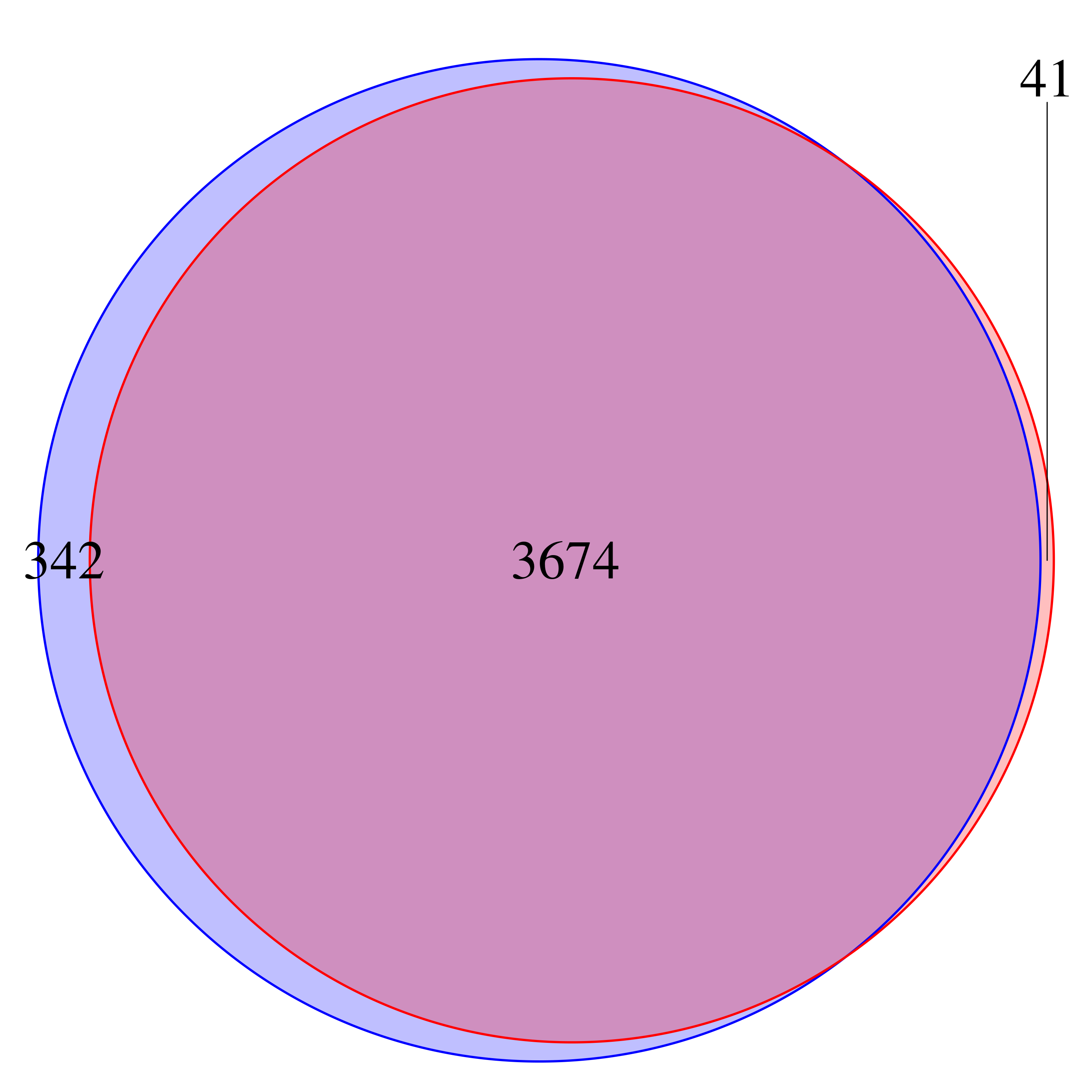

Supplement: Supplementary file 1 — Additional file 1: Zipped directory comprehending more detailed information on the species-specific transcriptomic analyses (transcriptomic statistics, read counts, fasta of upregulated transcripts, GO/IPR enrichment analyses, ratios bootstrap iterations) and extensive results of the comparative analyses (co-upregulated transcripts, co-enriched GO/IPR, comparisons of different cut-off iterations) [file 13227_2022_207_MOESM1_ESM.zip › SUPPLEMENTARY_MATERIAL/Schmidtea_mediterranea/Sme_DESeq2-edgeR_Venn.png]

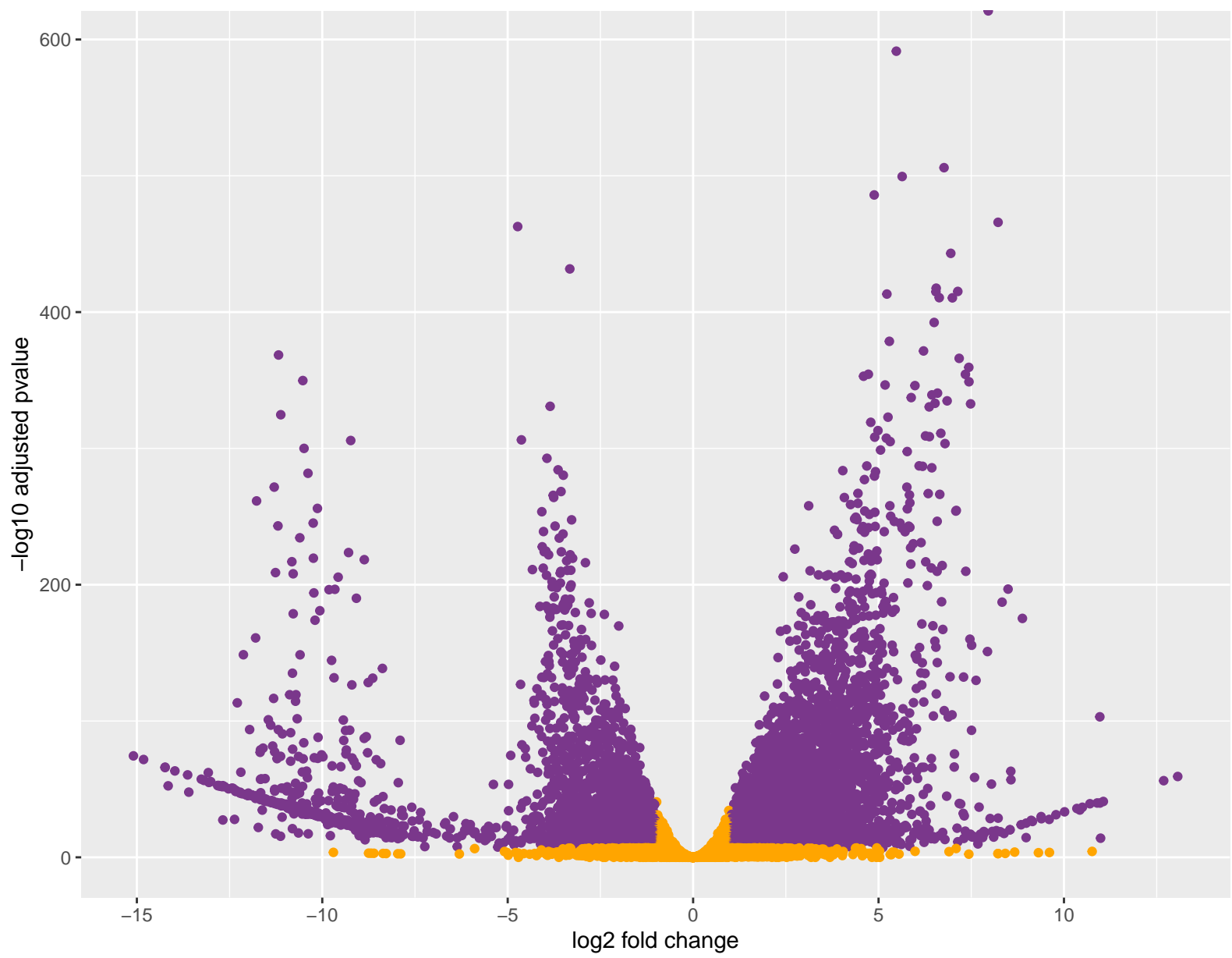

Supplement: Supplementary file 1 — Additional file 1: Zipped directory comprehending more detailed information on the species-specific transcriptomic analyses (transcriptomic statistics, read counts, fasta of upregulated transcripts, GO/IPR enrichment analyses, ratios bootstrap iterations) and extensive results of the comparative analyses (co-upregulated transcripts, co-enriched GO/IPR, comparisons of different cut-off iterations) [file 13227_2022_207_MOESM1_ESM.zip › SUPPLEMENTARY_MATERIAL/Schmidtea_mediterranea/Sme_Vplot_DESEq2.pdf]

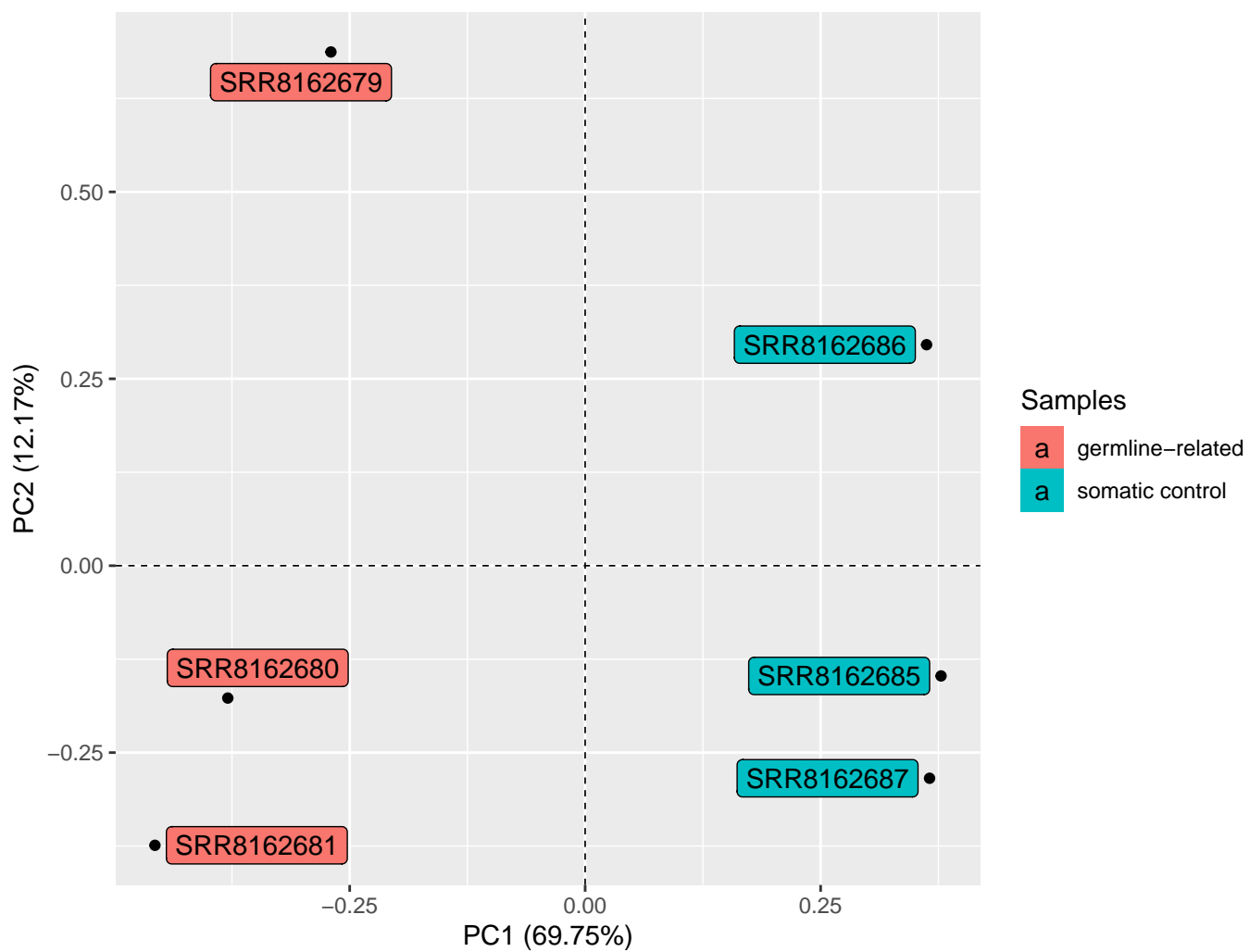

Supplement: Supplementary file 1 — Additional file 1: Zipped directory comprehending more detailed information on the species-specific transcriptomic analyses (transcriptomic statistics, read counts, fasta of upregulated transcripts, GO/IPR enrichment analyses, ratios bootstrap iterations) and extensive results of the comparative analyses (co-upregulated transcripts, co-enriched GO/IPR, comparisons of different cut-off iterations) [file 13227_2022_207_MOESM1_ESM.zip › SUPPLEMENTARY_MATERIAL/Schmidtea_mediterranea/Sme_PCA_logt-counts.pdf]

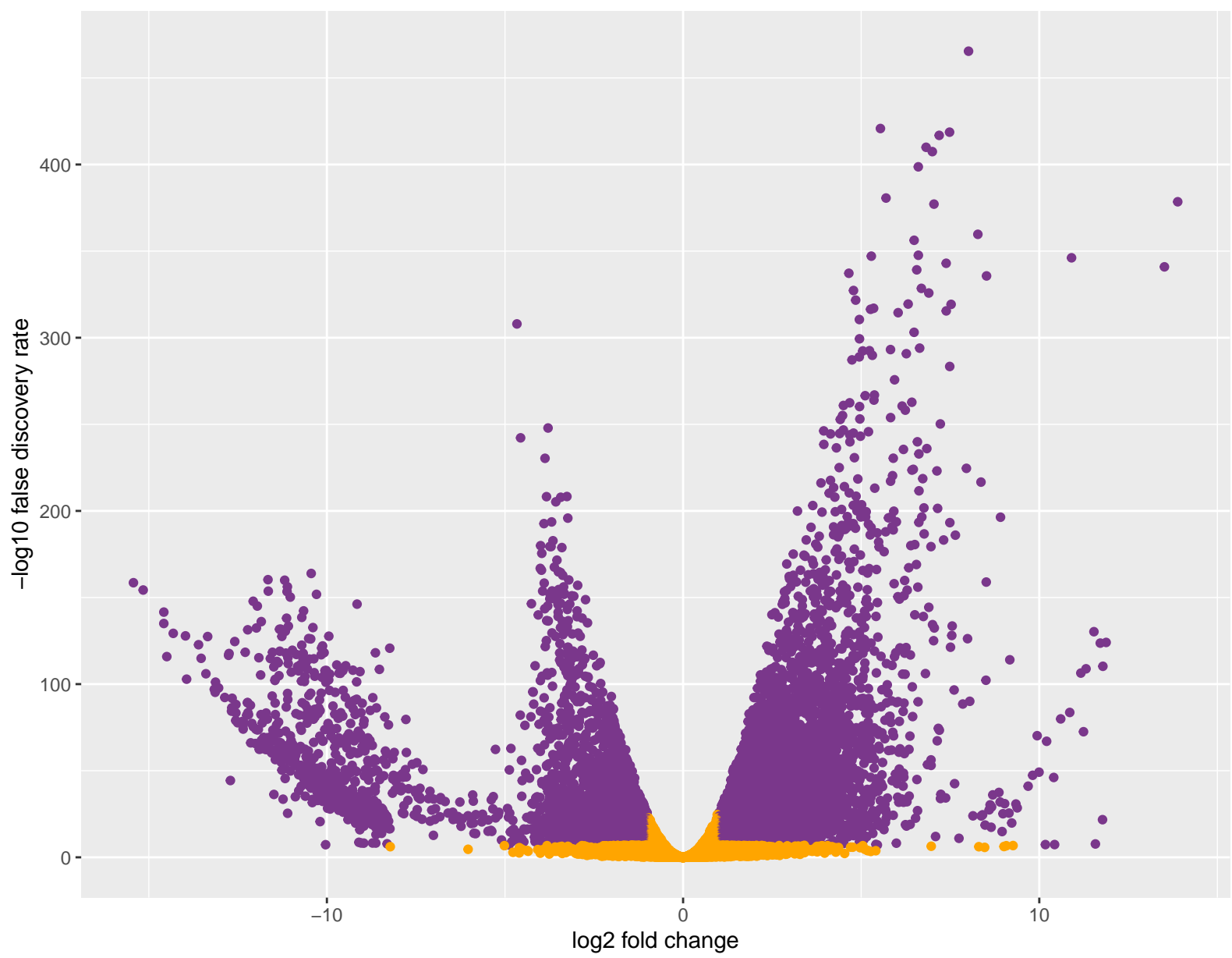

Supplement: Supplementary file 1 — Additional file 1: Zipped directory comprehending more detailed information on the species-specific transcriptomic analyses (transcriptomic statistics, read counts, fasta of upregulated transcripts, GO/IPR enrichment analyses, ratios bootstrap iterations) and extensive results of the comparative analyses (co-upregulated transcripts, co-enriched GO/IPR, comparisons of different cut-off iterations) [file 13227_2022_207_MOESM1_ESM.zip › SUPPLEMENTARY_MATERIAL/Schmidtea_mediterranea/Sme_Vplot_edgeR.pdf]

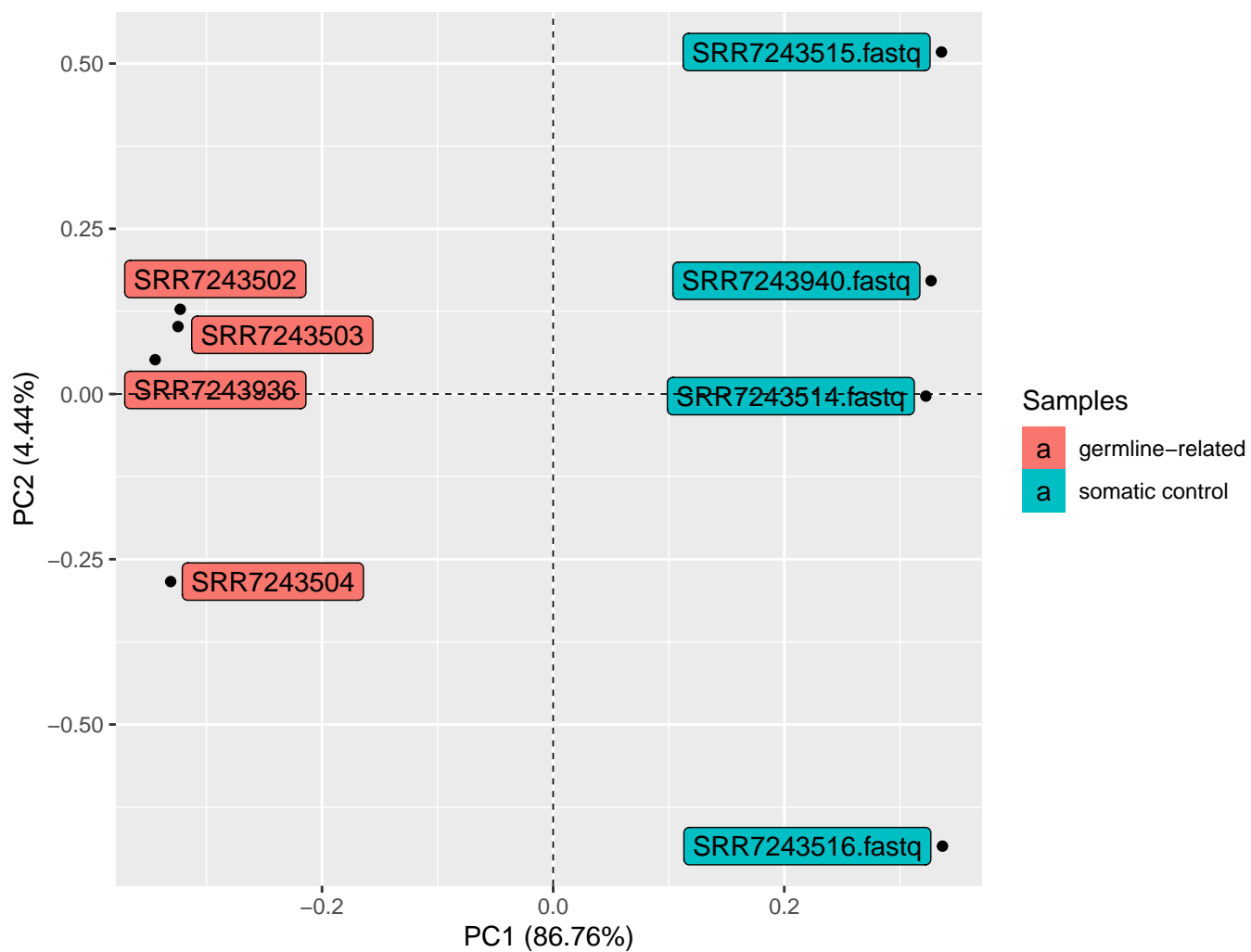

Supplement: Supplementary file 1 — Additional file 1: Zipped directory comprehending more detailed information on the species-specific transcriptomic analyses (transcriptomic statistics, read counts, fasta of upregulated transcripts, GO/IPR enrichment analyses, ratios bootstrap iterations) and extensive results of the comparative analyses (co-upregulated transcripts, co-enriched GO/IPR, comparisons of different cut-off iterations) [file 13227_2022_207_MOESM1_ESM.zip › SUPPLEMENTARY_MATERIAL/Drosophila_melanogaster/Dme_PCA_logt-counts.pdf]

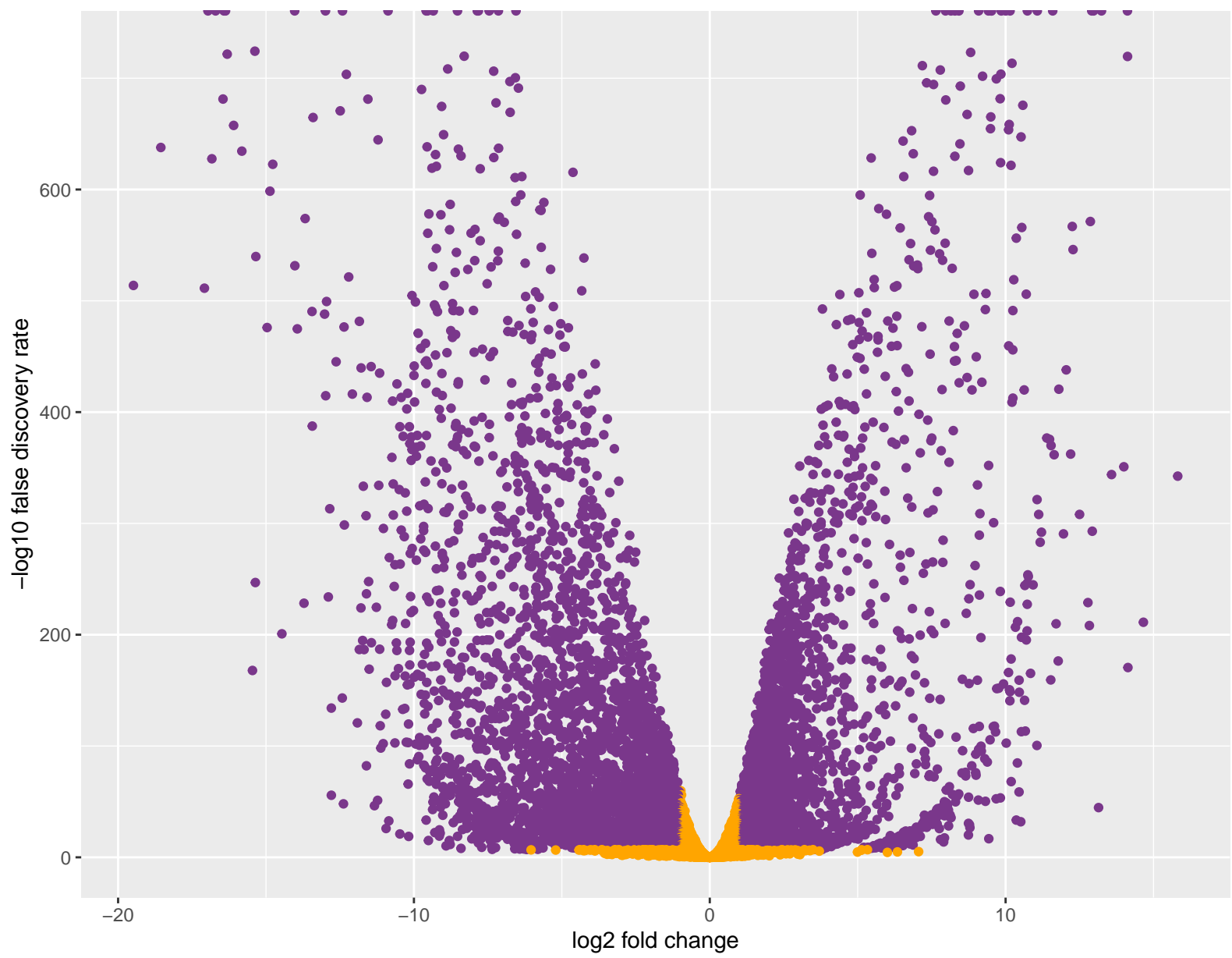

Supplement: Supplementary file 1 — Additional file 1: Zipped directory comprehending more detailed information on the species-specific transcriptomic analyses (transcriptomic statistics, read counts, fasta of upregulated transcripts, GO/IPR enrichment analyses, ratios bootstrap iterations) and extensive results of the comparative analyses (co-upregulated transcripts, co-enriched GO/IPR, comparisons of different cut-off iterations) [file 13227_2022_207_MOESM1_ESM.zip › SUPPLEMENTARY_MATERIAL/Drosophila_melanogaster/Dme_Vplot_edgeR.pdf]

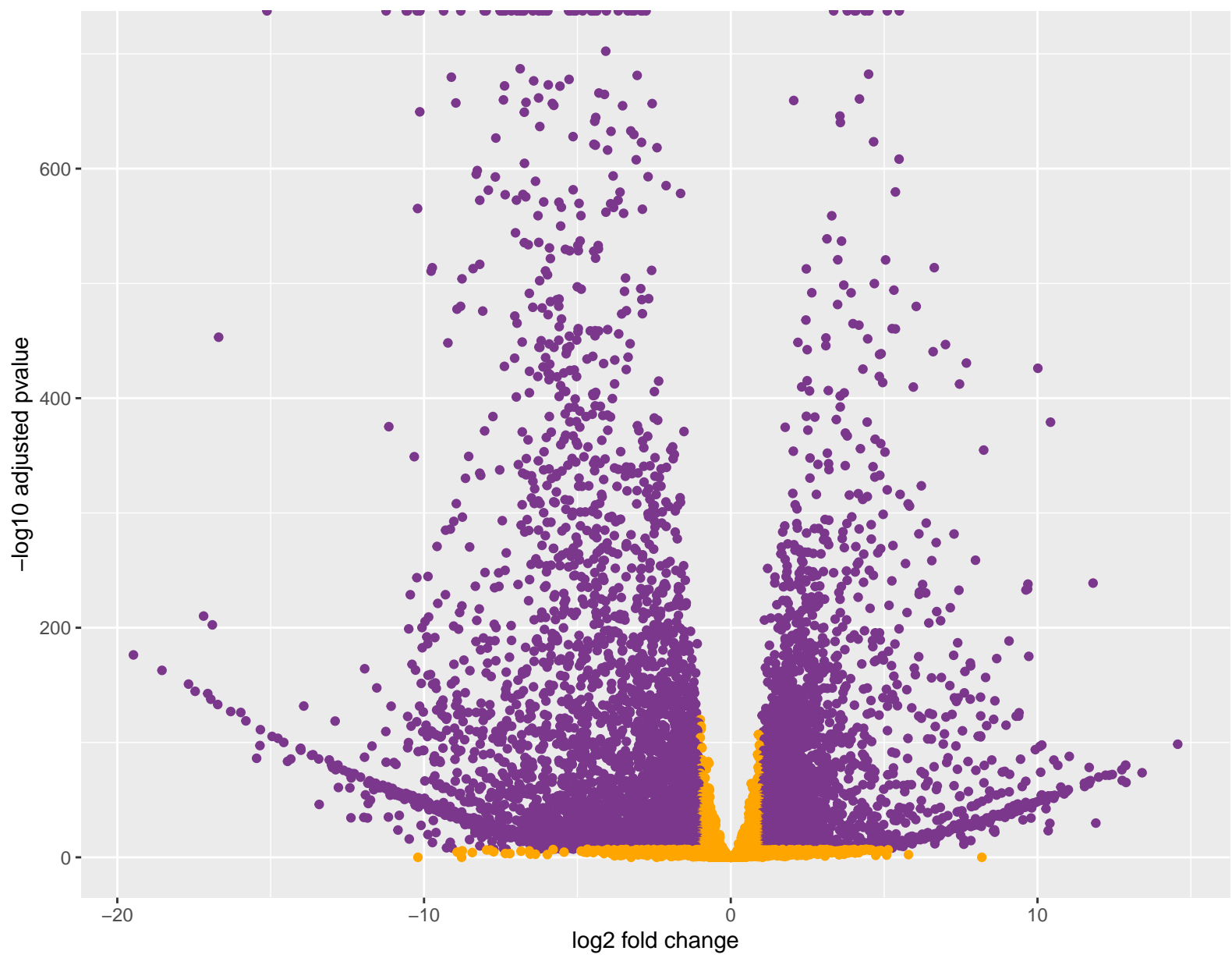

Supplement: Supplementary file 1 — Additional file 1: Zipped directory comprehending more detailed information on the species-specific transcriptomic analyses (transcriptomic statistics, read counts, fasta of upregulated transcripts, GO/IPR enrichment analyses, ratios bootstrap iterations) and extensive results of the comparative analyses (co-upregulated transcripts, co-enriched GO/IPR, comparisons of different cut-off iterations) [file 13227_2022_207_MOESM1_ESM.zip › SUPPLEMENTARY_MATERIAL/Drosophila_melanogaster/Dme_Vplot_DESEq2.pdf]

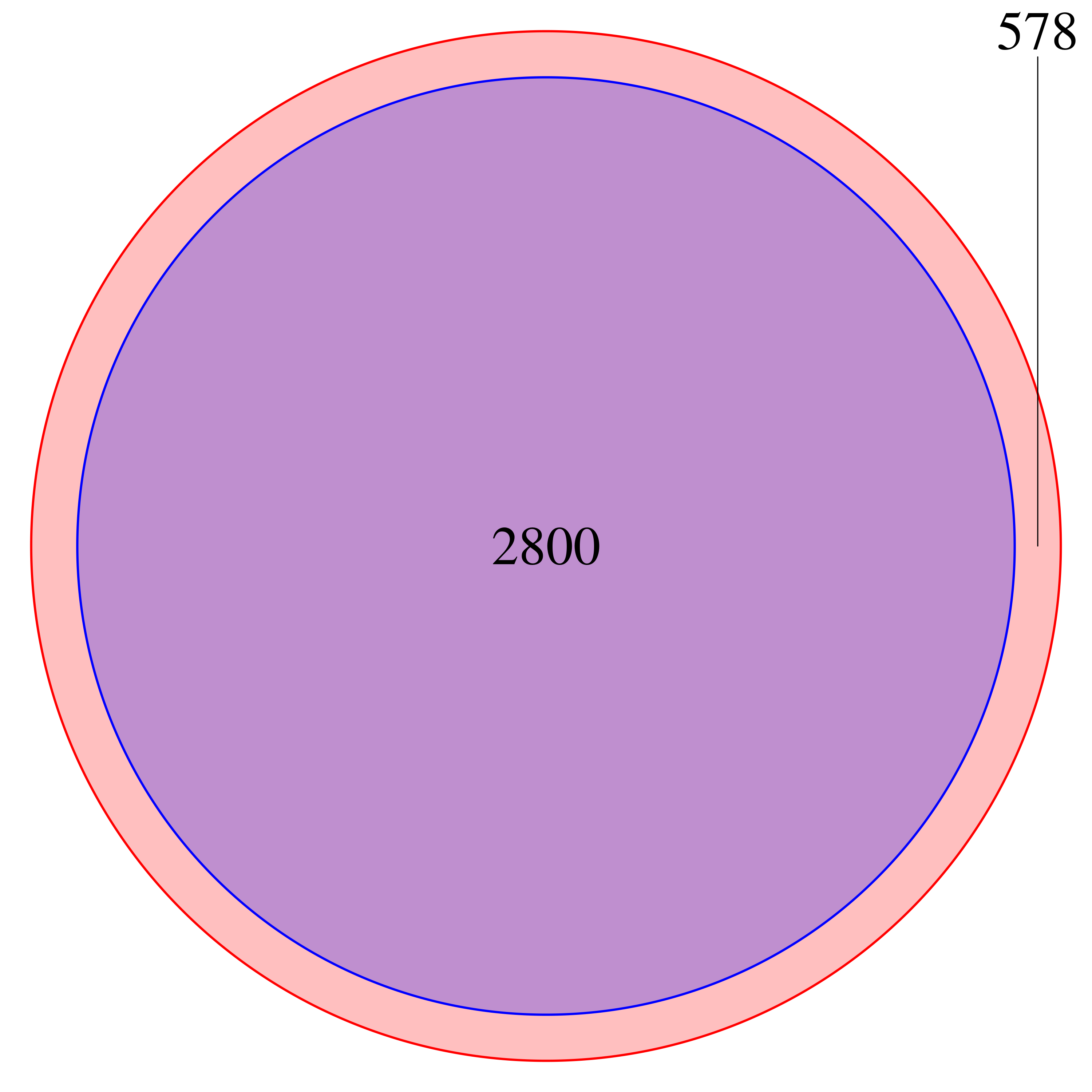

Supplement: Supplementary file 1 — Additional file 1: Zipped directory comprehending more detailed information on the species-specific transcriptomic analyses (transcriptomic statistics, read counts, fasta of upregulated transcripts, GO/IPR enrichment analyses, ratios bootstrap iterations) and extensive results of the comparative analyses (co-upregulated transcripts, co-enriched GO/IPR, comparisons of different cut-off iterations) [file 13227_2022_207_MOESM1_ESM.zip › SUPPLEMENTARY_MATERIAL/Drosophila_melanogaster/Dme_DESeq2-edgeR_Venn.png]

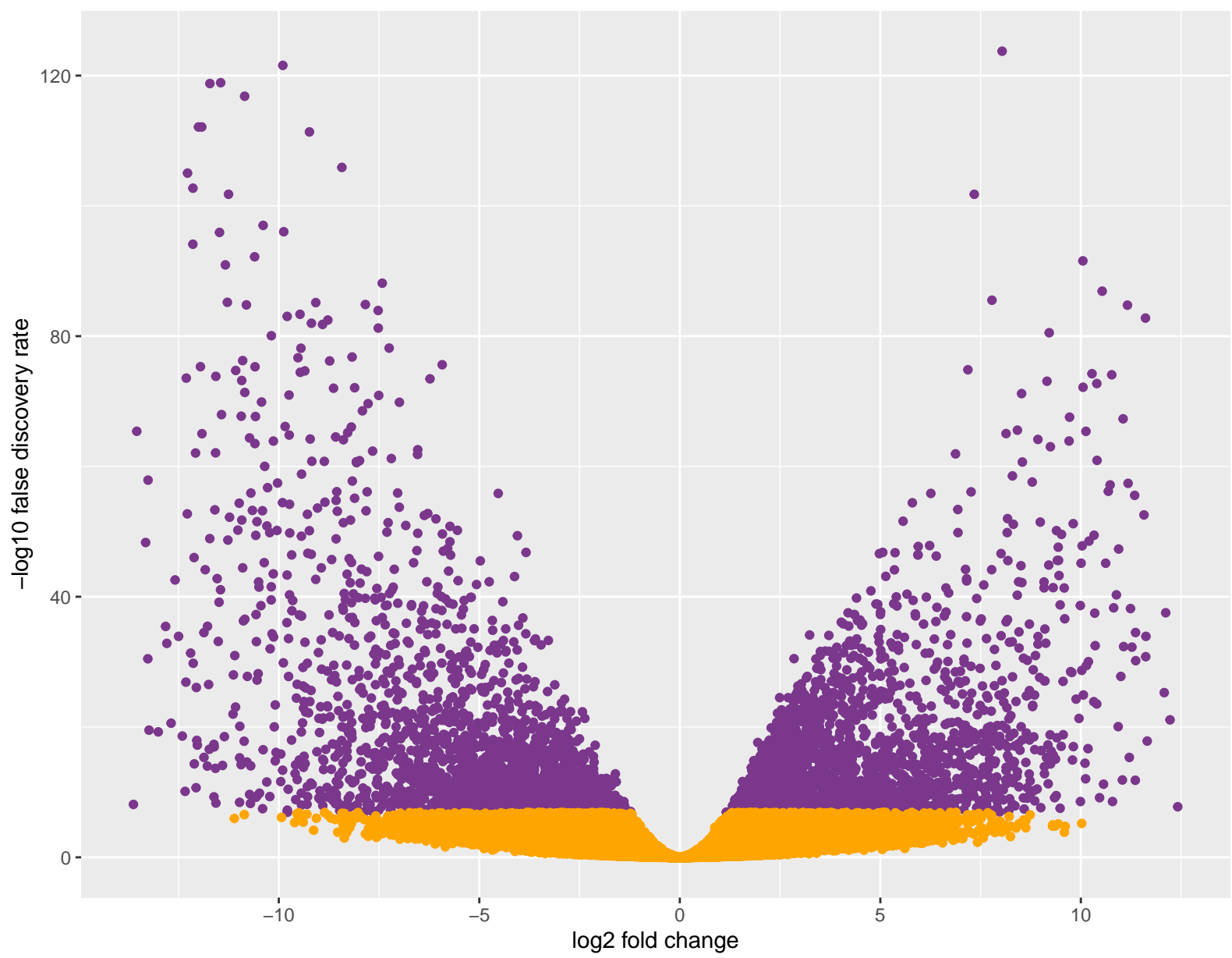

Supplement: Supplementary file 1 — Additional file 1: Zipped directory comprehending more detailed information on the species-specific transcriptomic analyses (transcriptomic statistics, read counts, fasta of upregulated transcripts, GO/IPR enrichment analyses, ratios bootstrap iterations) and extensive results of the comparative analyses (co-upregulated transcripts, co-enriched GO/IPR, comparisons of different cut-off iterations) [file 13227_2022_207_MOESM1_ESM.zip › SUPPLEMENTARY_MATERIAL/Ruditapes_philippinarum/Rph_Vplot_edgeR.pdf]

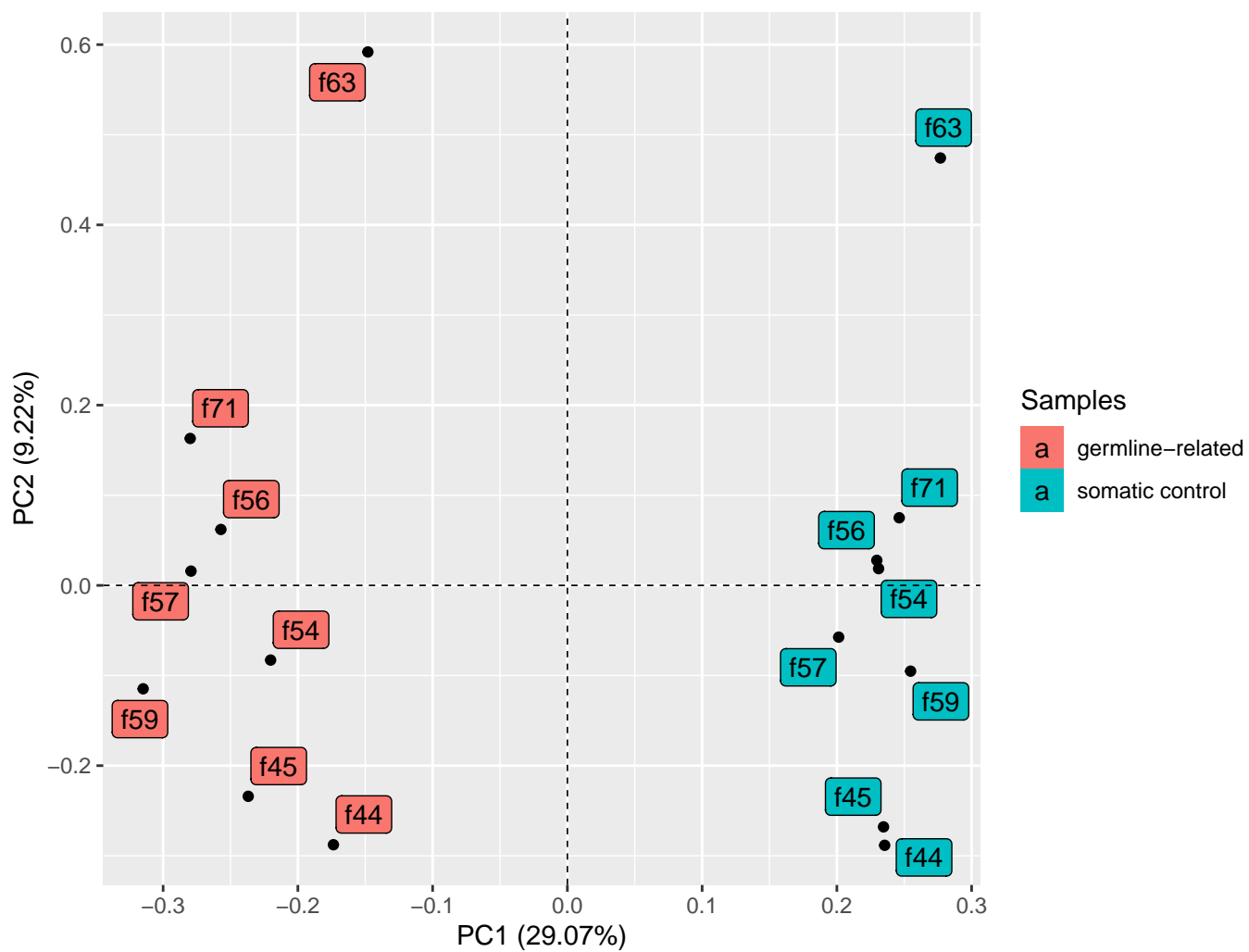

Supplement: Supplementary file 1 — Additional file 1: Zipped directory comprehending more detailed information on the species-specific transcriptomic analyses (transcriptomic statistics, read counts, fasta of upregulated transcripts, GO/IPR enrichment analyses, ratios bootstrap iterations) and extensive results of the comparative analyses (co-upregulated transcripts, co-enriched GO/IPR, comparisons of different cut-off iterations) [file 13227_2022_207_MOESM1_ESM.zip › SUPPLEMENTARY_MATERIAL/Ruditapes_philippinarum/Rph_PCA_logt-counts.pdf]

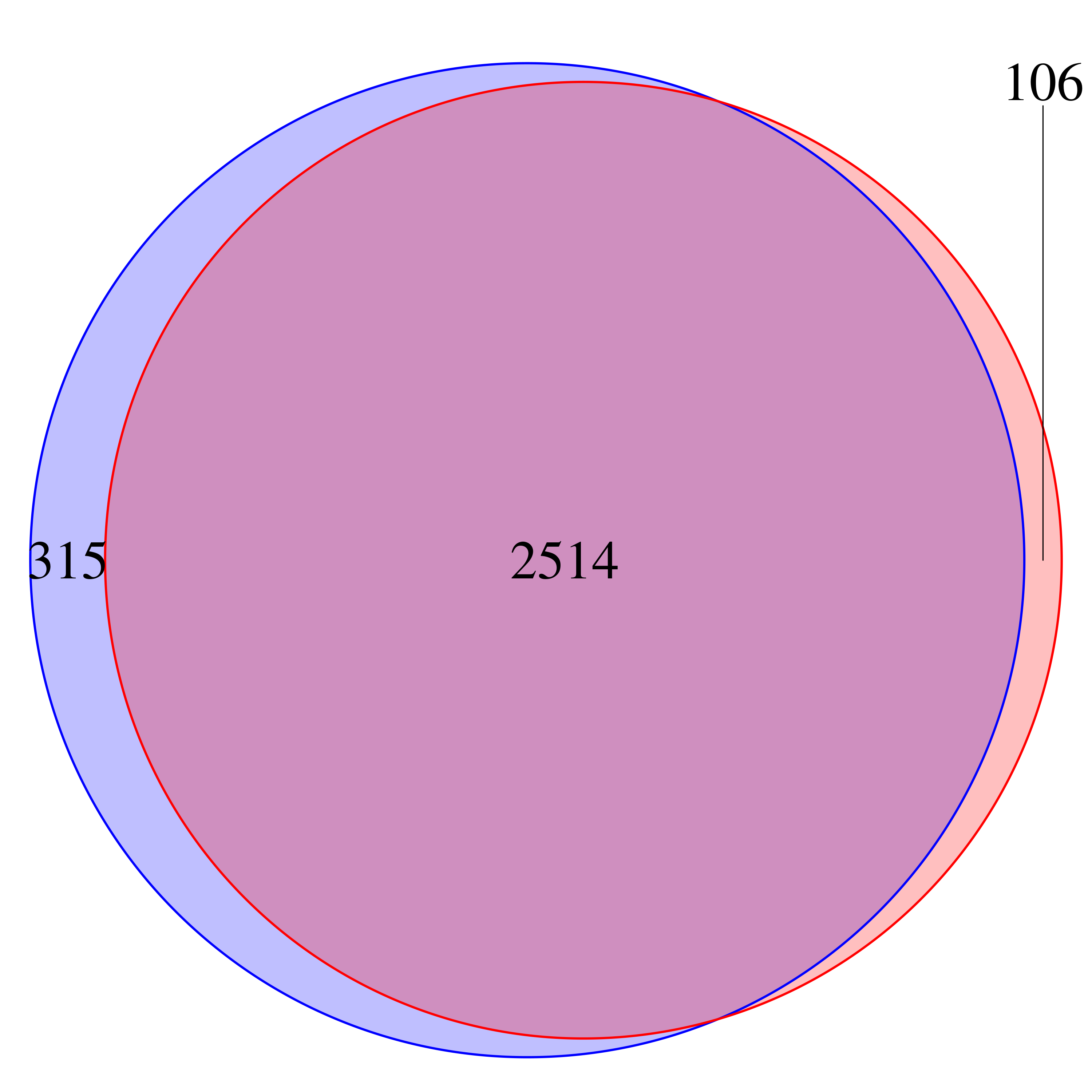

Supplement: Supplementary file 1 — Additional file 1: Zipped directory comprehending more detailed information on the species-specific transcriptomic analyses (transcriptomic statistics, read counts, fasta of upregulated transcripts, GO/IPR enrichment analyses, ratios bootstrap iterations) and extensive results of the comparative analyses (co-upregulated transcripts, co-enriched GO/IPR, comparisons of different cut-off iterations) [file 13227_2022_207_MOESM1_ESM.zip › SUPPLEMENTARY_MATERIAL/Ruditapes_philippinarum/Rph_DESeq2-edgeR_Venn.png]

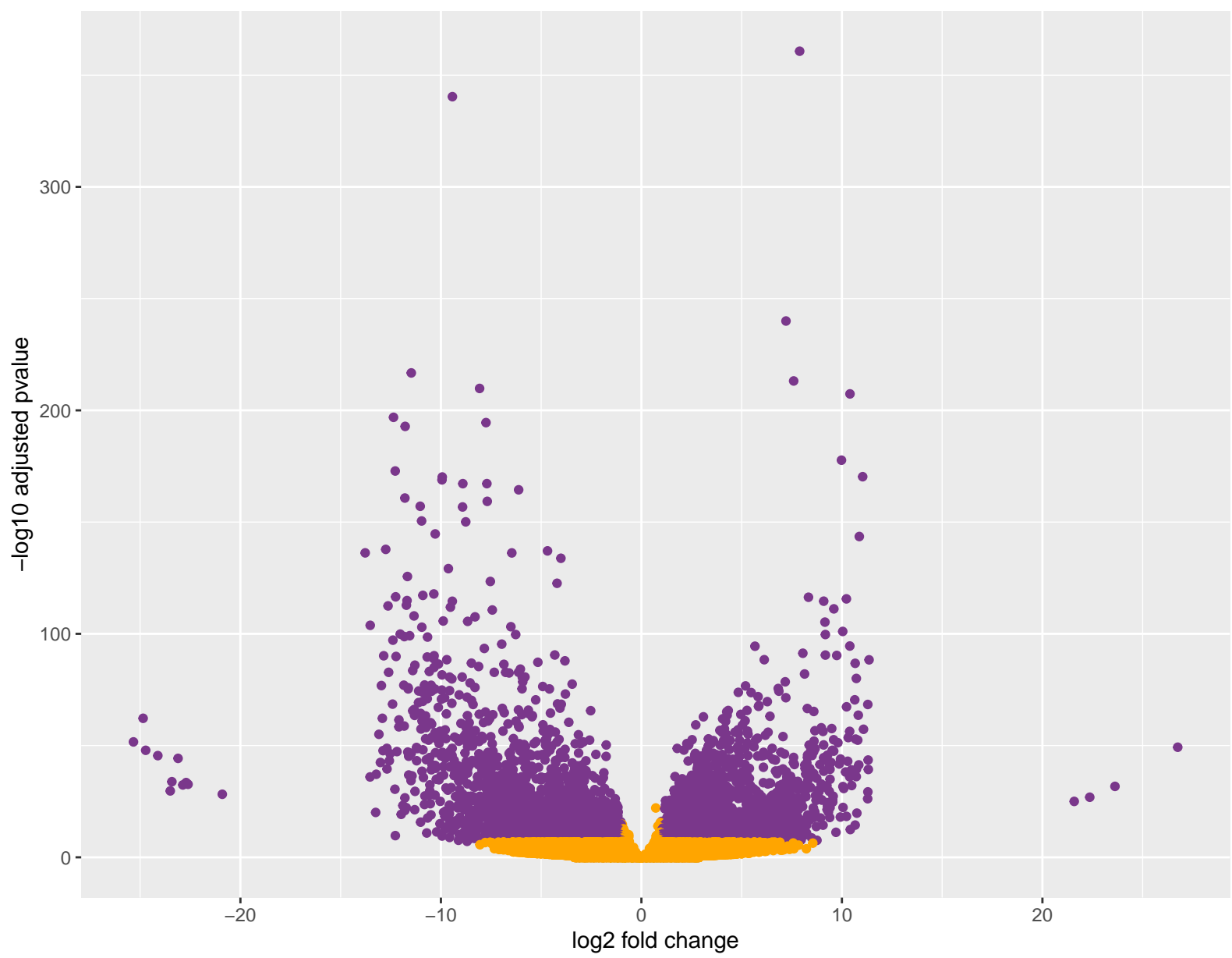

Supplement: Supplementary file 1 — Additional file 1: Zipped directory comprehending more detailed information on the species-specific transcriptomic analyses (transcriptomic statistics, read counts, fasta of upregulated transcripts, GO/IPR enrichment analyses, ratios bootstrap iterations) and extensive results of the comparative analyses (co-upregulated transcripts, co-enriched GO/IPR, comparisons of different cut-off iterations) [file 13227_2022_207_MOESM1_ESM.zip › SUPPLEMENTARY_MATERIAL/Ruditapes_philippinarum/Rph_Vplot_DESEq2.pdf]

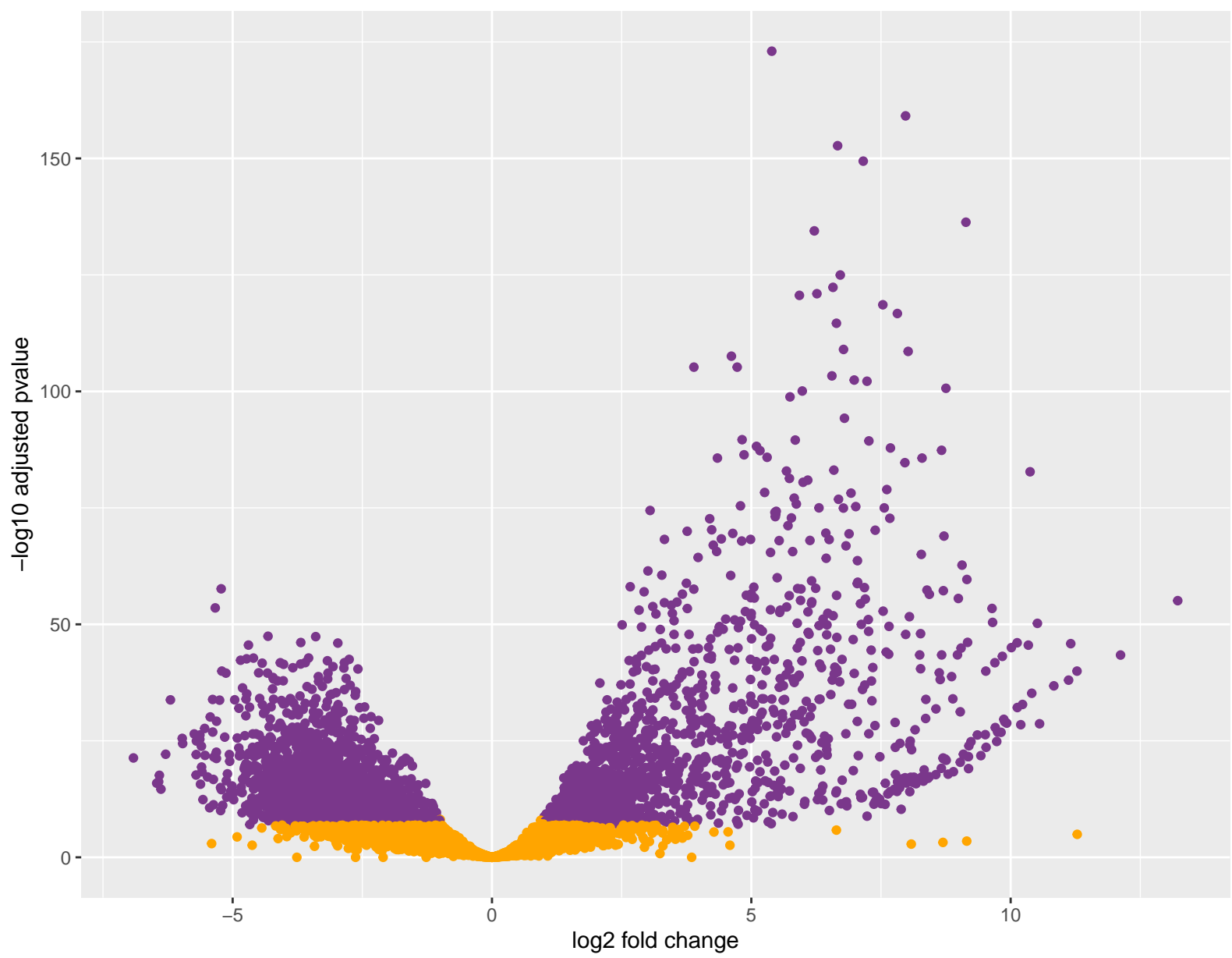

Supplement: Supplementary file 1 — Additional file 1: Zipped directory comprehending more detailed information on the species-specific transcriptomic analyses (transcriptomic statistics, read counts, fasta of upregulated transcripts, GO/IPR enrichment analyses, ratios bootstrap iterations) and extensive results of the comparative analyses (co-upregulated transcripts, co-enriched GO/IPR, comparisons of different cut-off iterations) [file 13227_2022_207_MOESM1_ESM.zip › SUPPLEMENTARY_MATERIAL/Caenorhabditis_elegans/Cel_Vplot_DESEq2.pdf]

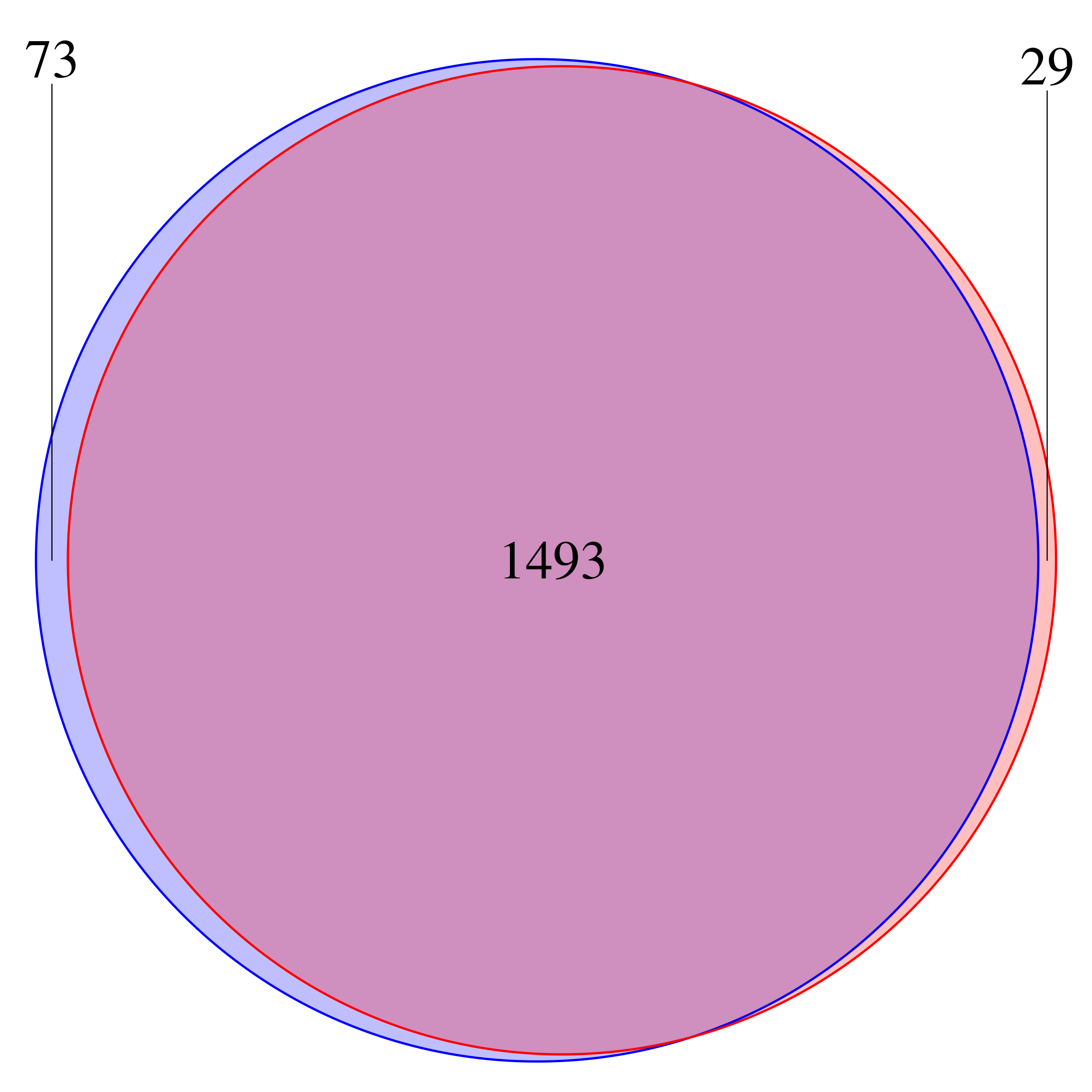

Supplement: Supplementary file 1 — Additional file 1: Zipped directory comprehending more detailed information on the species-specific transcriptomic analyses (transcriptomic statistics, read counts, fasta of upregulated transcripts, GO/IPR enrichment analyses, ratios bootstrap iterations) and extensive results of the comparative analyses (co-upregulated transcripts, co-enriched GO/IPR, comparisons of different cut-off iterations) [file 13227_2022_207_MOESM1_ESM.zip › SUPPLEMENTARY_MATERIAL/Caenorhabditis_elegans/Cel_DESeq2-edgeR_Venn.png]

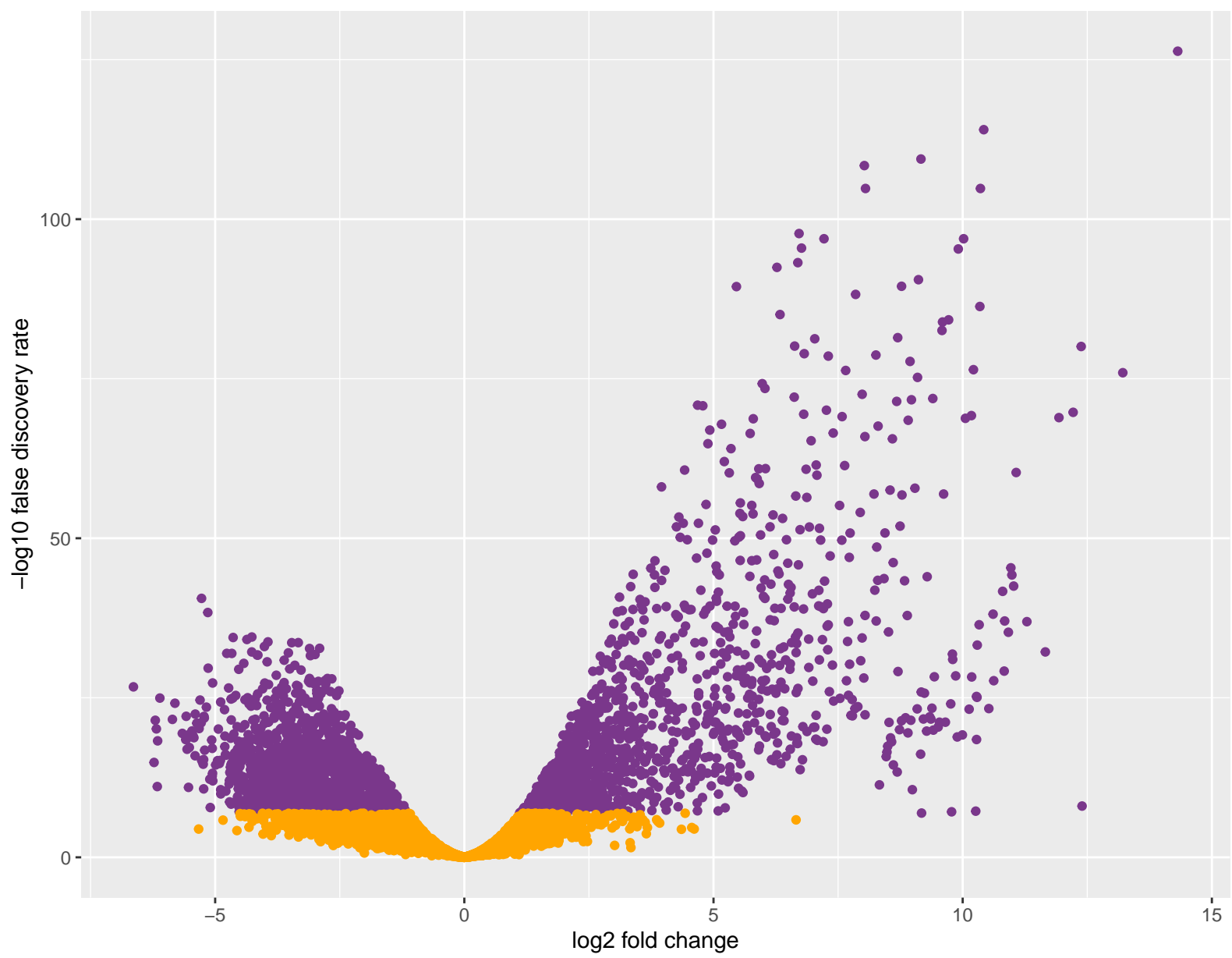

Supplement: Supplementary file 1 — Additional file 1: Zipped directory comprehending more detailed information on the species-specific transcriptomic analyses (transcriptomic statistics, read counts, fasta of upregulated transcripts, GO/IPR enrichment analyses, ratios bootstrap iterations) and extensive results of the comparative analyses (co-upregulated transcripts, co-enriched GO/IPR, comparisons of different cut-off iterations) [file 13227_2022_207_MOESM1_ESM.zip › SUPPLEMENTARY_MATERIAL/Caenorhabditis_elegans/Cel_Vplot_edgeR.pdf]

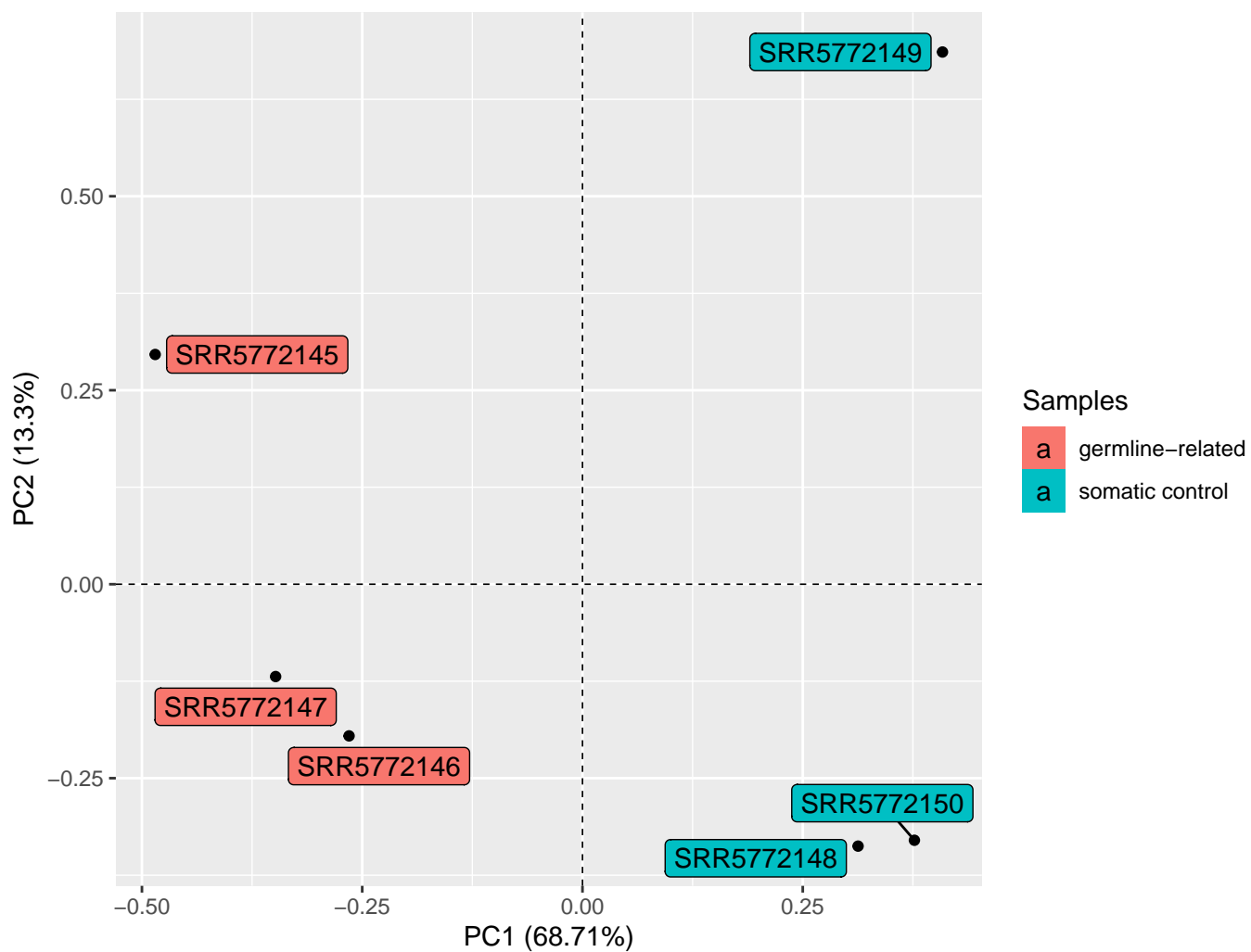

Supplement: Supplementary file 1 — Additional file 1: Zipped directory comprehending more detailed information on the species-specific transcriptomic analyses (transcriptomic statistics, read counts, fasta of upregulated transcripts, GO/IPR enrichment analyses, ratios bootstrap iterations) and extensive results of the comparative analyses (co-upregulated transcripts, co-enriched GO/IPR, comparisons of different cut-off iterations) [file 13227_2022_207_MOESM1_ESM.zip › SUPPLEMENTARY_MATERIAL/Caenorhabditis_elegans/Cel_PCA_logt-counts.pdf]

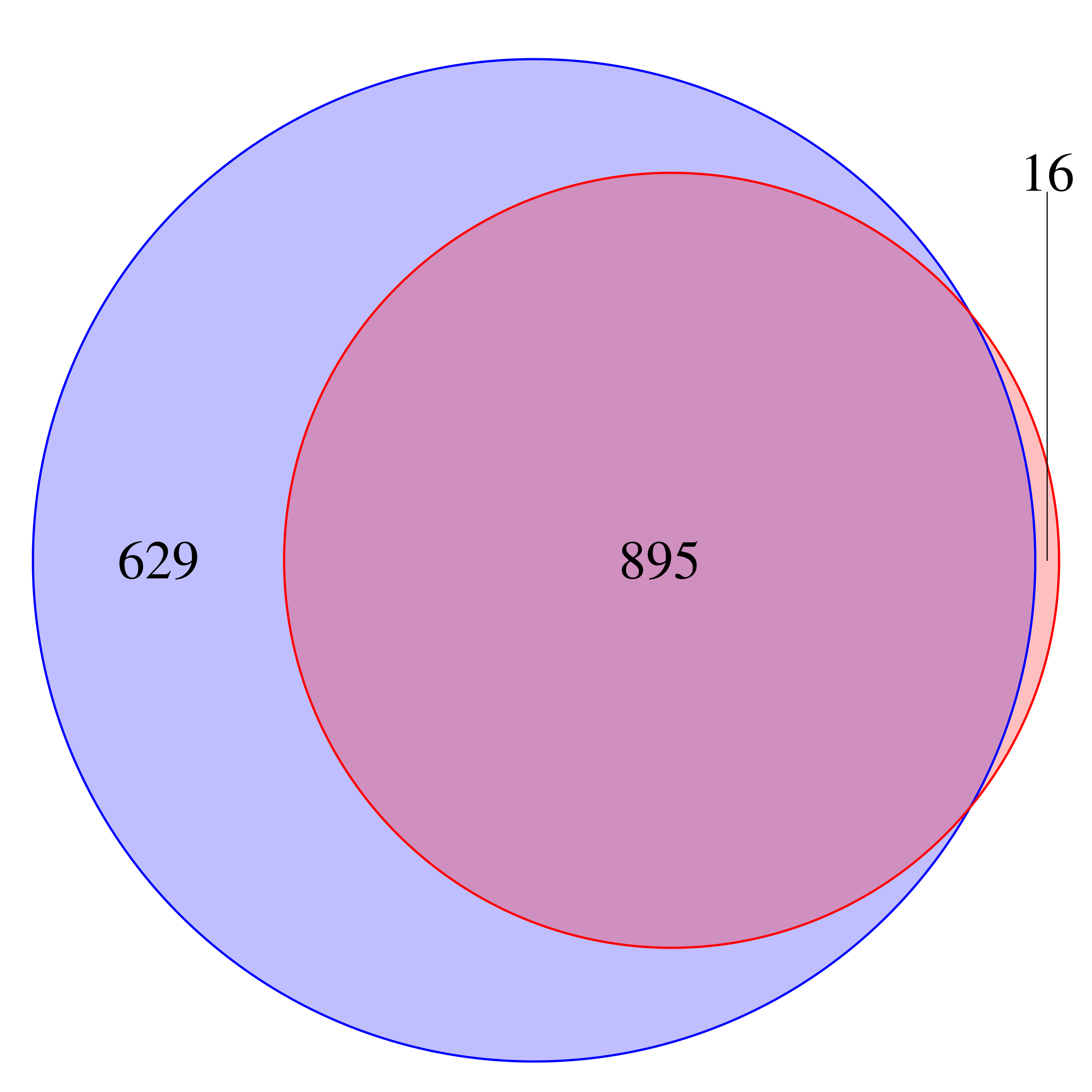

Supplement: Supplementary file 1 — Additional file 1: Zipped directory comprehending more detailed information on the species-specific transcriptomic analyses (transcriptomic statistics, read counts, fasta of upregulated transcripts, GO/IPR enrichment analyses, ratios bootstrap iterations) and extensive results of the comparative analyses (co-upregulated transcripts, co-enriched GO/IPR, comparisons of different cut-off iterations) [file 13227_2022_207_MOESM1_ESM.zip › SUPPLEMENTARY_MATERIAL/Haliotis_rufescens/Hru_DESeq2-edgeR_Venn.png]

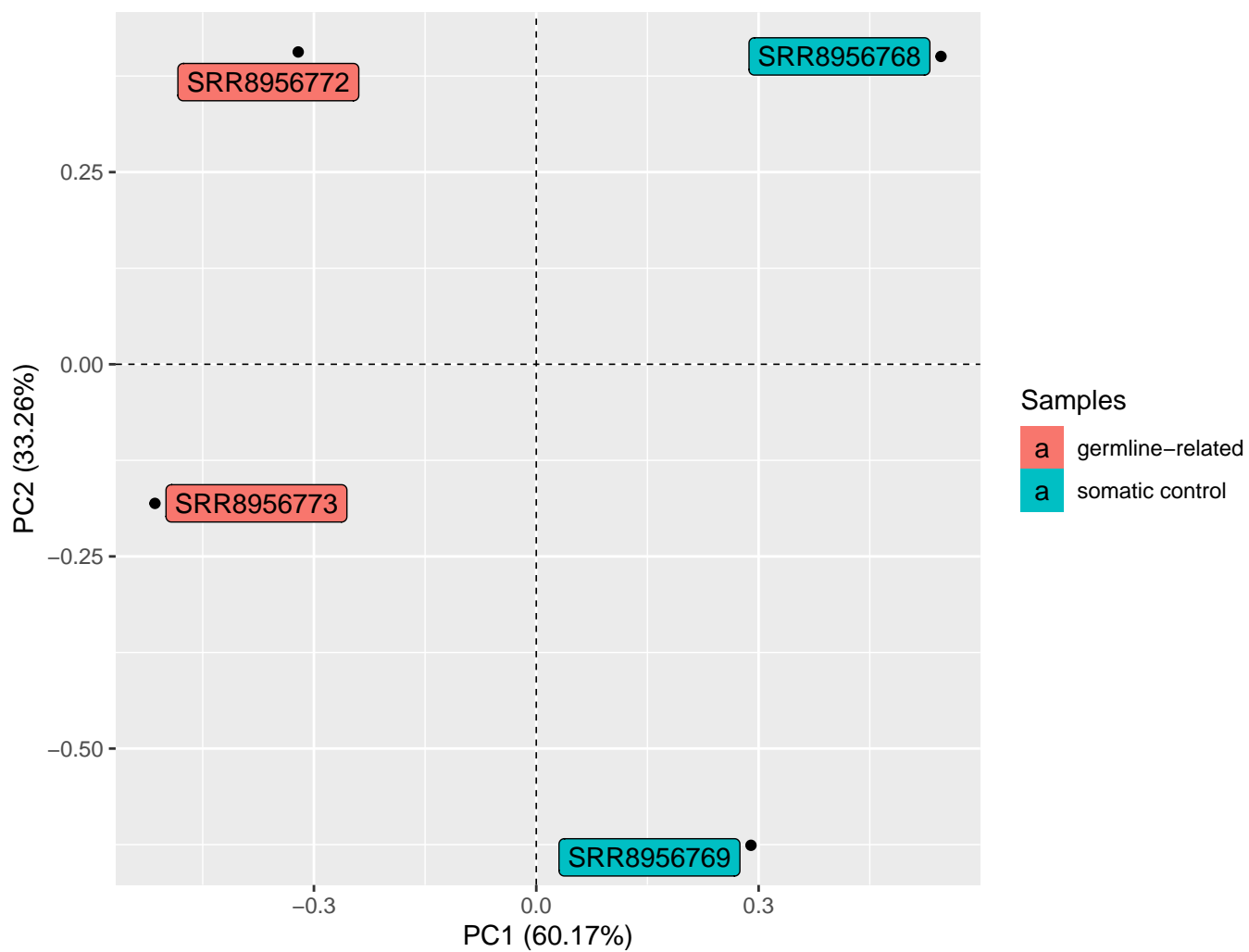

Supplement: Supplementary file 1 — Additional file 1: Zipped directory comprehending more detailed information on the species-specific transcriptomic analyses (transcriptomic statistics, read counts, fasta of upregulated transcripts, GO/IPR enrichment analyses, ratios bootstrap iterations) and extensive results of the comparative analyses (co-upregulated transcripts, co-enriched GO/IPR, comparisons of different cut-off iterations) [file 13227_2022_207_MOESM1_ESM.zip › SUPPLEMENTARY_MATERIAL/Haliotis_rufescens/Hru_PCA_logt-counts.pdf]

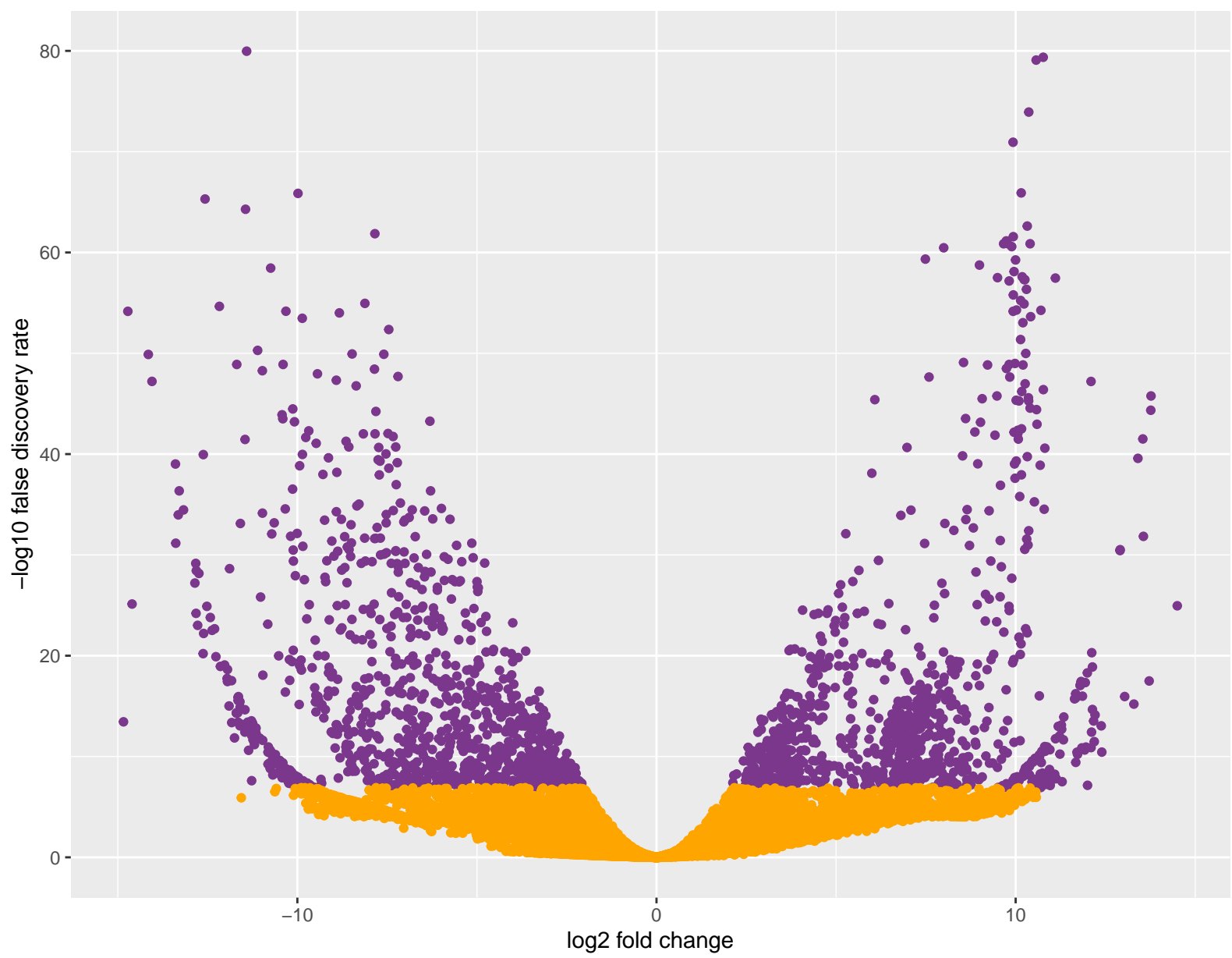

Supplement: Supplementary file 1 — Additional file 1: Zipped directory comprehending more detailed information on the species-specific transcriptomic analyses (transcriptomic statistics, read counts, fasta of upregulated transcripts, GO/IPR enrichment analyses, ratios bootstrap iterations) and extensive results of the comparative analyses (co-upregulated transcripts, co-enriched GO/IPR, comparisons of different cut-off iterations) [file 13227_2022_207_MOESM1_ESM.zip › SUPPLEMENTARY_MATERIAL/Haliotis_rufescens/Hru_Vplot_edgeR.pdf]

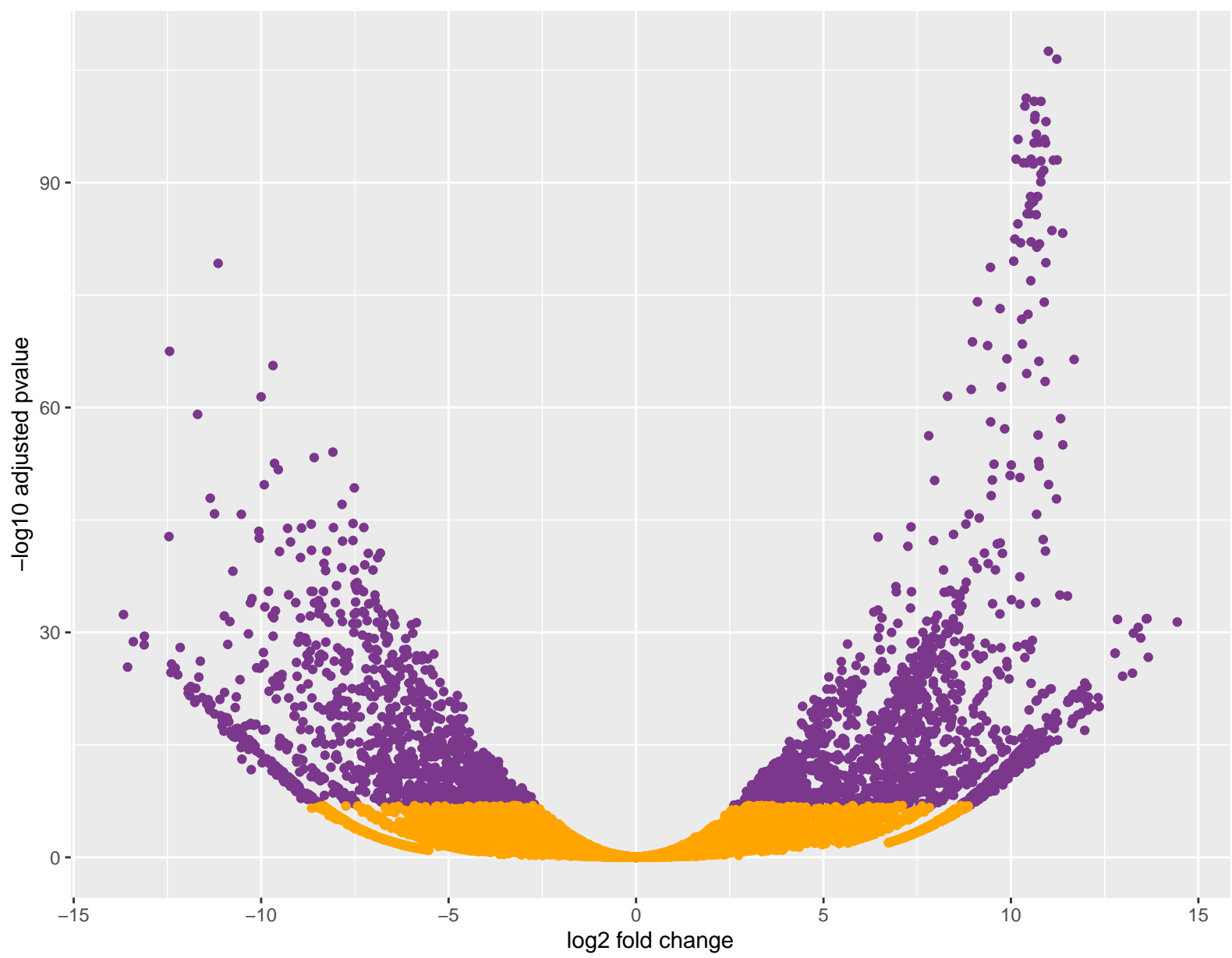

Supplement: Supplementary file 1 — Additional file 1: Zipped directory comprehending more detailed information on the species-specific transcriptomic analyses (transcriptomic statistics, read counts, fasta of upregulated transcripts, GO/IPR enrichment analyses, ratios bootstrap iterations) and extensive results of the comparative analyses (co-upregulated transcripts, co-enriched GO/IPR, comparisons of different cut-off iterations) [file 13227_2022_207_MOESM1_ESM.zip › SUPPLEMENTARY_MATERIAL/Haliotis_rufescens/Hru_Vplot_DESEq2.pdf]

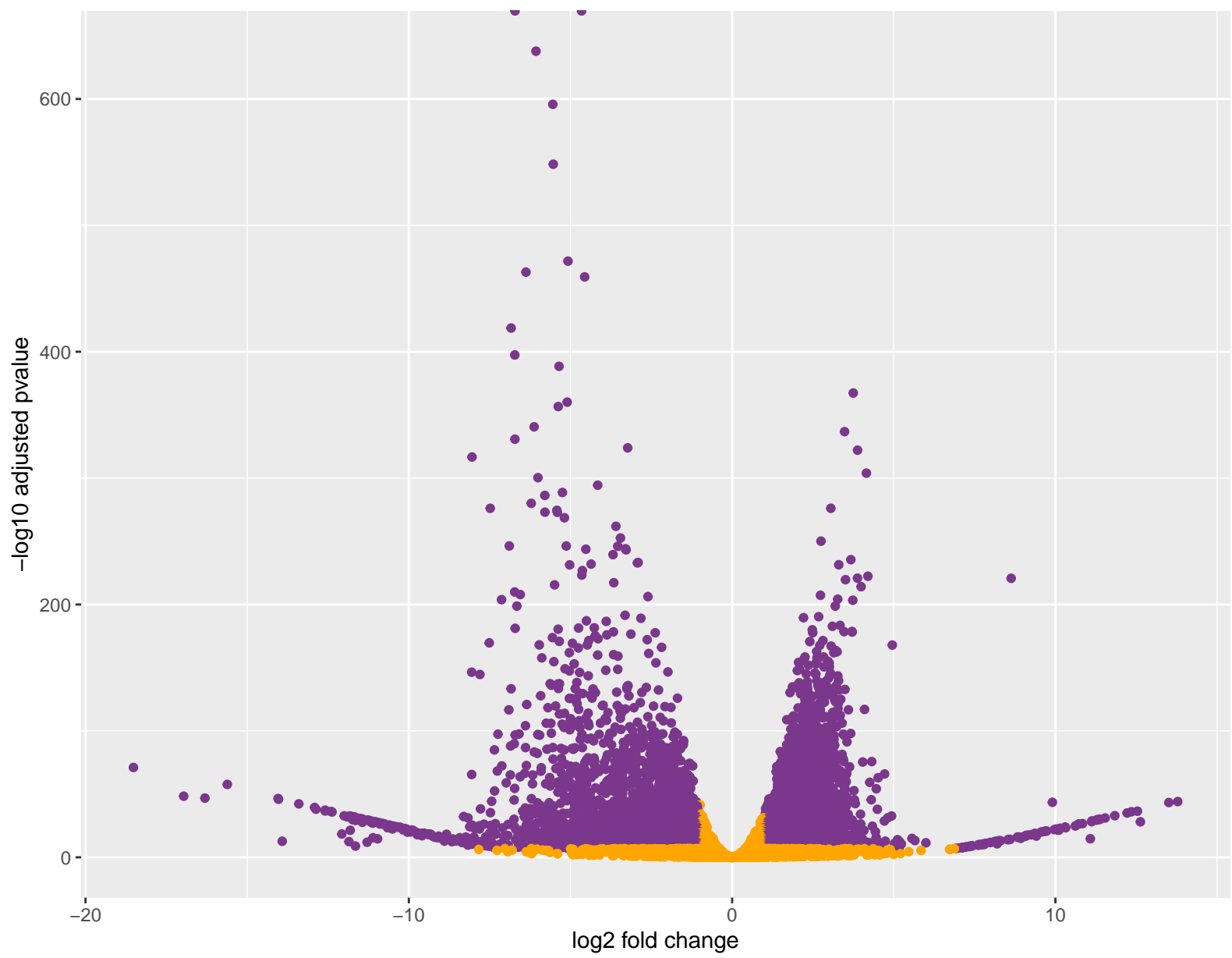

Supplement: Supplementary file 1 — Additional file 1: Zipped directory comprehending more detailed information on the species-specific transcriptomic analyses (transcriptomic statistics, read counts, fasta of upregulated transcripts, GO/IPR enrichment analyses, ratios bootstrap iterations) and extensive results of the comparative analyses (co-upregulated transcripts, co-enriched GO/IPR, comparisons of different cut-off iterations) [file 13227_2022_207_MOESM1_ESM.zip › SUPPLEMENTARY_MATERIAL/Ephydatia_fluviatilis/Efl_Vplot_DESEq2.pdf]

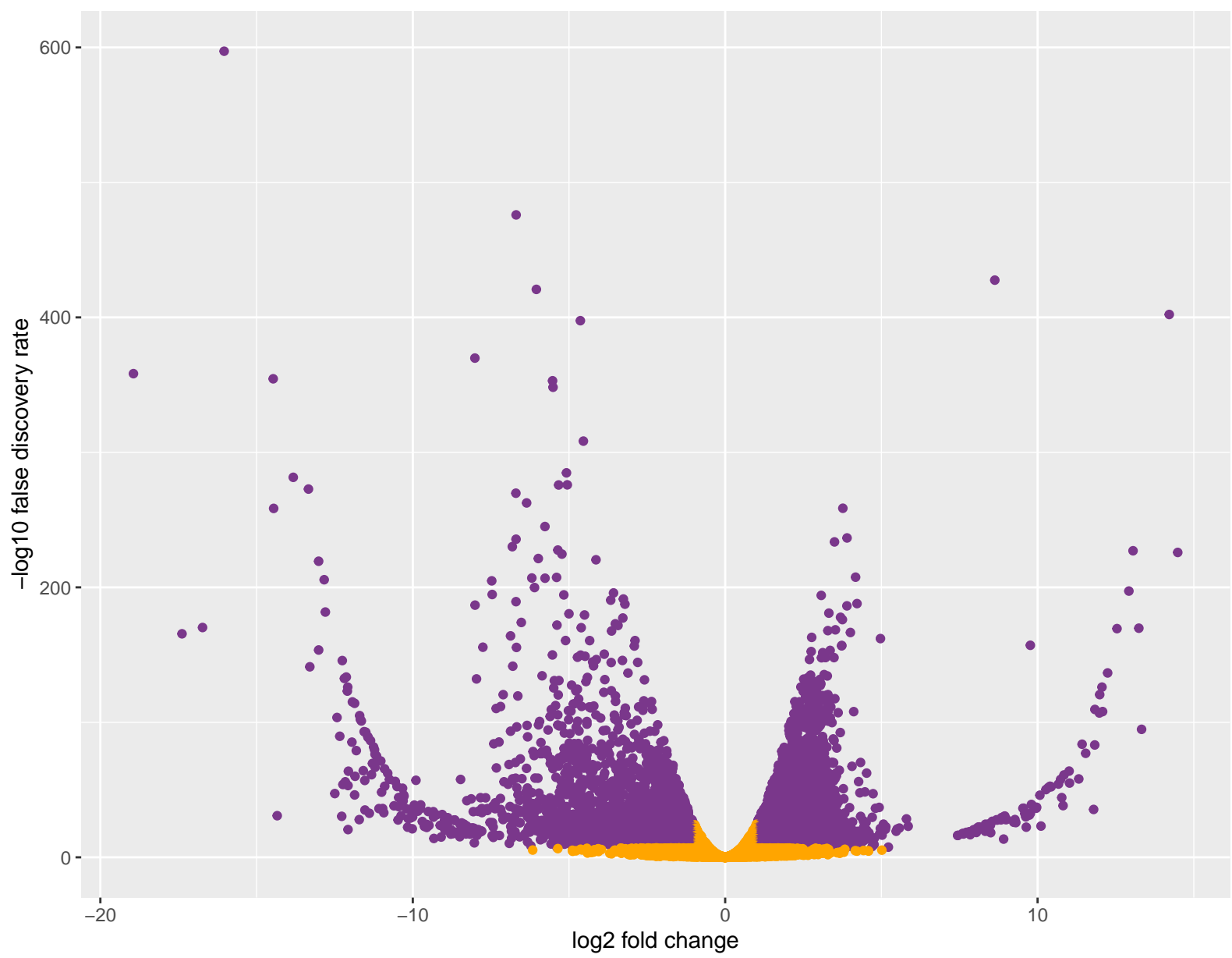

Supplement: Supplementary file 1 — Additional file 1: Zipped directory comprehending more detailed information on the species-specific transcriptomic analyses (transcriptomic statistics, read counts, fasta of upregulated transcripts, GO/IPR enrichment analyses, ratios bootstrap iterations) and extensive results of the comparative analyses (co-upregulated transcripts, co-enriched GO/IPR, comparisons of different cut-off iterations) [file 13227_2022_207_MOESM1_ESM.zip › SUPPLEMENTARY_MATERIAL/Ephydatia_fluviatilis/Efl_Vplot_edgeR.pdf]

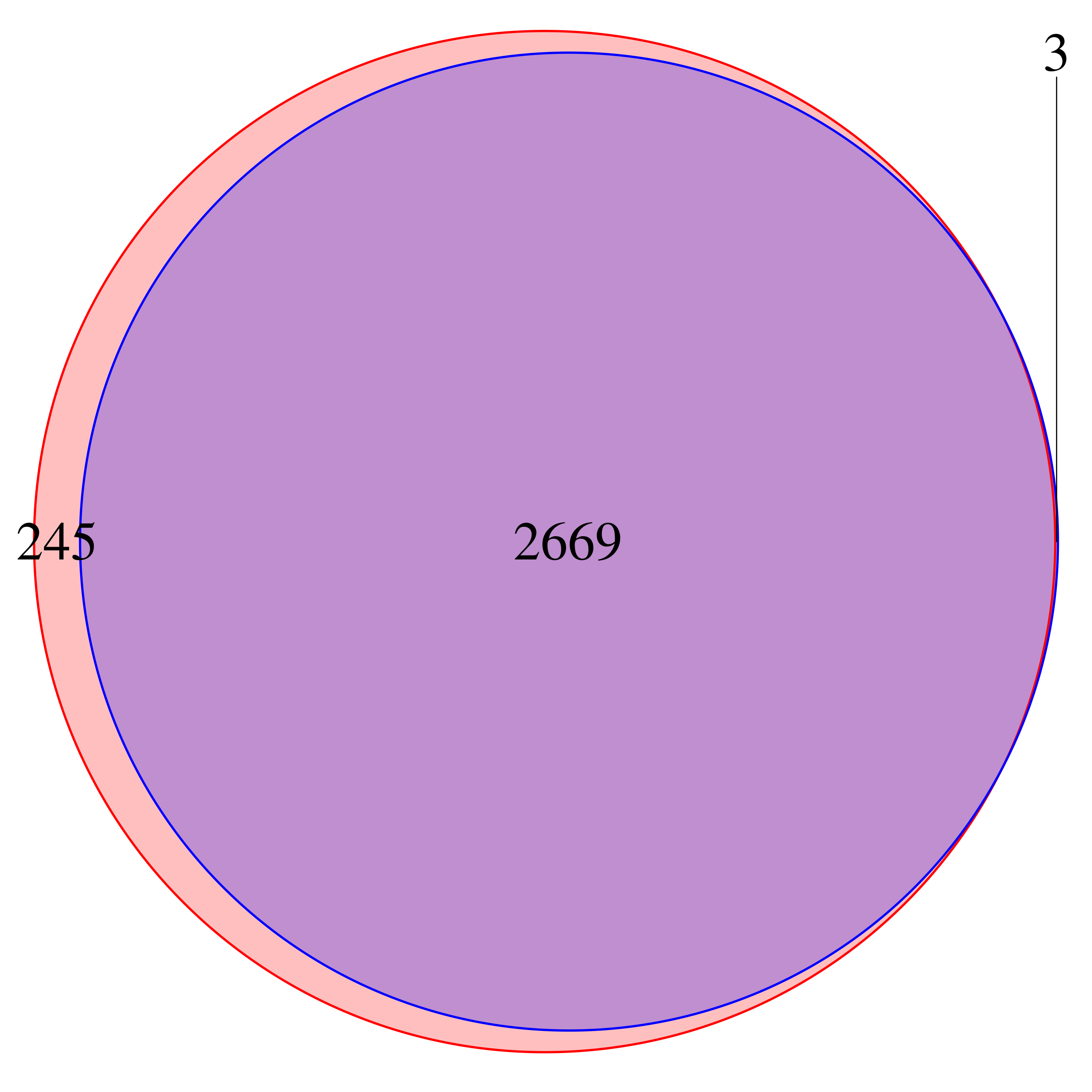

Supplement: Supplementary file 1 — Additional file 1: Zipped directory comprehending more detailed information on the species-specific transcriptomic analyses (transcriptomic statistics, read counts, fasta of upregulated transcripts, GO/IPR enrichment analyses, ratios bootstrap iterations) and extensive results of the comparative analyses (co-upregulated transcripts, co-enriched GO/IPR, comparisons of different cut-off iterations) [file 13227_2022_207_MOESM1_ESM.zip › SUPPLEMENTARY_MATERIAL/Ephydatia_fluviatilis/Efl_DESeq2-edgeR_Venn.png]

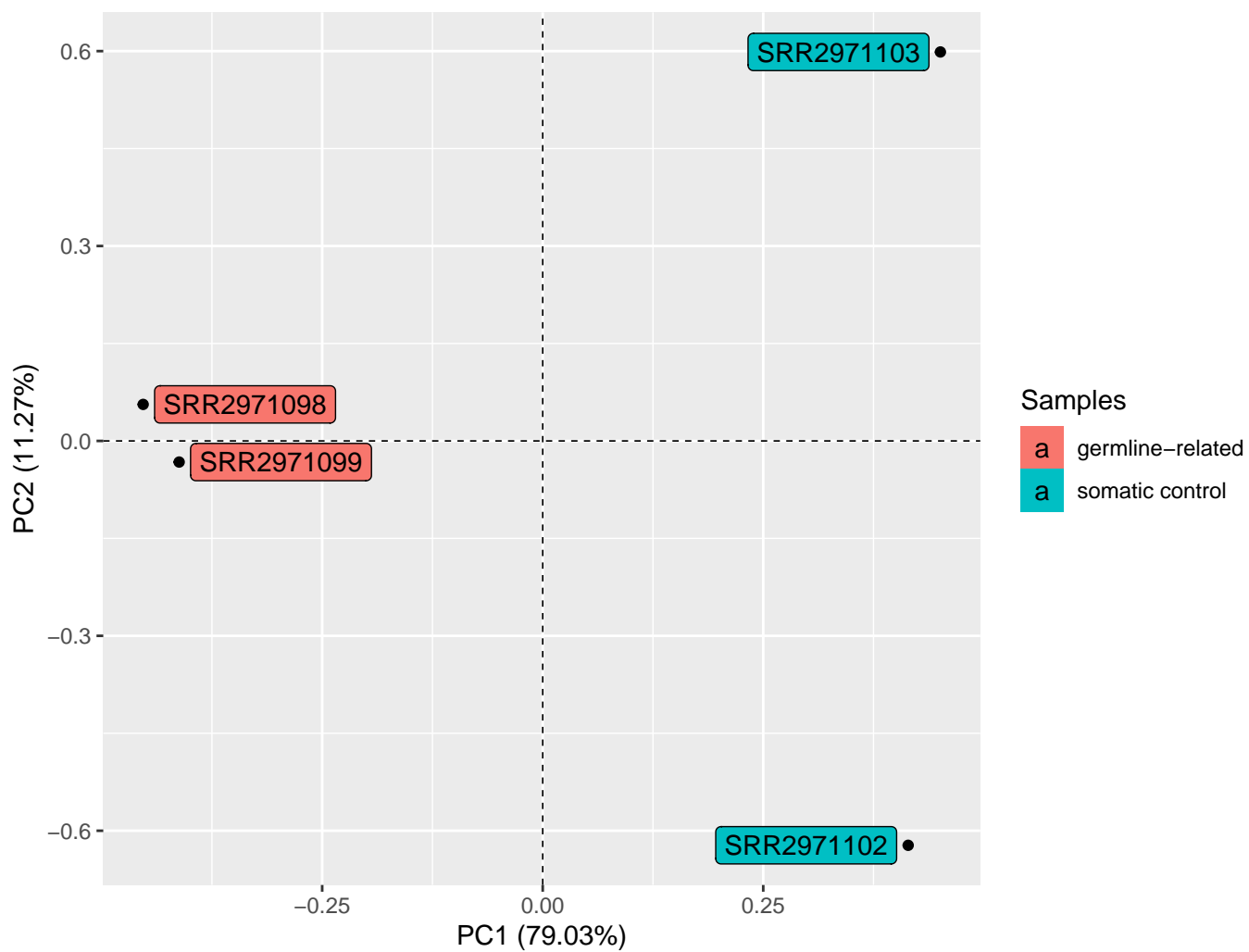

Supplement: Supplementary file 1 — Additional file 1: Zipped directory comprehending more detailed information on the species-specific transcriptomic analyses (transcriptomic statistics, read counts, fasta of upregulated transcripts, GO/IPR enrichment analyses, ratios bootstrap iterations) and extensive results of the comparative analyses (co-upregulated transcripts, co-enriched GO/IPR, comparisons of different cut-off iterations) [file 13227_2022_207_MOESM1_ESM.zip › SUPPLEMENTARY_MATERIAL/Ephydatia_fluviatilis/Efl_PCA_logt-counts.pdf]

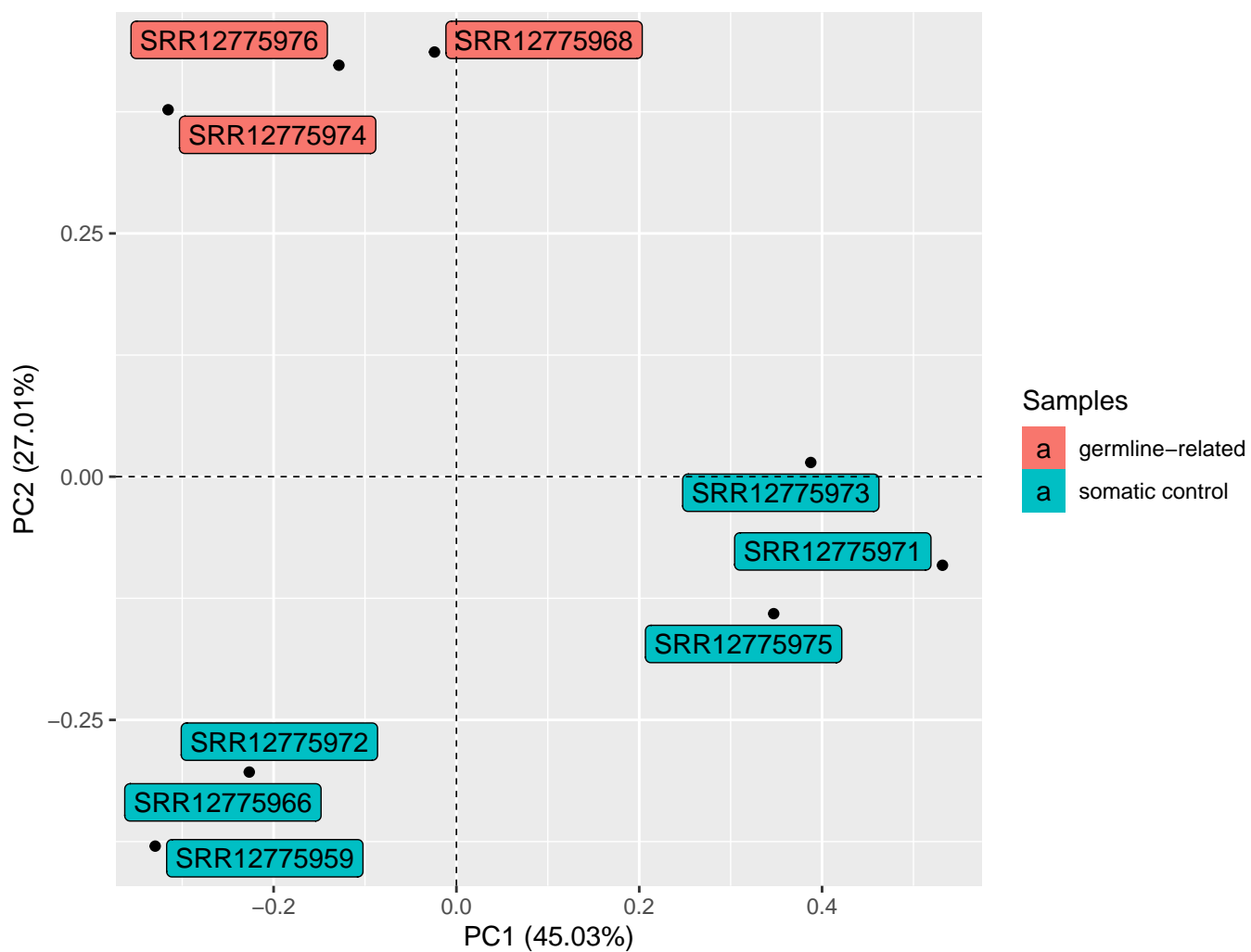

Supplement: Supplementary file 1 — Additional file 1: Zipped directory comprehending more detailed information on the species-specific transcriptomic analyses (transcriptomic statistics, read counts, fasta of upregulated transcripts, GO/IPR enrichment analyses, ratios bootstrap iterations) and extensive results of the comparative analyses (co-upregulated transcripts, co-enriched GO/IPR, comparisons of different cut-off iterations) [file 13227_2022_207_MOESM1_ESM.zip › SUPPLEMENTARY_MATERIAL/Nematostella_vectensis/Nve_PCA_logt-counts.pdf]

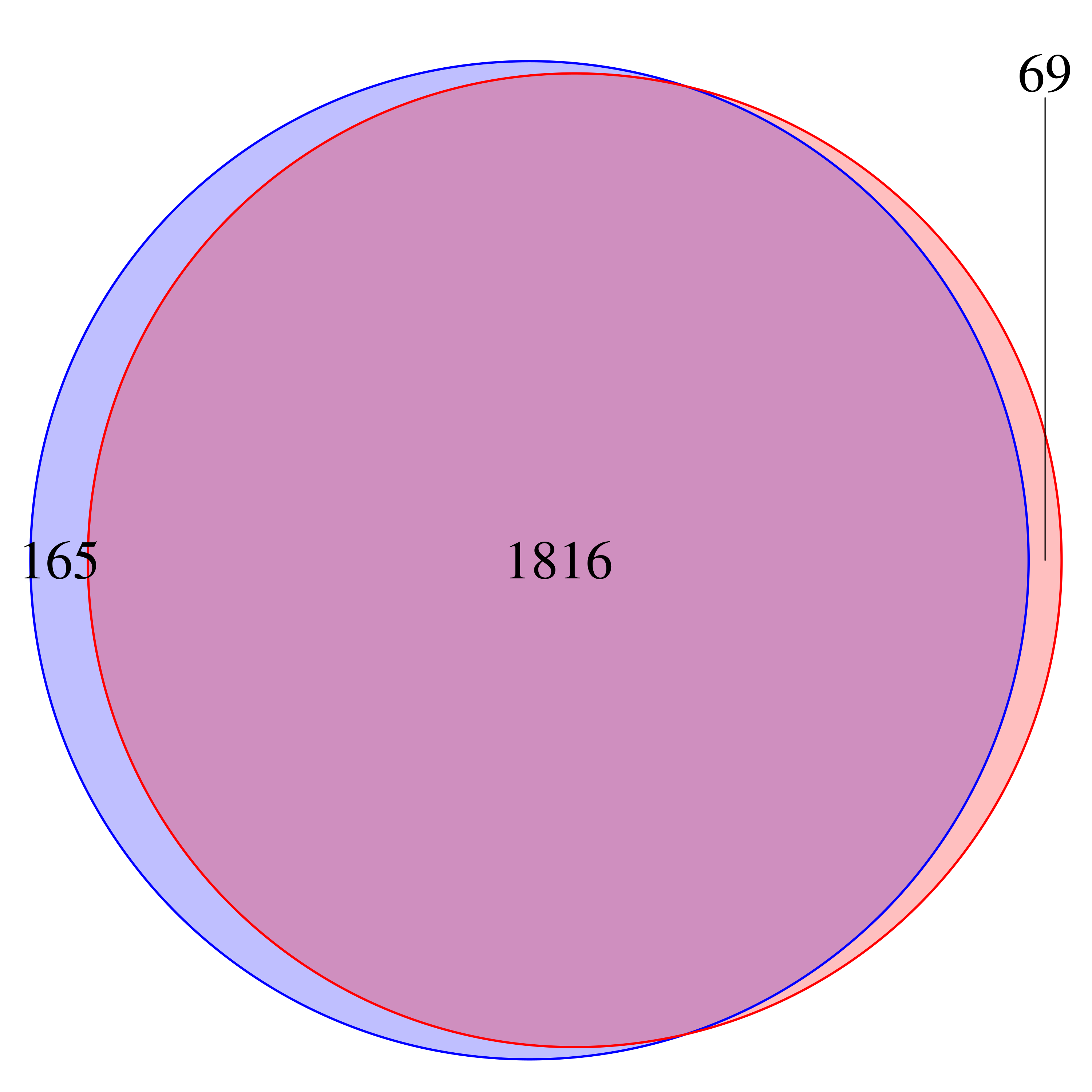

Supplement: Supplementary file 1 — Additional file 1: Zipped directory comprehending more detailed information on the species-specific transcriptomic analyses (transcriptomic statistics, read counts, fasta of upregulated transcripts, GO/IPR enrichment analyses, ratios bootstrap iterations) and extensive results of the comparative analyses (co-upregulated transcripts, co-enriched GO/IPR, comparisons of different cut-off iterations) [file 13227_2022_207_MOESM1_ESM.zip › SUPPLEMENTARY_MATERIAL/Nematostella_vectensis/Nve_DESeq2-edgeR_Venn.png]

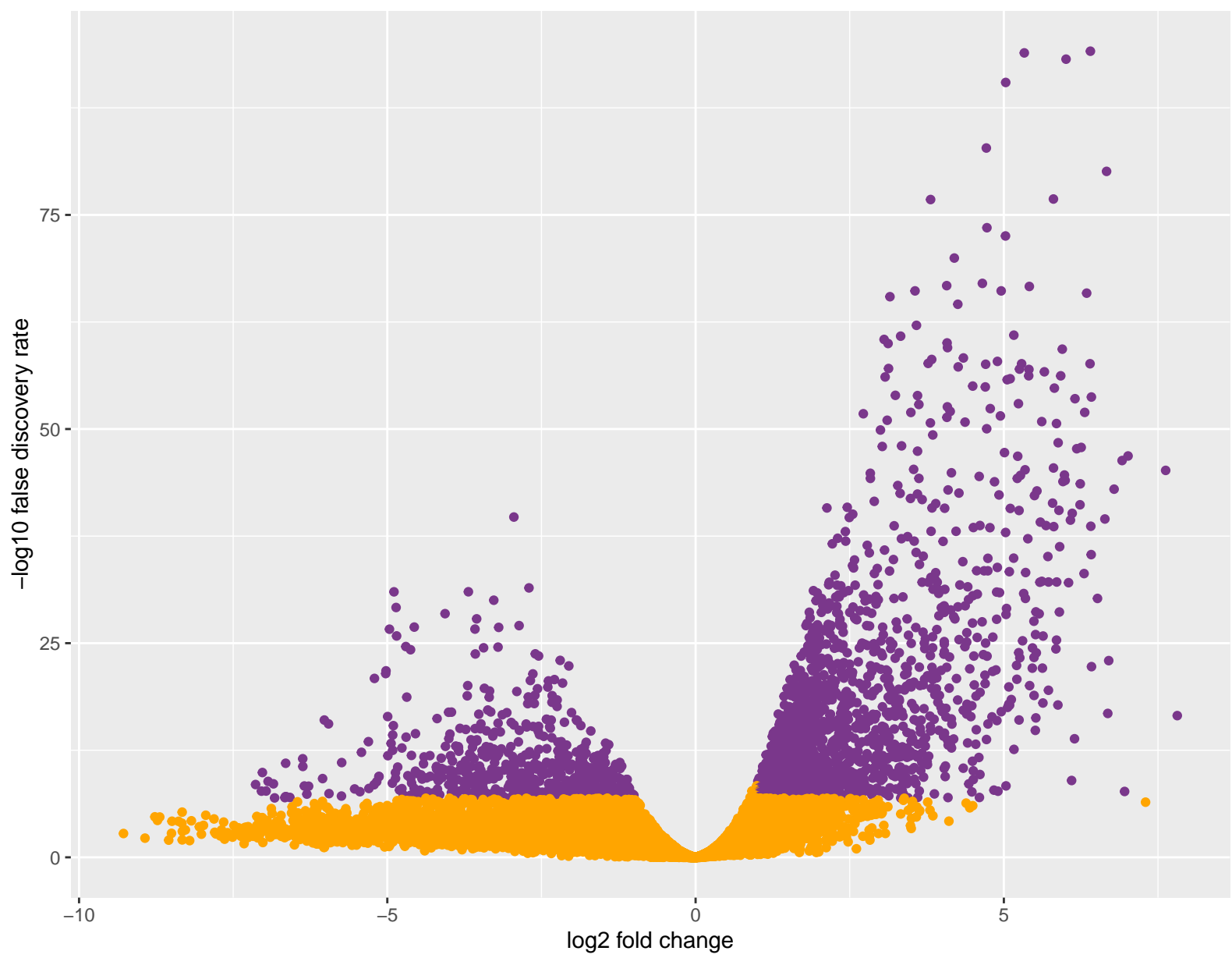

Supplement: Supplementary file 1 — Additional file 1: Zipped directory comprehending more detailed information on the species-specific transcriptomic analyses (transcriptomic statistics, read counts, fasta of upregulated transcripts, GO/IPR enrichment analyses, ratios bootstrap iterations) and extensive results of the comparative analyses (co-upregulated transcripts, co-enriched GO/IPR, comparisons of different cut-off iterations) [file 13227_2022_207_MOESM1_ESM.zip › SUPPLEMENTARY_MATERIAL/Nematostella_vectensis/Nve_Vplot_edgeR.pdf]

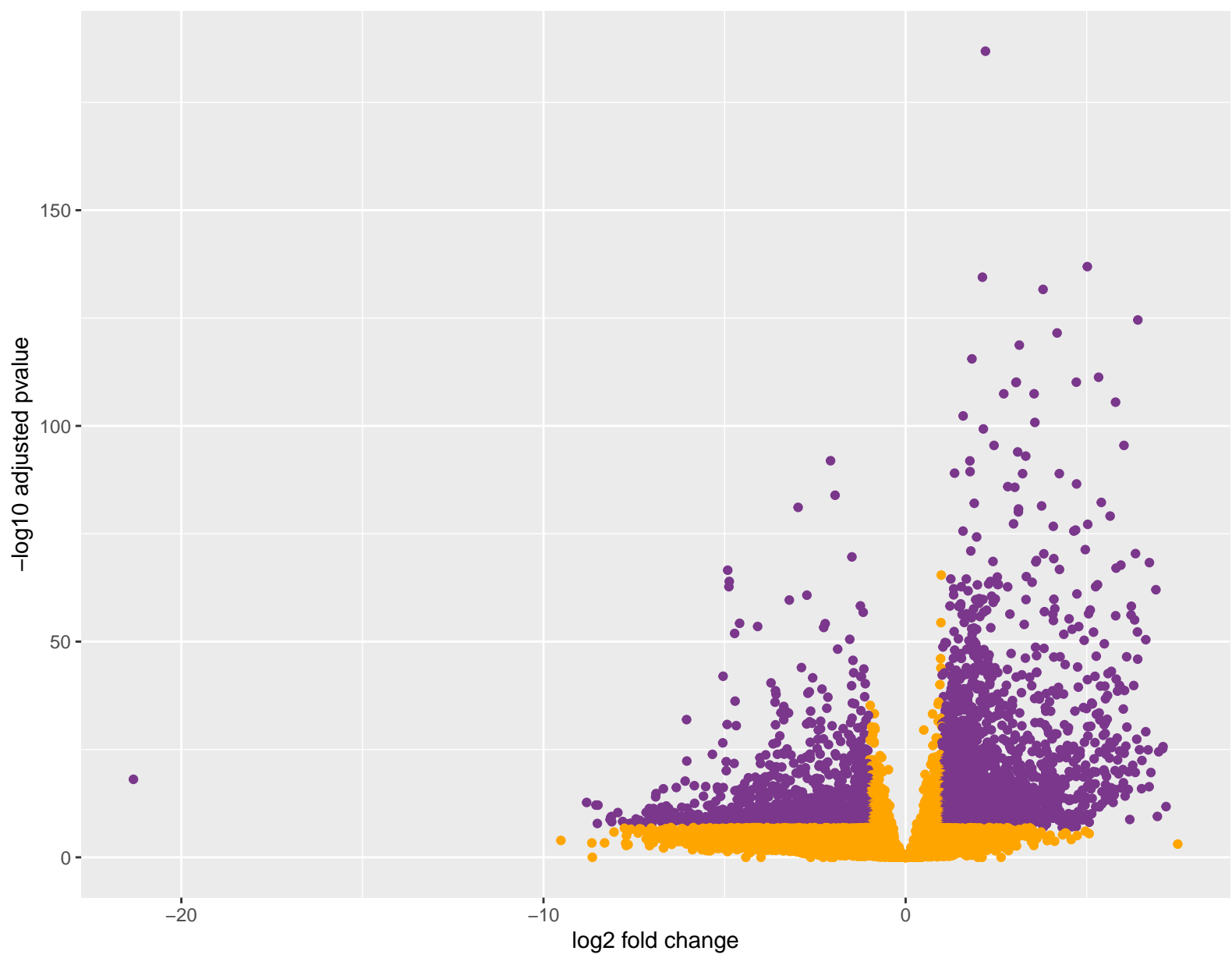

Supplement: Supplementary file 1 — Additional file 1: Zipped directory comprehending more detailed information on the species-specific transcriptomic analyses (transcriptomic statistics, read counts, fasta of upregulated transcripts, GO/IPR enrichment analyses, ratios bootstrap iterations) and extensive results of the comparative analyses (co-upregulated transcripts, co-enriched GO/IPR, comparisons of different cut-off iterations) [file 13227_2022_207_MOESM1_ESM.zip › SUPPLEMENTARY_MATERIAL/Nematostella_vectensis/Nve_Vplot_DESEq2.pdf]

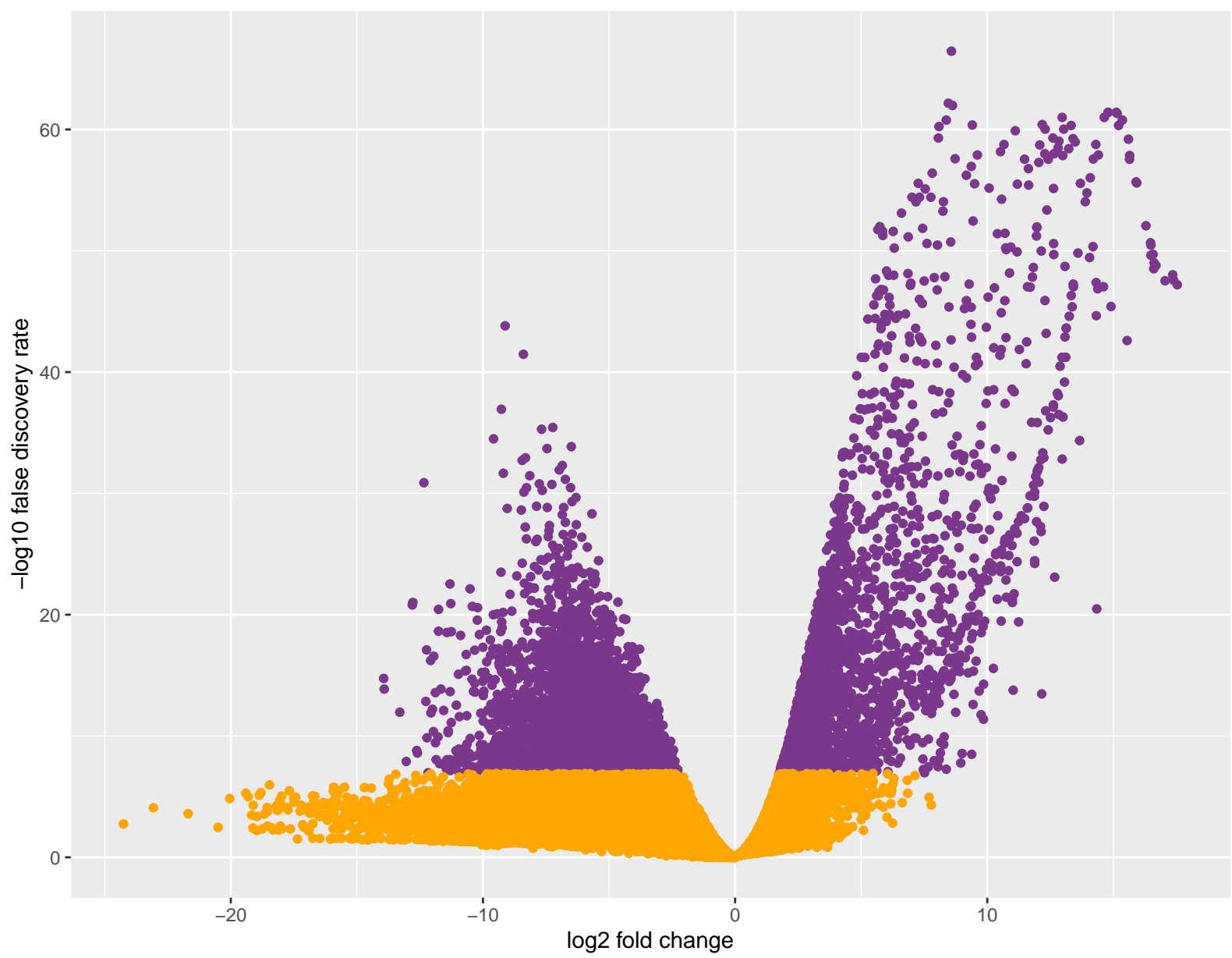

Supplement: Supplementary file 1 — Additional file 1: Zipped directory comprehending more detailed information on the species-specific transcriptomic analyses (transcriptomic statistics, read counts, fasta of upregulated transcripts, GO/IPR enrichment analyses, ratios bootstrap iterations) and extensive results of the comparative analyses (co-upregulated transcripts, co-enriched GO/IPR, comparisons of different cut-off iterations) [file 13227_2022_207_MOESM1_ESM.zip › SUPPLEMENTARY_MATERIAL/Xenopus_tropicalis/Xtr_Vplot_edgeR.pdf]

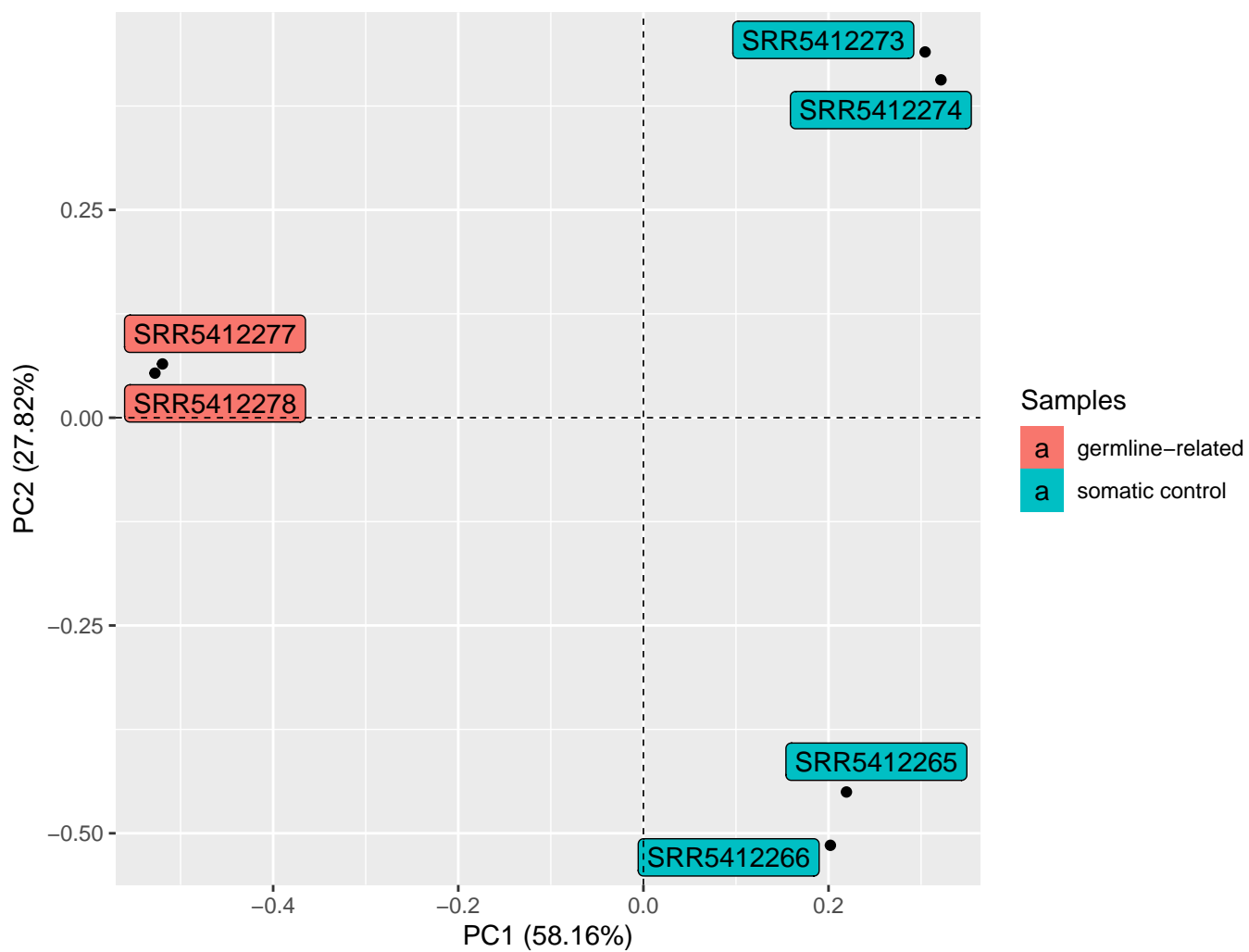

Supplement: Supplementary file 1 — Additional file 1: Zipped directory comprehending more detailed information on the species-specific transcriptomic analyses (transcriptomic statistics, read counts, fasta of upregulated transcripts, GO/IPR enrichment analyses, ratios bootstrap iterations) and extensive results of the comparative analyses (co-upregulated transcripts, co-enriched GO/IPR, comparisons of different cut-off iterations) [file 13227_2022_207_MOESM1_ESM.zip › SUPPLEMENTARY_MATERIAL/Xenopus_tropicalis/Xtr_PCA_logt-counts.pdf]

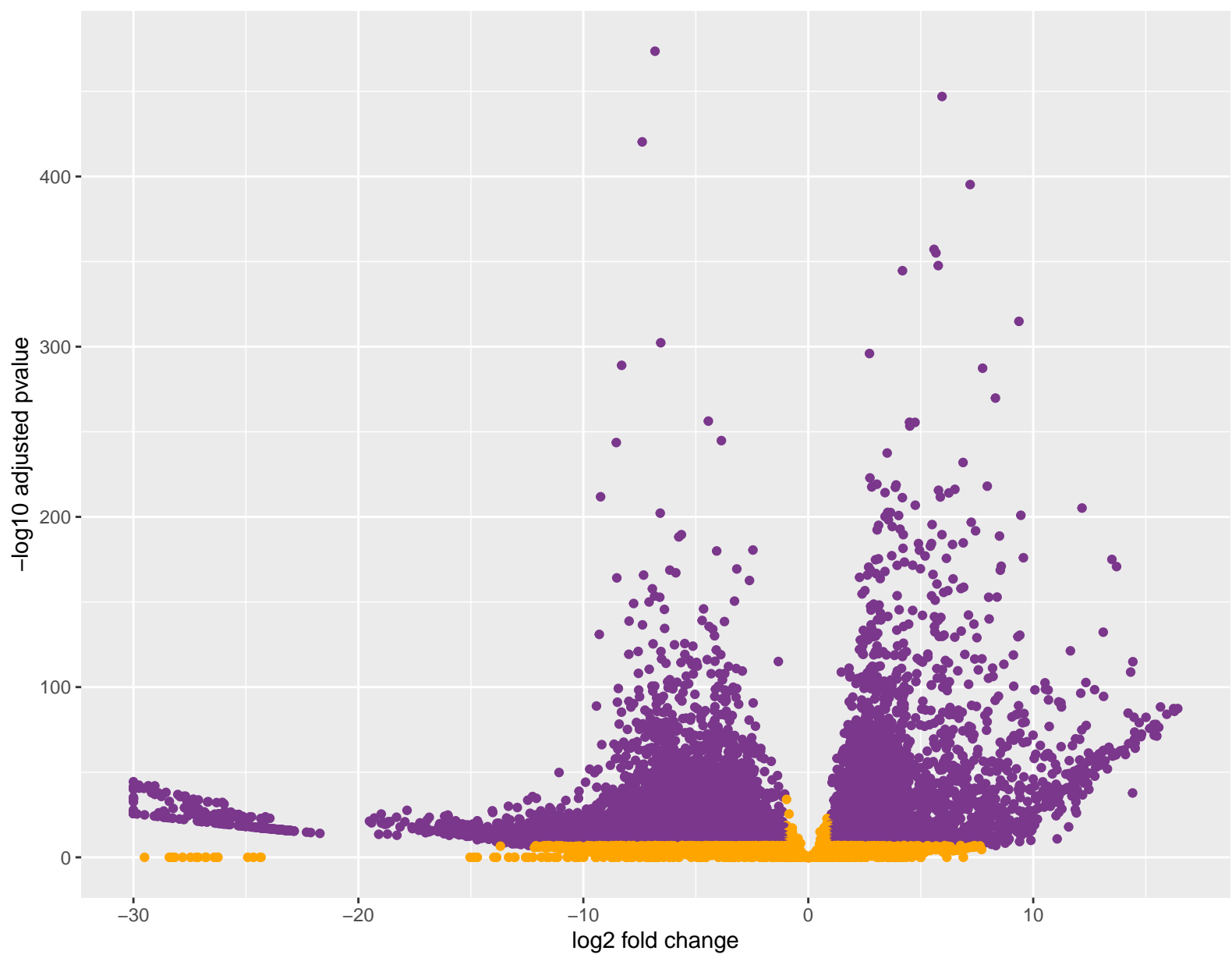

Supplement: Supplementary file 1 — Additional file 1: Zipped directory comprehending more detailed information on the species-specific transcriptomic analyses (transcriptomic statistics, read counts, fasta of upregulated transcripts, GO/IPR enrichment analyses, ratios bootstrap iterations) and extensive results of the comparative analyses (co-upregulated transcripts, co-enriched GO/IPR, comparisons of different cut-off iterations) [file 13227_2022_207_MOESM1_ESM.zip › SUPPLEMENTARY_MATERIAL/Xenopus_tropicalis/Xtr_Vplot_DESEq2.pdf]

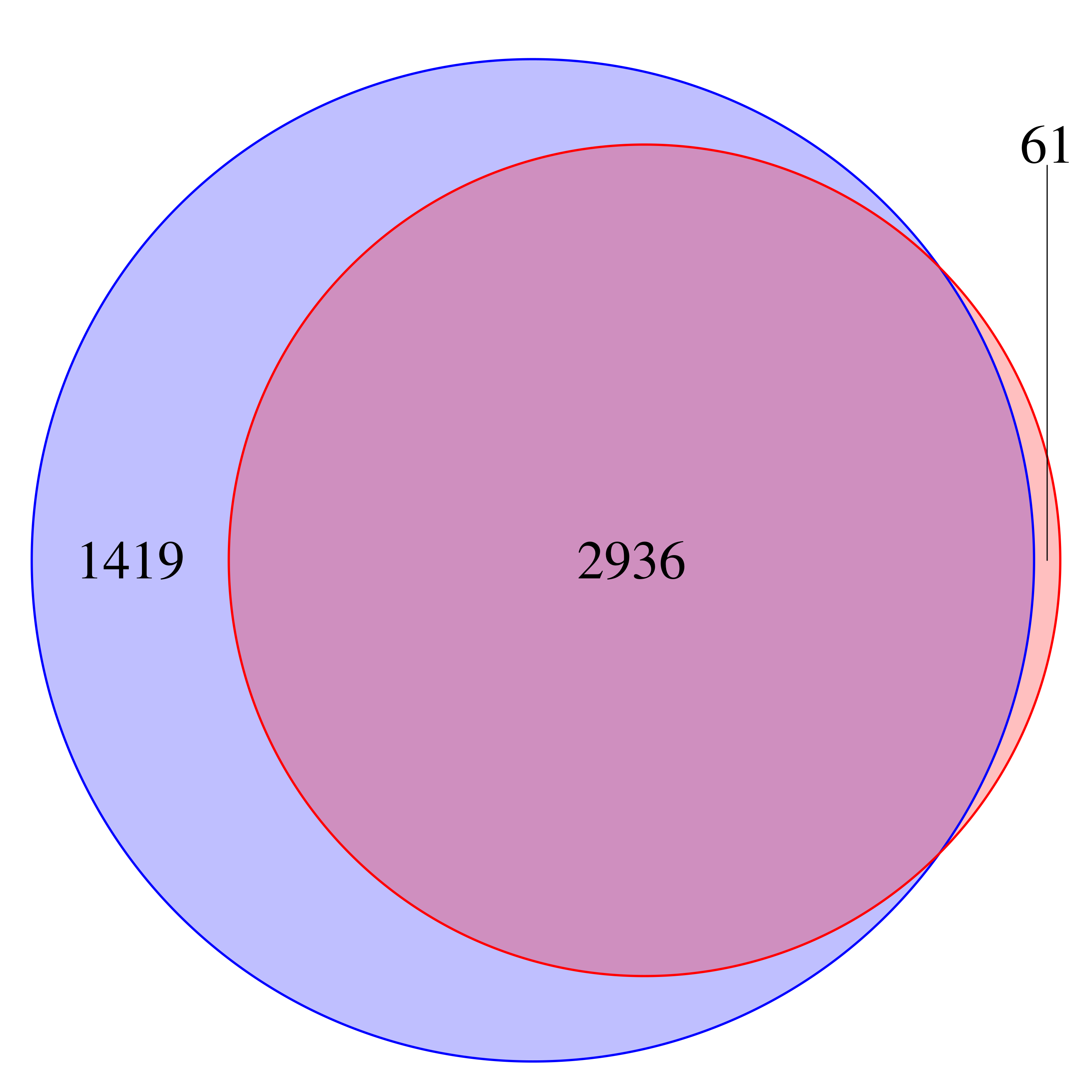

Supplement: Supplementary file 1 — Additional file 1: Zipped directory comprehending more detailed information on the species-specific transcriptomic analyses (transcriptomic statistics, read counts, fasta of upregulated transcripts, GO/IPR enrichment analyses, ratios bootstrap iterations) and extensive results of the comparative analyses (co-upregulated transcripts, co-enriched GO/IPR, comparisons of different cut-off iterations) [file 13227_2022_207_MOESM1_ESM.zip › SUPPLEMENTARY_MATERIAL/Xenopus_tropicalis/Xtr_DESeq2-edgeR_Venn.png]

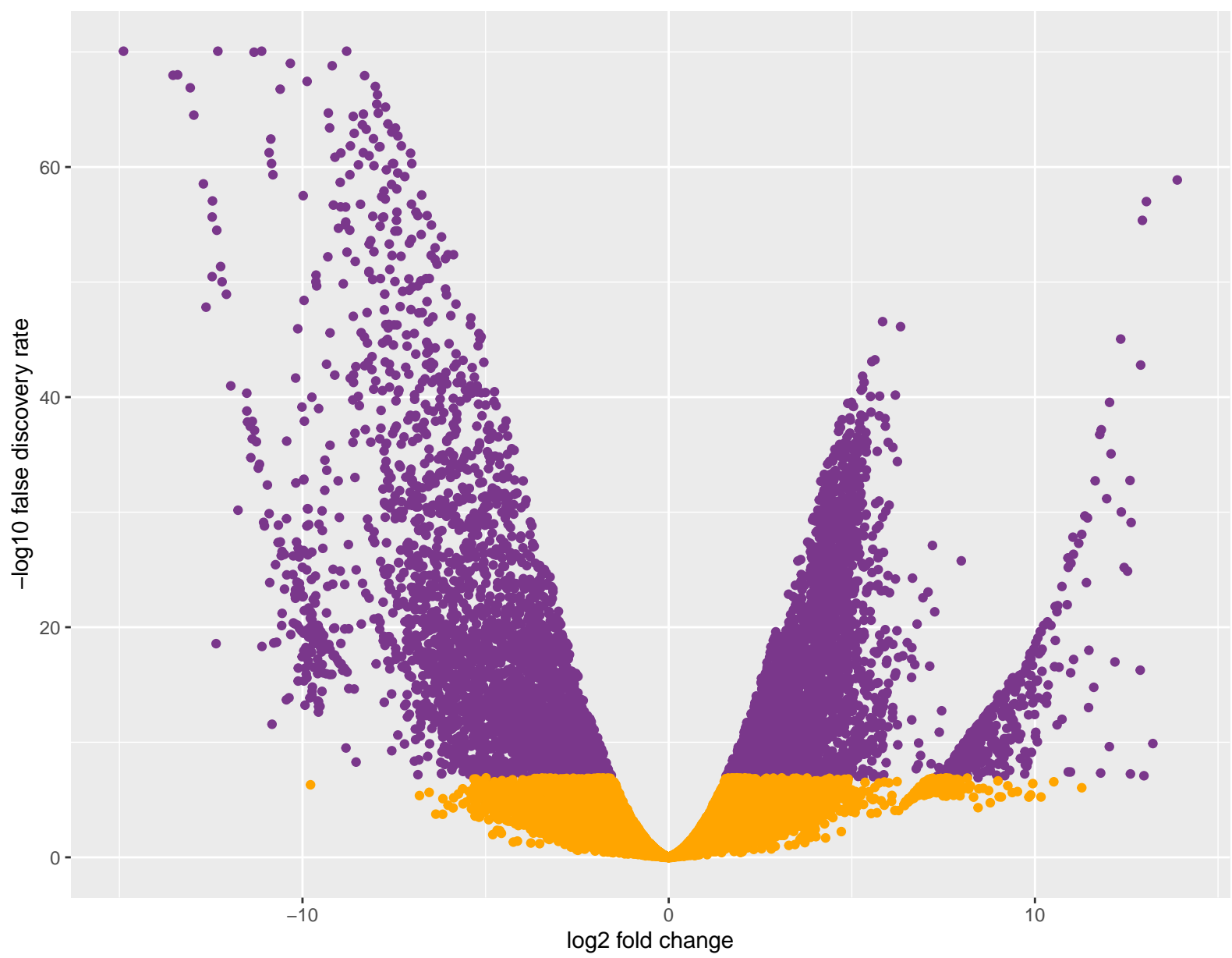

Supplement: Supplementary file 1 — Additional file 1: Zipped directory comprehending more detailed information on the species-specific transcriptomic analyses (transcriptomic statistics, read counts, fasta of upregulated transcripts, GO/IPR enrichment analyses, ratios bootstrap iterations) and extensive results of the comparative analyses (co-upregulated transcripts, co-enriched GO/IPR, comparisons of different cut-off iterations) [file 13227_2022_207_MOESM1_ESM.zip › SUPPLEMENTARY_MATERIAL/Danio_rerio/Dre_Vplot_edgeR.pdf]

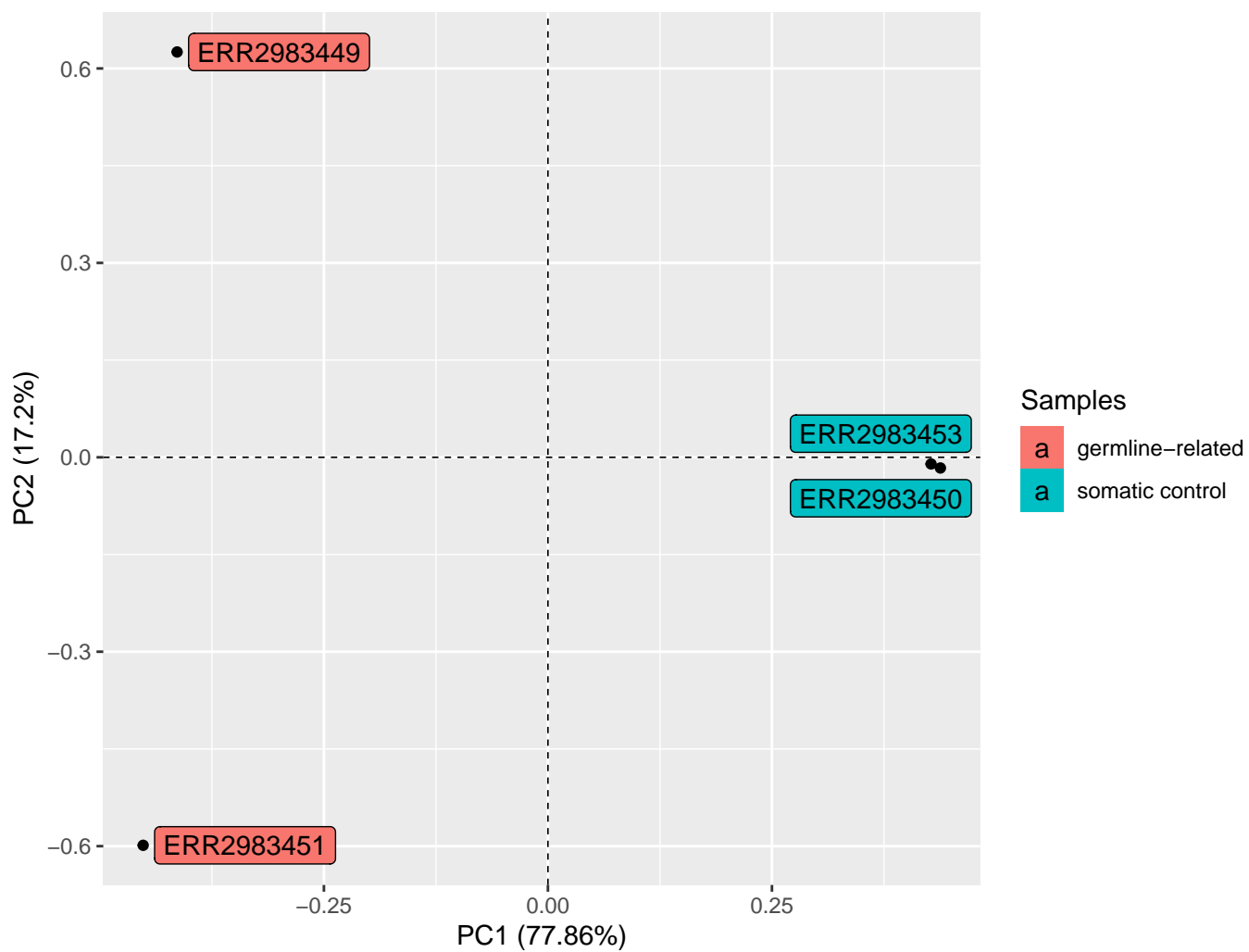

Supplement: Supplementary file 1 — Additional file 1: Zipped directory comprehending more detailed information on the species-specific transcriptomic analyses (transcriptomic statistics, read counts, fasta of upregulated transcripts, GO/IPR enrichment analyses, ratios bootstrap iterations) and extensive results of the comparative analyses (co-upregulated transcripts, co-enriched GO/IPR, comparisons of different cut-off iterations) [file 13227_2022_207_MOESM1_ESM.zip › SUPPLEMENTARY_MATERIAL/Danio_rerio/Dre_PCA_logt-counts.pdf]

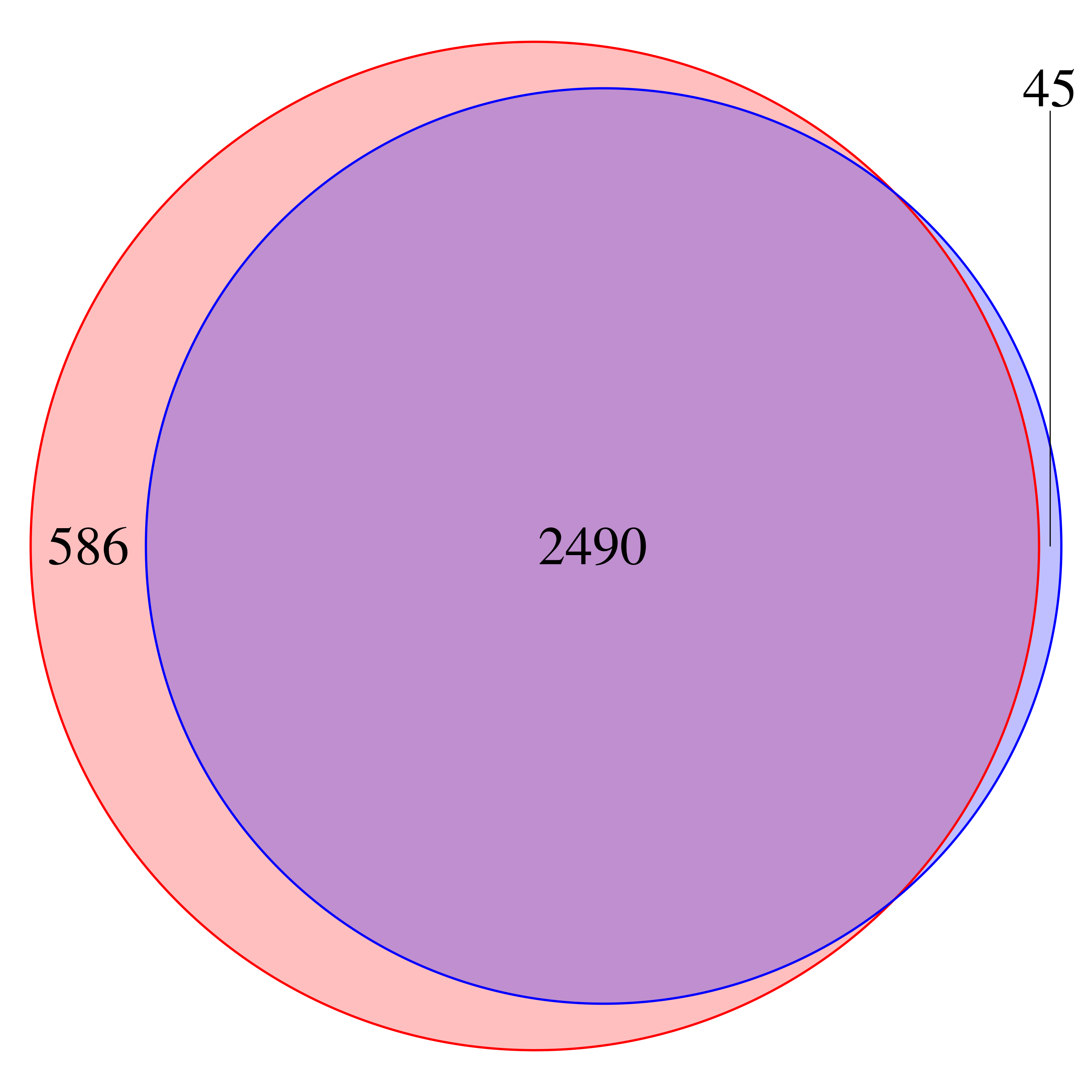

Supplement: Supplementary file 1 — Additional file 1: Zipped directory comprehending more detailed information on the species-specific transcriptomic analyses (transcriptomic statistics, read counts, fasta of upregulated transcripts, GO/IPR enrichment analyses, ratios bootstrap iterations) and extensive results of the comparative analyses (co-upregulated transcripts, co-enriched GO/IPR, comparisons of different cut-off iterations) [file 13227_2022_207_MOESM1_ESM.zip › SUPPLEMENTARY_MATERIAL/Danio_rerio/Dre_DESeq2-edgeR_Venn.png]

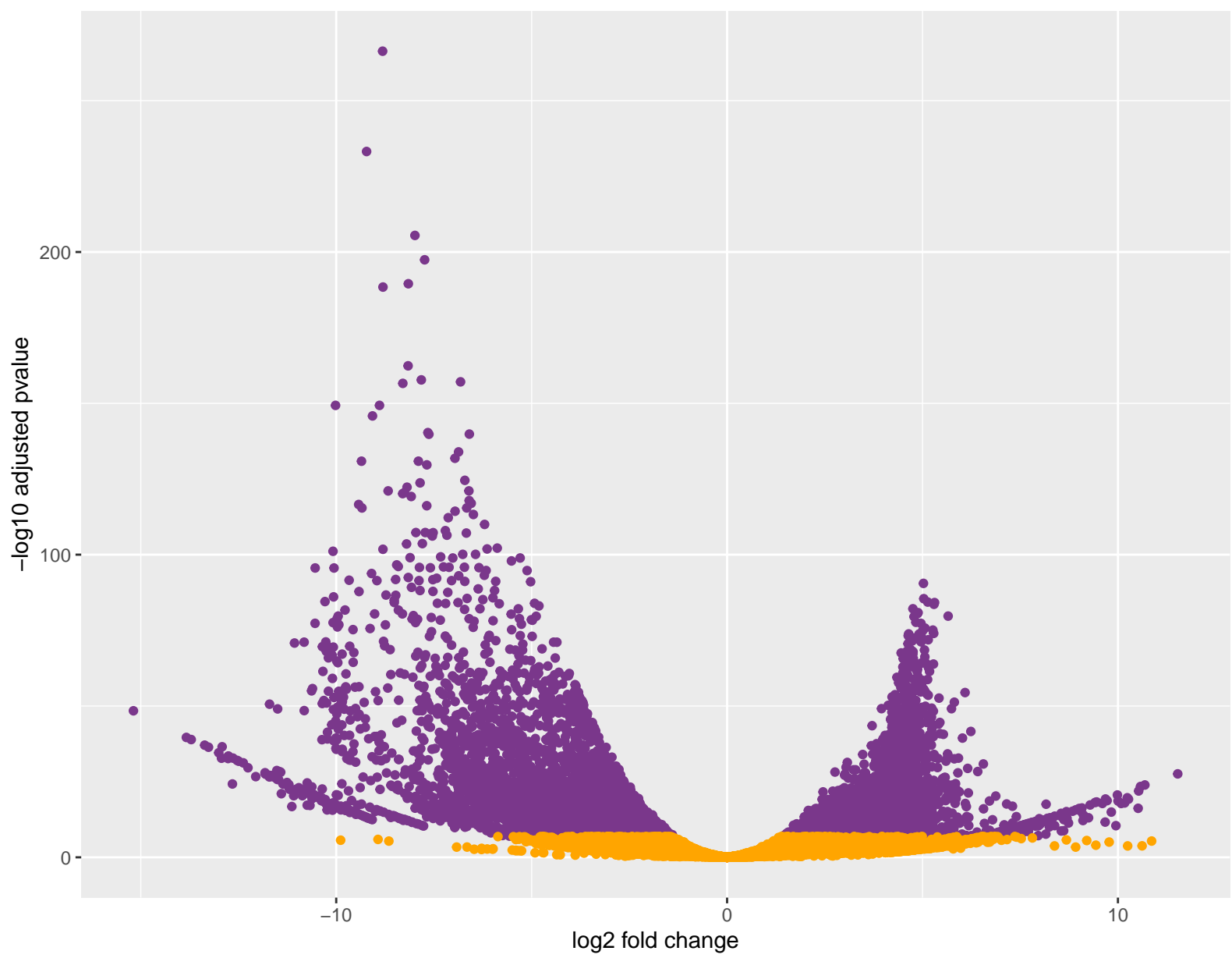

Supplement: Supplementary file 1 — Additional file 1: Zipped directory comprehending more detailed information on the species-specific transcriptomic analyses (transcriptomic statistics, read counts, fasta of upregulated transcripts, GO/IPR enrichment analyses, ratios bootstrap iterations) and extensive results of the comparative analyses (co-upregulated transcripts, co-enriched GO/IPR, comparisons of different cut-off iterations) [file 13227_2022_207_MOESM1_ESM.zip › SUPPLEMENTARY_MATERIAL/Danio_rerio/Dre_Vplot_DESEq2.pdf]

# 1000 random sets of genes

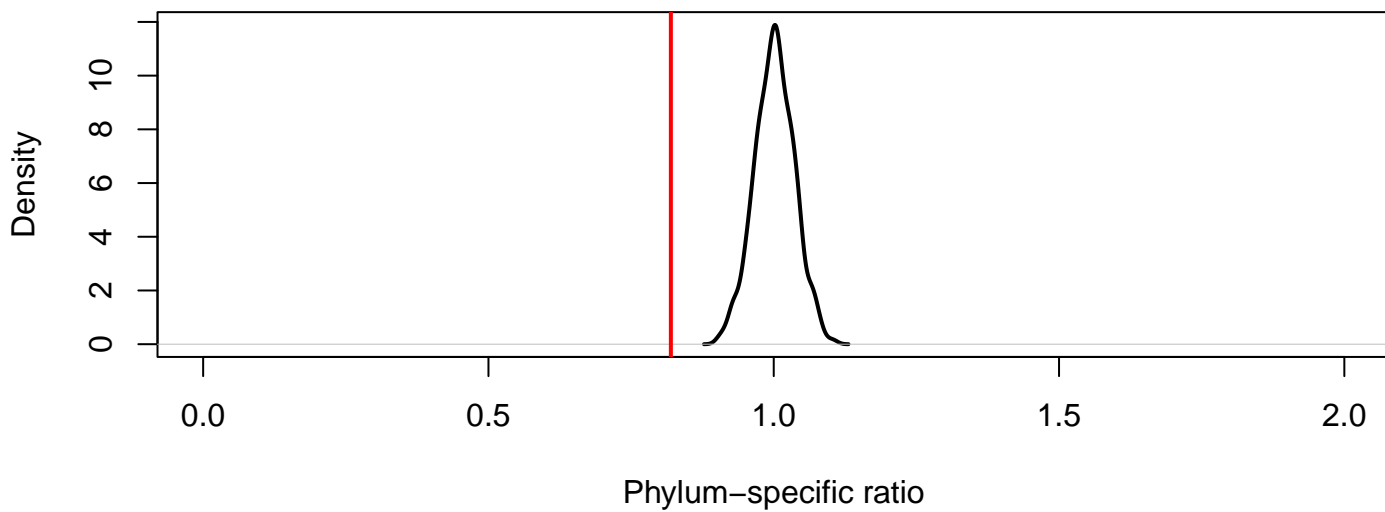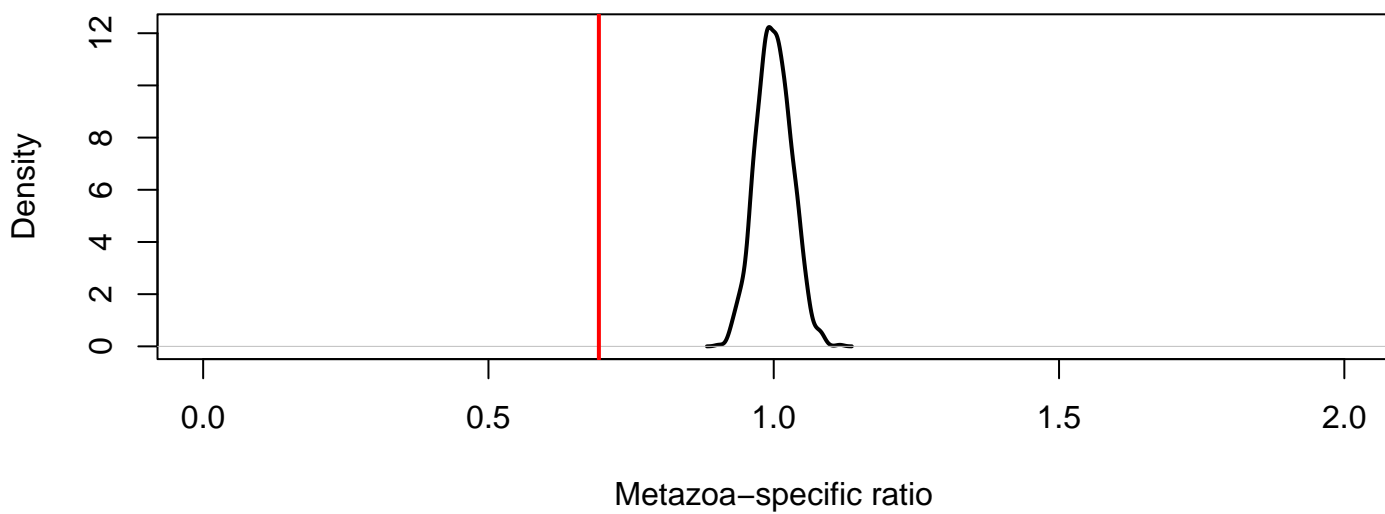

Supplement: Supplementary file 1 — Additional file 1: Zipped directory comprehending more detailed information on the species-specific transcriptomic analyses (transcriptomic statistics, read counts, fasta of upregulated transcripts, GO/IPR enrichment analyses, ratios bootstrap iterations) and extensive results of the comparative analyses (co-upregulated transcripts, co-enriched GO/IPR, comparisons of different cut-off iterations) [file 13227_2022_207_MOESM1_ESM.zip › SUPPLEMENTARY_MATERIAL/Schmidtea_mediterranea/Sme_1000sets_ratios.pdf]

# 1000 random sets of genes

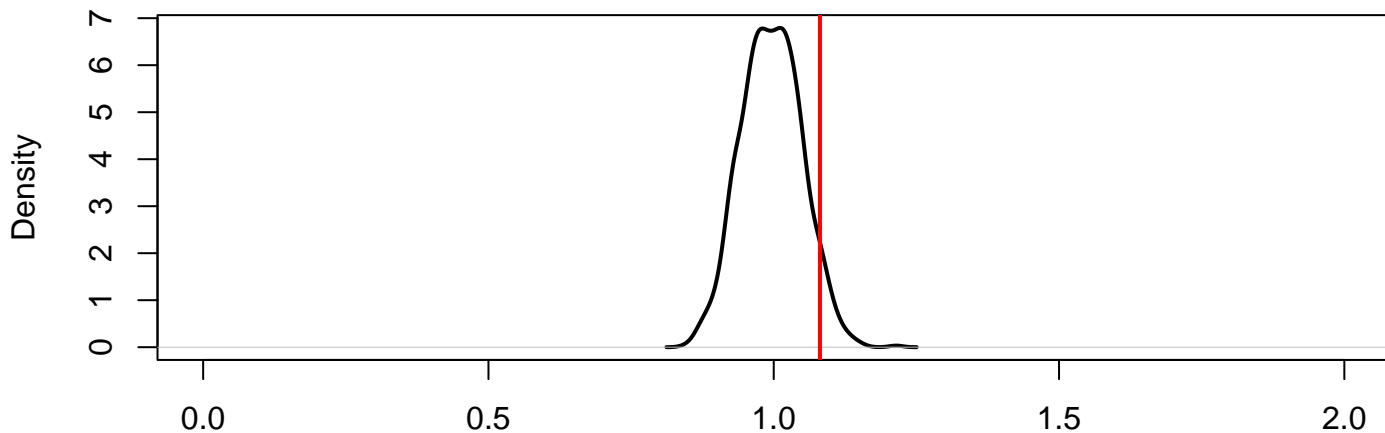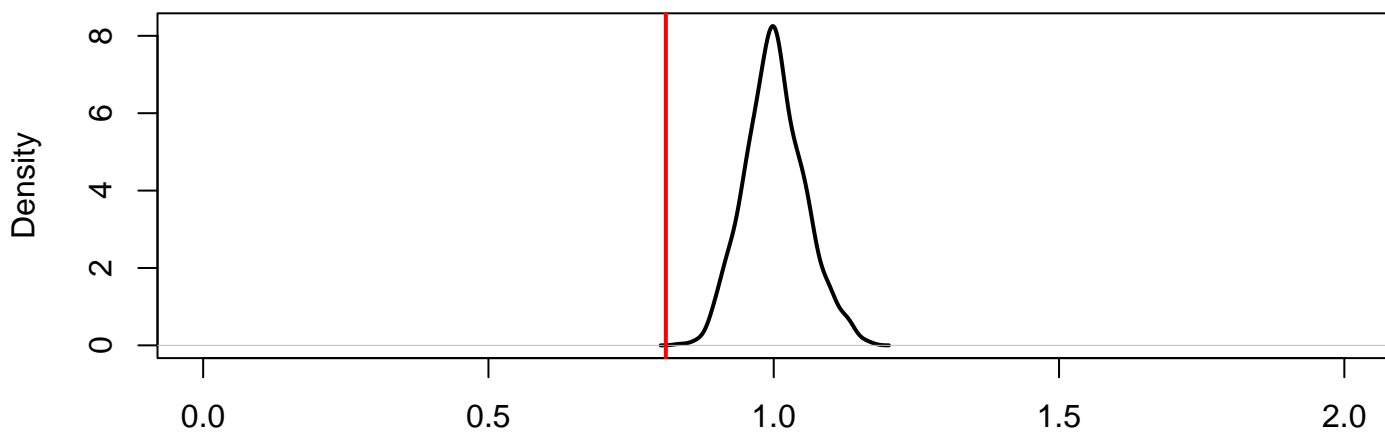

Metazoa-specific ratio

Supplement: Supplementary file 1 — Additional file 1: Zipped directory comprehending more detailed information on the species-specific transcriptomic analyses (transcriptomic statistics, read counts, fasta of upregulated transcripts, GO/IPR enrichment analyses, ratios bootstrap iterations) and extensive results of the comparative analyses (co-upregulated transcripts, co-enriched GO/IPR, comparisons of different cut-off iterations) [file 13227_2022_207_MOESM1_ESM.zip › SUPPLEMENTARY_MATERIAL/Drosophila_melanogaster/Dme_1000sets_ratios.pdf]

# 1000 random sets of genes

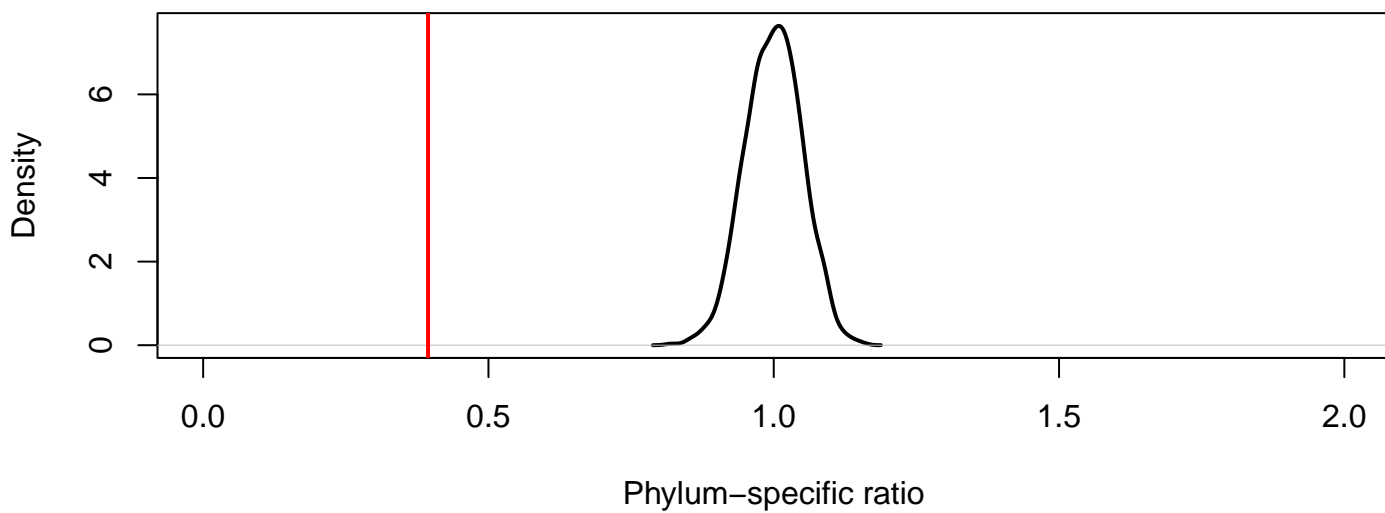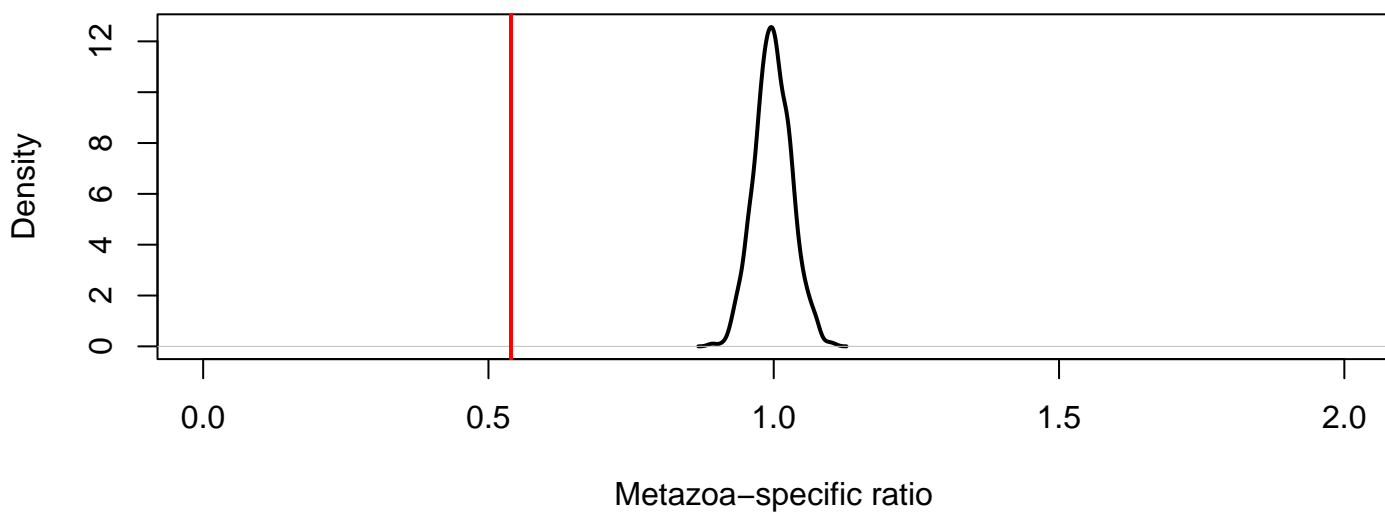

Supplement: Supplementary file 1 — Additional file 1: Zipped directory comprehending more detailed information on the species-specific transcriptomic analyses (transcriptomic statistics, read counts, fasta of upregulated transcripts, GO/IPR enrichment analyses, ratios bootstrap iterations) and extensive results of the comparative analyses (co-upregulated transcripts, co-enriched GO/IPR, comparisons of different cut-off iterations) [file 13227_2022_207_MOESM1_ESM.zip › SUPPLEMENTARY_MATERIAL/Ruditapes_philippinarum/Rph_1000sets_ratios.pdf]

### 1000 random sets of genes

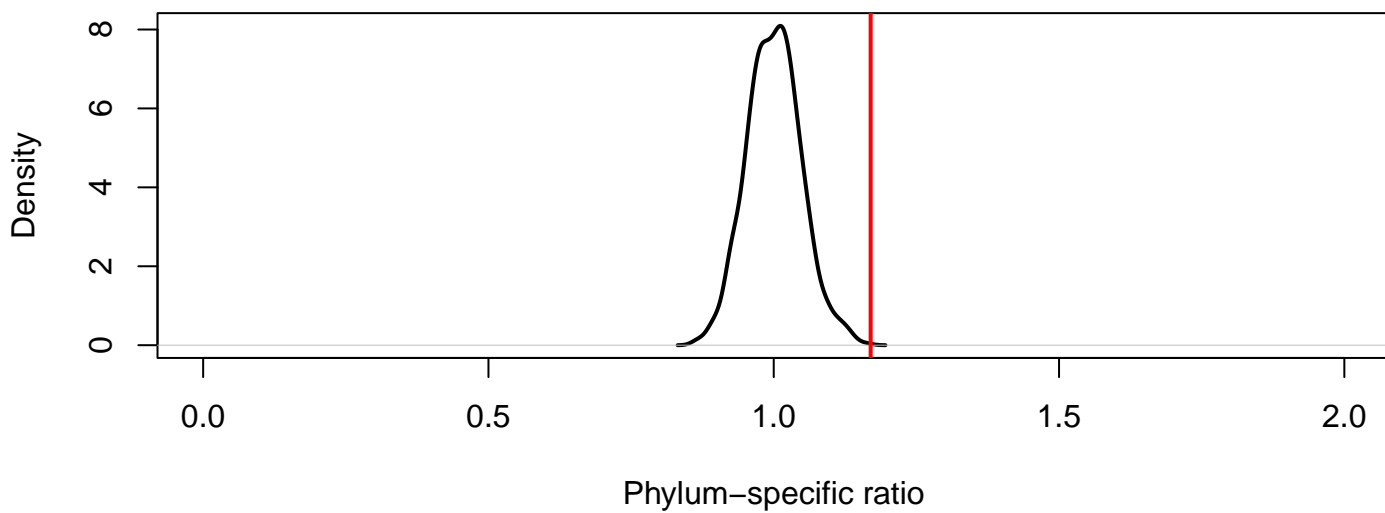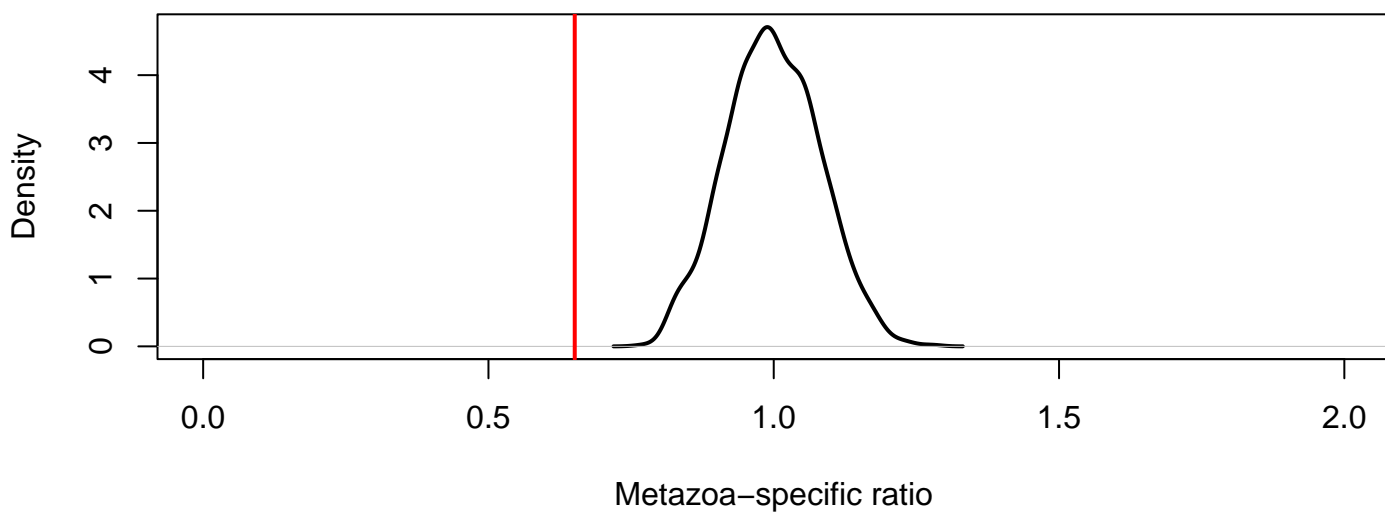

Supplement: Supplementary file 1 — Additional file 1: Zipped directory comprehending more detailed information on the species-specific transcriptomic analyses (transcriptomic statistics, read counts, fasta of upregulated transcripts, GO/IPR enrichment analyses, ratios bootstrap iterations) and extensive results of the comparative analyses (co-upregulated transcripts, co-enriched GO/IPR, comparisons of different cut-off iterations) [file 13227_2022_207_MOESM1_ESM.zip › SUPPLEMENTARY_MATERIAL/Caenorhabditis_elegans/Cel_1000sets_ratios.pdf]

# 1000 random sets of genes

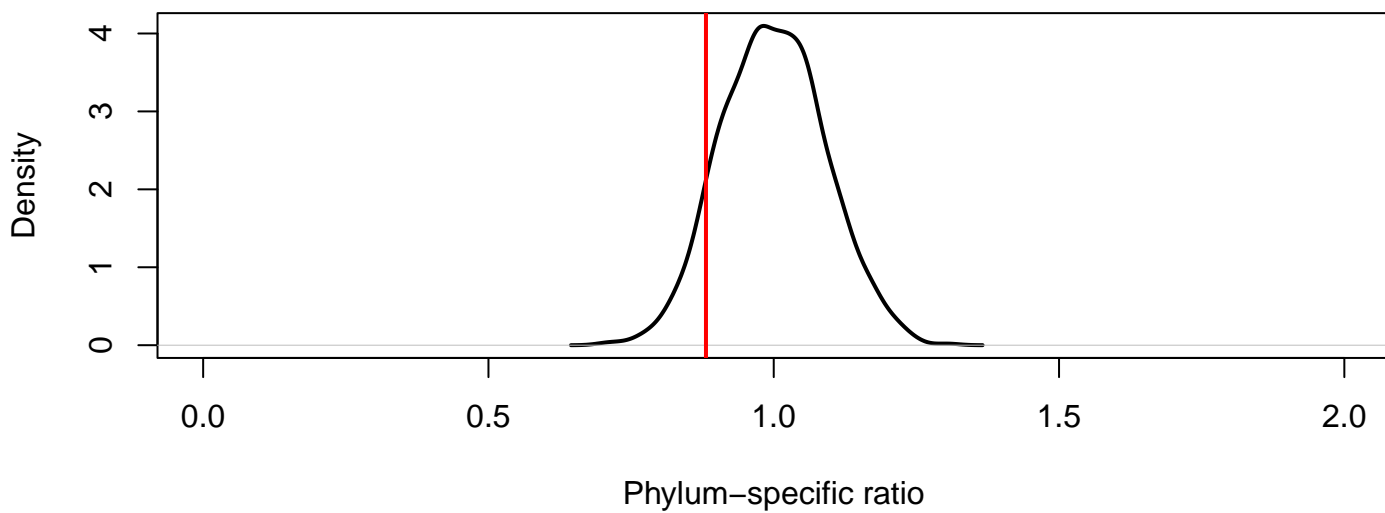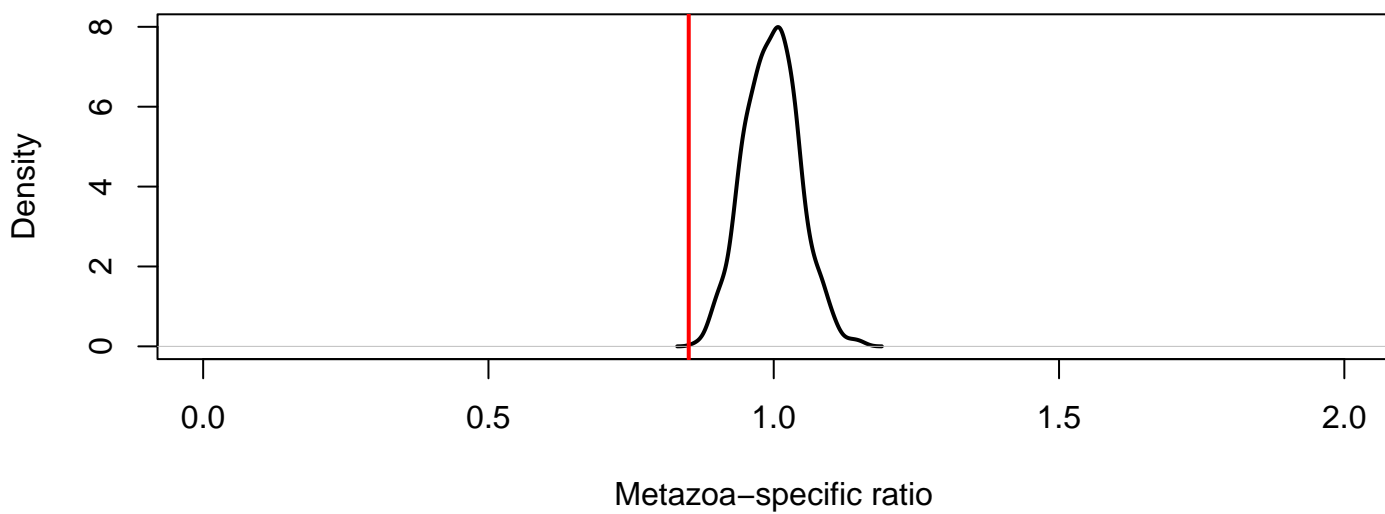

Supplement: Supplementary file 1 — Additional file 1: Zipped directory comprehending more detailed information on the species-specific transcriptomic analyses (transcriptomic statistics, read counts, fasta of upregulated transcripts, GO/IPR enrichment analyses, ratios bootstrap iterations) and extensive results of the comparative analyses (co-upregulated transcripts, co-enriched GO/IPR, comparisons of different cut-off iterations) [file 13227_2022_207_MOESM1_ESM.zip › SUPPLEMENTARY_MATERIAL/Haliotis_rufescens/Hru_1000sets_ratios.pdf]

# 1000 random sets of genes

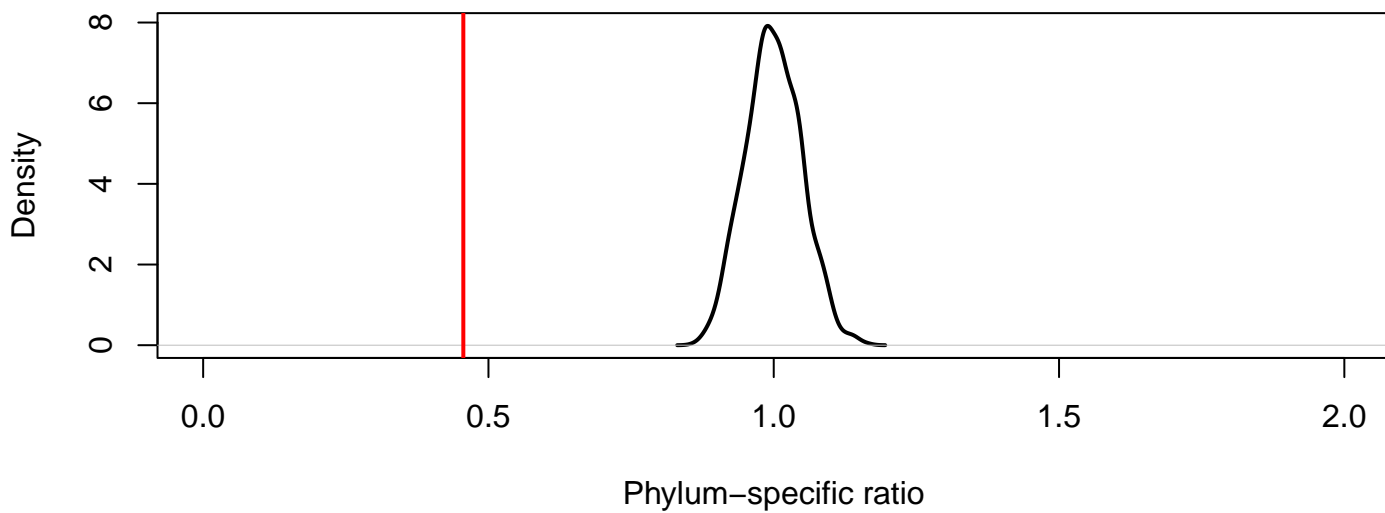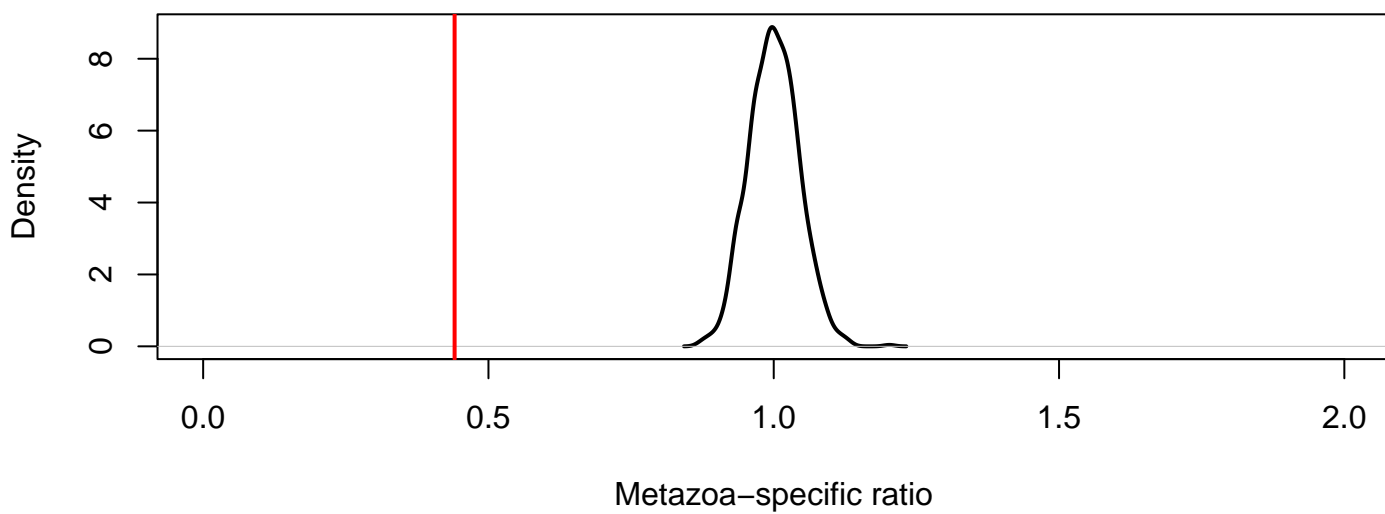

Supplement: Supplementary file 1 — Additional file 1: Zipped directory comprehending more detailed information on the species-specific transcriptomic analyses (transcriptomic statistics, read counts, fasta of upregulated transcripts, GO/IPR enrichment analyses, ratios bootstrap iterations) and extensive results of the comparative analyses (co-upregulated transcripts, co-enriched GO/IPR, comparisons of different cut-off iterations) [file 13227_2022_207_MOESM1_ESM.zip › SUPPLEMENTARY_MATERIAL/Ephydatia_fluviatilis/Efl_1000sets_ratios.pdf]

# 1000 random sets of genes

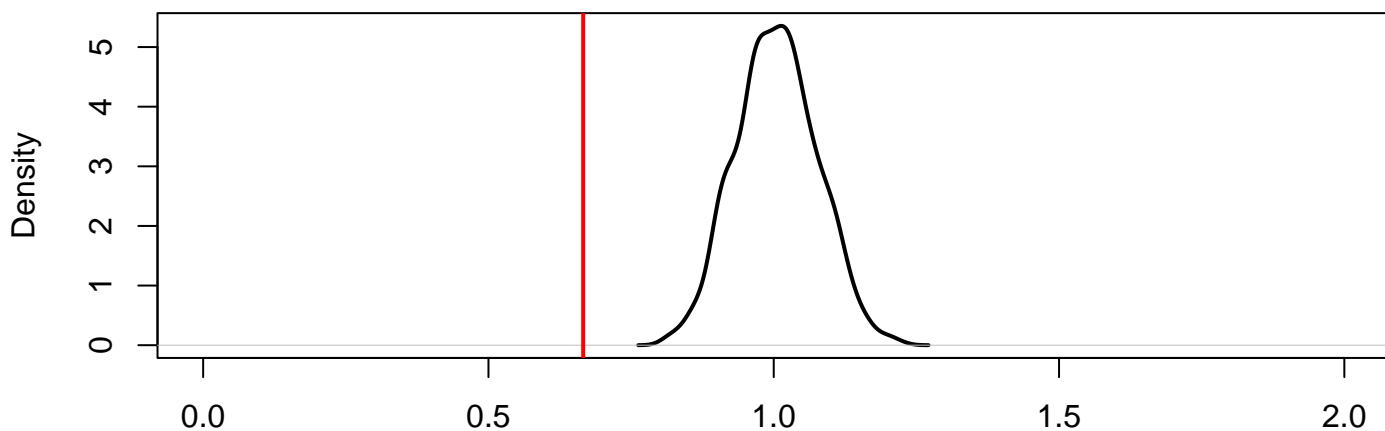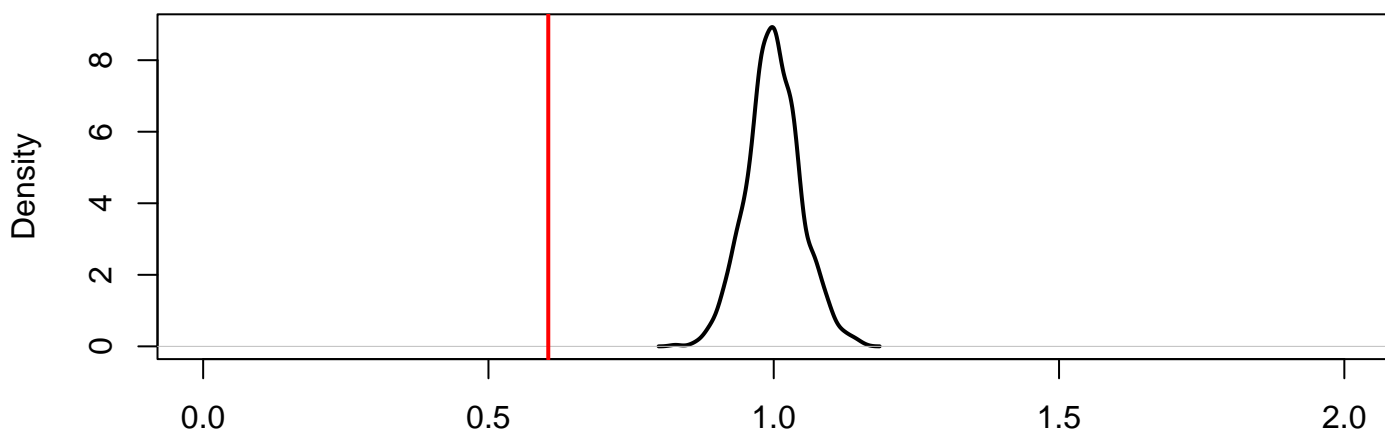

Metazoa-specific ratio

Supplement: Supplementary file 1 — Additional file 1: Zipped directory comprehending more detailed information on the species-specific transcriptomic analyses (transcriptomic statistics, read counts, fasta of upregulated transcripts, GO/IPR enrichment analyses, ratios bootstrap iterations) and extensive results of the comparative analyses (co-upregulated transcripts, co-enriched GO/IPR, comparisons of different cut-off iterations) [file 13227_2022_207_MOESM1_ESM.zip › SUPPLEMENTARY_MATERIAL/Nematostella_vectensis/Nve_1000sets_ratios.pdf]

# 1000 random sets of genes

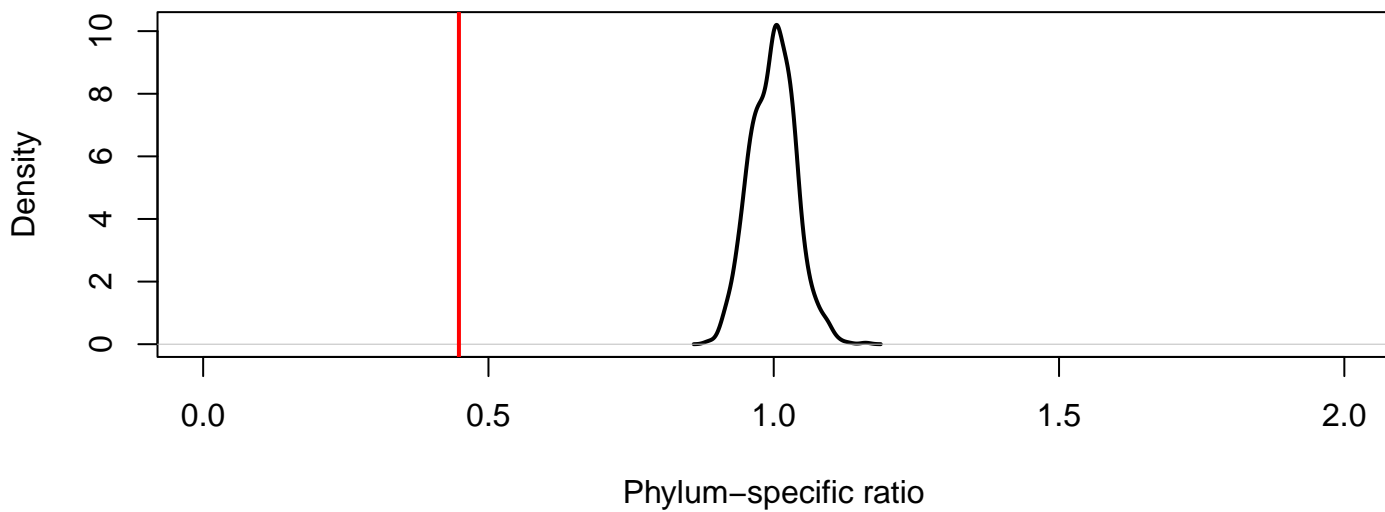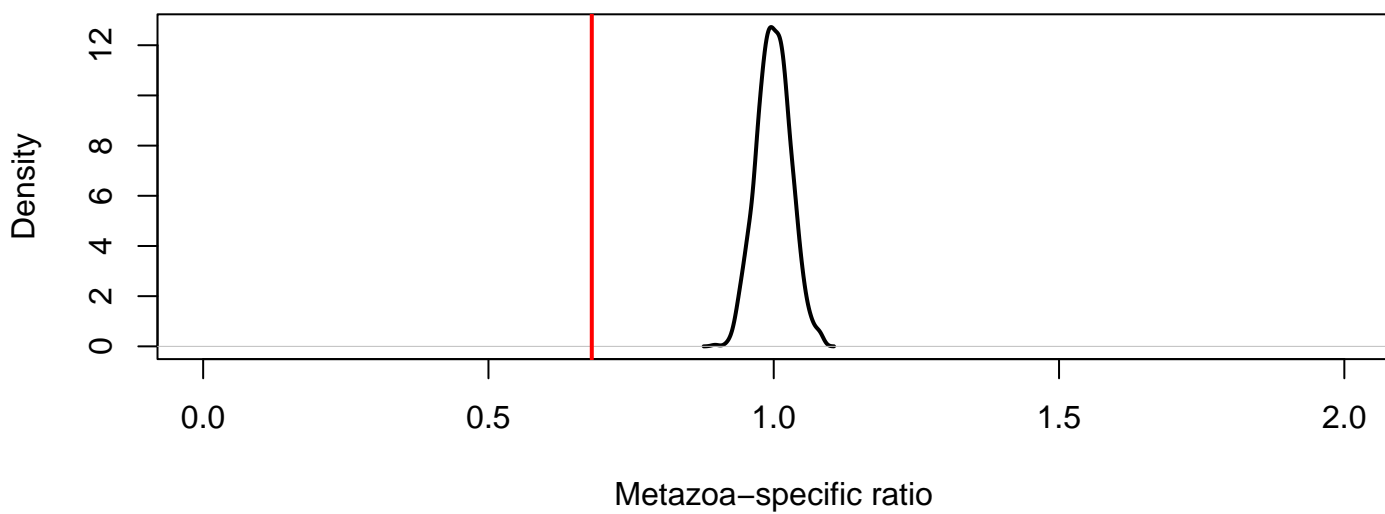

Supplement: Supplementary file 1 — Additional file 1: Zipped directory comprehending more detailed information on the species-specific transcriptomic analyses (transcriptomic statistics, read counts, fasta of upregulated transcripts, GO/IPR enrichment analyses, ratios bootstrap iterations) and extensive results of the comparative analyses (co-upregulated transcripts, co-enriched GO/IPR, comparisons of different cut-off iterations) [file 13227_2022_207_MOESM1_ESM.zip › SUPPLEMENTARY_MATERIAL/Xenopus_tropicalis/Xtr_1000sets_ratios.pdf]

# 1000 random sets of genes

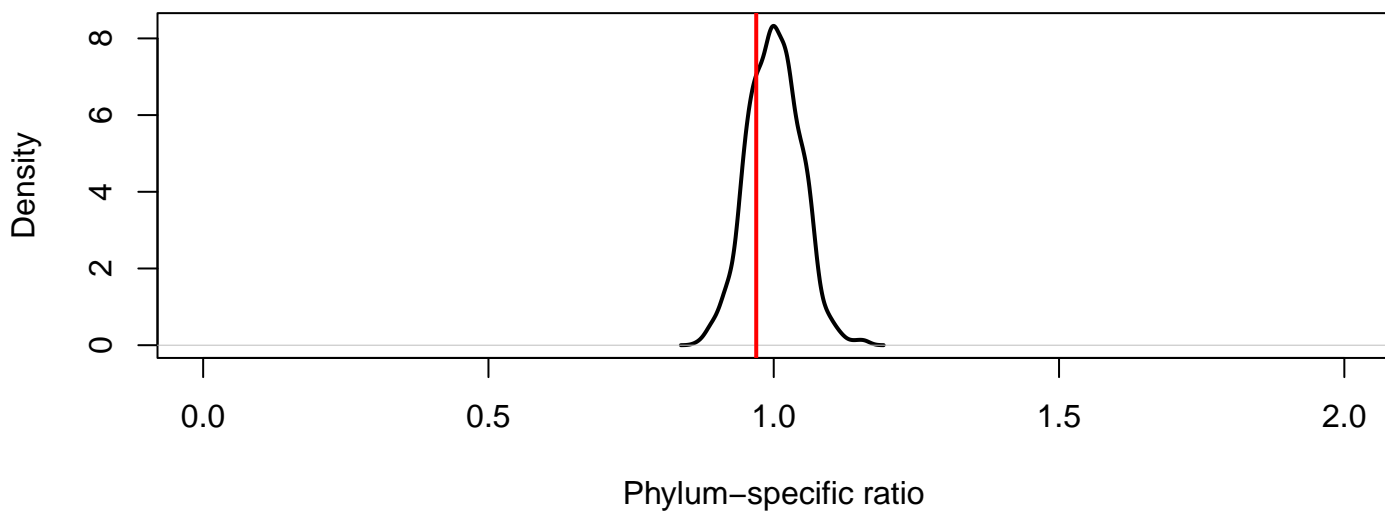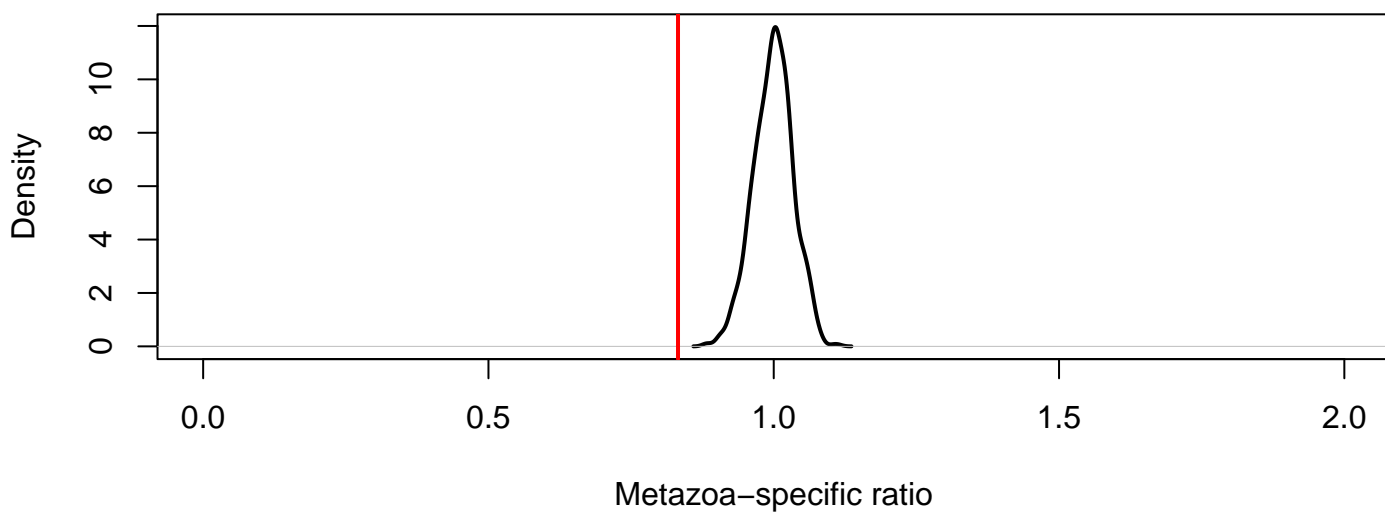

Supplement: Supplementary file 1 — Additional file 1: Zipped directory comprehending more detailed information on the species-specific transcriptomic analyses (transcriptomic statistics, read counts, fasta of upregulated transcripts, GO/IPR enrichment analyses, ratios bootstrap iterations) and extensive results of the comparative analyses (co-upregulated transcripts, co-enriched GO/IPR, comparisons of different cut-off iterations) [file 13227_2022_207_MOESM1_ESM.zip › SUPPLEMENTARY_MATERIAL/Danio_rerio/Dre_1000sets_ratios.pdf]
