# Supplementary material for: Germline-related molecular phenotype in Metazoa: conservation and innovation highlighted by comparative transcriptomics
Source: EvoDevo. 2023 Jan 30;14:2. doi: 10.1186/s13227-022-00207-3 (PMC9885605; doi:10.1186/s13227-022-00207-3)
Supplement: Supplementary file 2 — Additional file 2: Table S1. Species in the data set and accession codes of Genome assemblies. When the proteome was retrieved from an online source different from NCBI, the whole accession link is present. Phyla are in alphabetical order, with the four non-Metazoa phyla at the bottom of the table. Figure S1. GMP genes-associated IPR codes in germline-related samples. The table represents presence (light blue) or absence (light grey) in different species (columns) of IPR codes annotated for some representative GMP genes (whose transcript upregulation is depicted in Figure 2 of the main text). Considering associated domains and families rather than whole sequence homology improved the signal: for instance, virtually all domains belonging to vasa are biased in germline-related samples of all species, even if we could not observe vasa homologue upregulation for C. elegans and H. rufescens (Fig. 2 of the main text). Figure S2. Counts of co-upregulated OGs for all combinations of species. Each row represents the number of OGs that included upregulated germline-related transcripts in a precise number of species (from 2 to 9). For example, first row: 1803 OGs included germline-related upregulated sequences belonging to 2 species only (counting any possible 2-species combination). On the right the deviation from expected random distributions for the combinations of the corresponding number of species is reported: positive deviation from expectation is depicted in blue, negative deviation in red. For instance: the number of observed co-upregulated OGs in 4 species (any 4 species and only 4 species) was lower than expected; the number of observed co-upregulated OGs in 8 species (any 8 species and only 8 species) was higher than expected. Figure S3. Co-enriched GO terms (Biological Processes) in 6 or more species. The table represents presence (light blue) or absence (light grey) in different species (columns) of GO terms enriched in more than 2/3 of the data set. Figure S4. C [file 13227_2022_207_MOESM2_ESM.pdf]

Additional Information

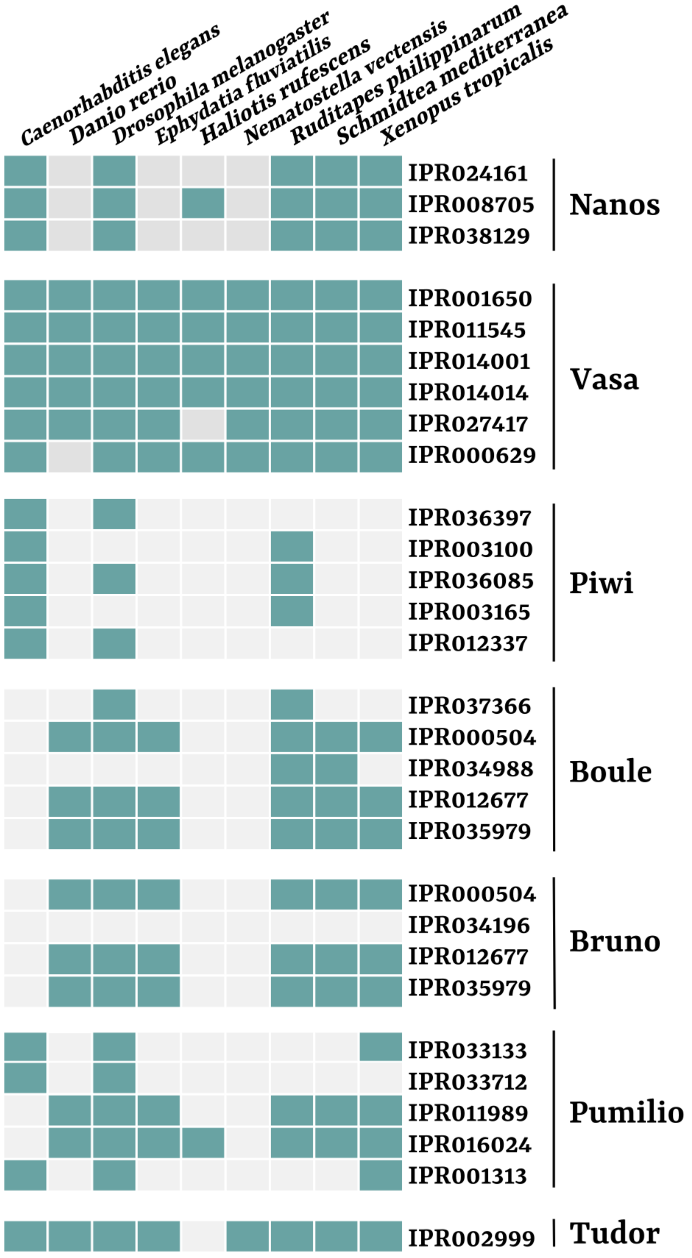

Additional file 1: Figure S1. GMP genes-associated IPR codes in germline-related samples. The table represents presence (light blue) or absence (light grey) in different species (columns) of IPR codes annotated for some representative GMP genes (whose transcript upregulation is depicted in Figure 2 of the main text). Considering associated domains and families rather than whole sequence homology improved the signal: for instance, virtually all domains belonging to *vasa* are biased in germline-related samples of all species, even if we could not observe *vasa* homologue upregulation for *C. elegans* and *H. rufescens* (Figure 2 of the main text).

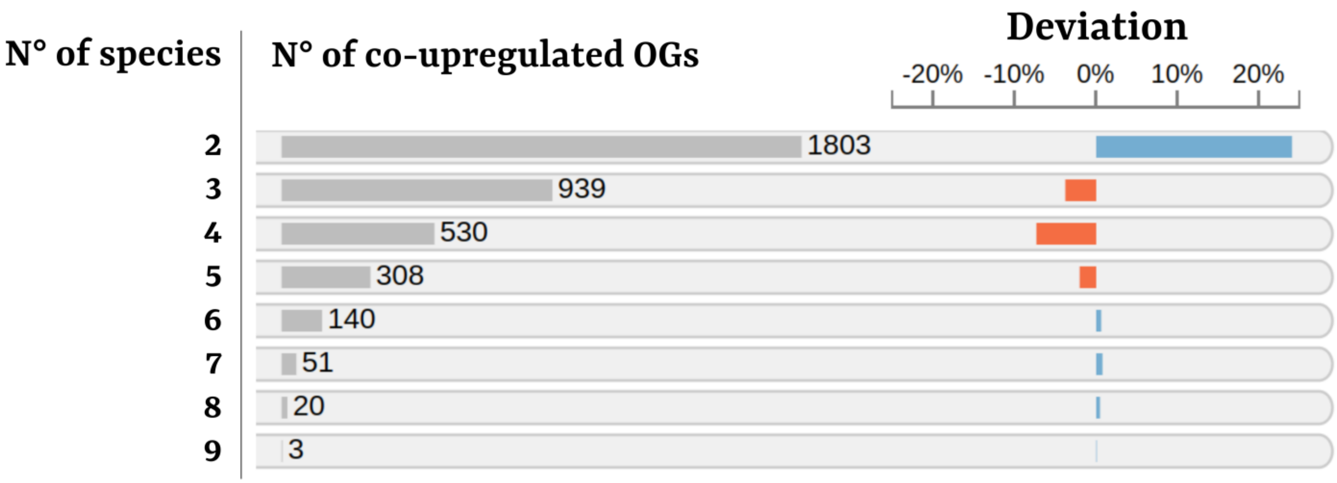

Additional file 1: Figure S2. Counts of co-upregulated OGs for all combinations of species. Each row represents the number of OGs that included upregulated germline-related transcripts in a precise number of species (from 2 to 9). For example, first row: 1803 OGs included germline-related upregulated sequences belonging to 2 species only (counting any possible 2-species combination). On the right the deviation from expected random distributions for the combinations of the corresponding number of species is reported: positive deviation from expectation is depicted in blue, negative deviation in red. For instance: the number of observed co-upregulated OGs in 4 species (any 4 species and only 4 species) was lower than expected; the number of observed co-upregulated OGs in 8 species (any 8 species and only 8 species) was higher than expected.

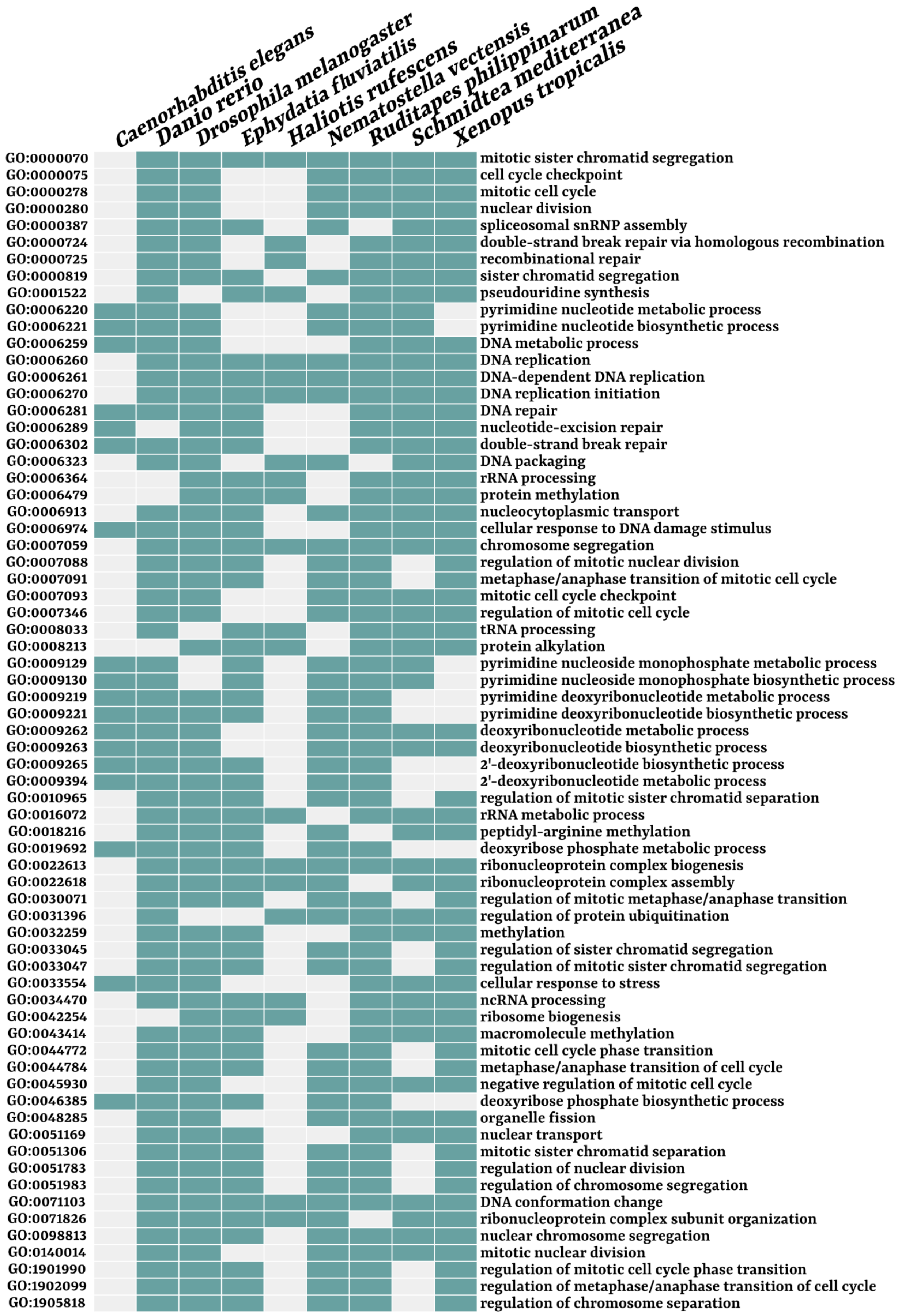

Caenorhabditis elegans
Danio rerio
Drosophila melanogaster
Ephydatia fluviatilis
Haliotis rufescens
Nematostella vectensis
Ruditapes philippinarum
Schmidtea mediterranea
Xenopus tropicalis
GO:0000070 mitotic sister chromatid segregation
GO:0000075 cell cycle checkpoint
GO:0000278 mitotic cell cycle
GO:0000280 nuclear division
GO:0000387 spliceosomal snRNP assembly
GO:0000724 double-strand break repair via homologous recombination
GO:0000725 recombinational repair
GO:0000819 sister chromatid segregation
GO:0001522 pseudouridine synthesis
GO:0006220 pyrimidine nucleotide metabolic process
GO:0006221 pyrimidine nucleotide biosynthetic process
GO:0006259 DNA metabolic process
GO:0006260 DNA replication
GO:0006261 DNA-dependent DNA replication
GO:0006270 DNA replication initiation
GO:0006281 DNA repair
GO:0006289 nucleotide-excision repair
GO:0006302 double-strand break repair
GO:0006323 DNA packaging
GO:0006364 rRNA processing
GO:0006479 protein methylation
GO:0006913 nucleocytoplasmic transport
GO:0006974 cellular response to DNA damage stimulus
GO:0007059 chromosome segregation
GO:0007088 regulation of mitotic nuclear division
GO:0007091 metaphase/anaphase transition of mitotic cell cycle
GO:0007093 mitotic cell cycle checkpoint
GO:0007346 regulation of mitotic cell cycle
GO:0008033 tRNA processing
GO:0008213 protein alkylation
GO:0009129 pyrimidine nucleoside monophosphate metabolic process
GO:0009130 pyrimidine nucleoside monophosphate biosynthetic process
GO:0009219 pyrimidine deoxyribonucleotide metabolic process
GO:0009221 pyrimidine deoxyribonucleotide biosynthetic process
GO:0009262 deoxyribonucleotide metabolic process
GO:0009263 deoxyribonucleotide biosynthetic process
GO:0009265 2'-deoxyribonucleotide biosynthetic process
GO:0009394 2'-deoxyribonucleotide metabolic process
GO:0010965 regulation of mitotic sister chromatid separation
GO:0016072 rRNA metabolic process
GO:0018216 peptidyl-arginine methylation
GO:0019692 deoxyribose phosphate metabolic process
GO:0022613 ribonucleoprotein complex biogenesis
GO:0022618 ribonucleoprotein complex assembly
GO:0030071 regulation of mitotic metaphase/anaphase transition
GO:0031396 regulation of protein ubiquitination
GO:0032259 methylation
GO:0033045 regulation of sister chromatid segregation
GO:0033047 regulation of mitotic sister chromatid segregation
GO:0033554 cellular response to stress
GO:0034470 ncRNA processing
GO:0042254 ribosome biogenesis
GO:0043414 macromolecule methylation
GO:0044772 mitotic cell cycle phase transition
GO:0044784 metaphase/anaphase transition of cell cycle
GO:0045930 negative regulation of mitotic cell cycle
GO:0046385 deoxyribose phosphate biosynthetic process
GO:0048285 organelle fission
GO:0051169 nuclear transport
GO:0051306 mitotic sister chromatid separation
GO:0051783 regulation of nuclear division
GO:0051983 regulation of chromosome segregation
GO:0071103 DNA conformation change
GO:0071826 ribonucleoprotein complex subunit organization
GO:0098813 nuclear chromosome segregation
GO:0140014 mitotic nuclear division
GO:1901990 regulation of mitotic cell cycle phase transition
GO:1902099 regulation of metaphase/anaphase transition of cell cycle
GO:1905818 regulation of chromosome separation

Additional file 1: Figure S3 (figure in previous page). Co-enriched GO terms (Biological Processes) in 6 or more species. The table represents presence (light blue) or absence (light grey) in different species (columns) of GO terms enriched in more than 2/3 of the data set.

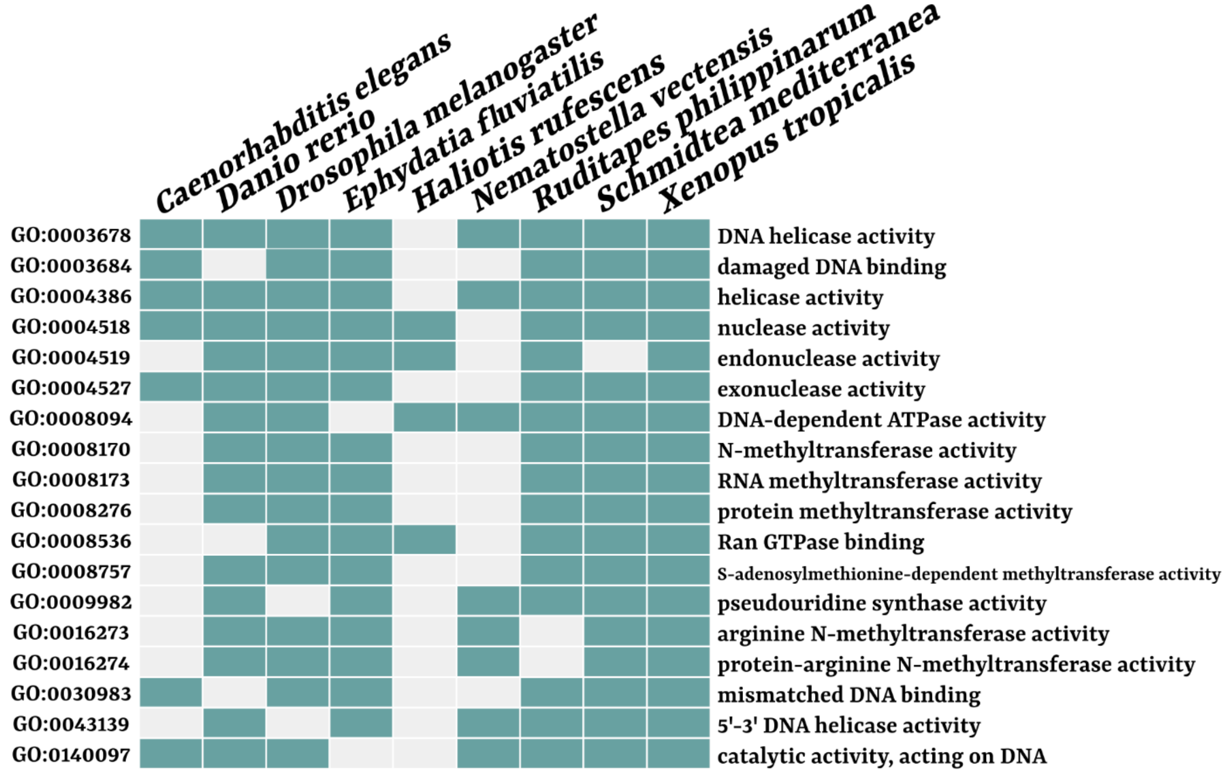

Additional file 1: Figure S4. Co-enriched GO terms (Molecular Functions) in 6 or more species. The table represents presence (light blue) or absence (light grey) in different species (columns) of GO terms enriched in more than 2/3 of the data set.

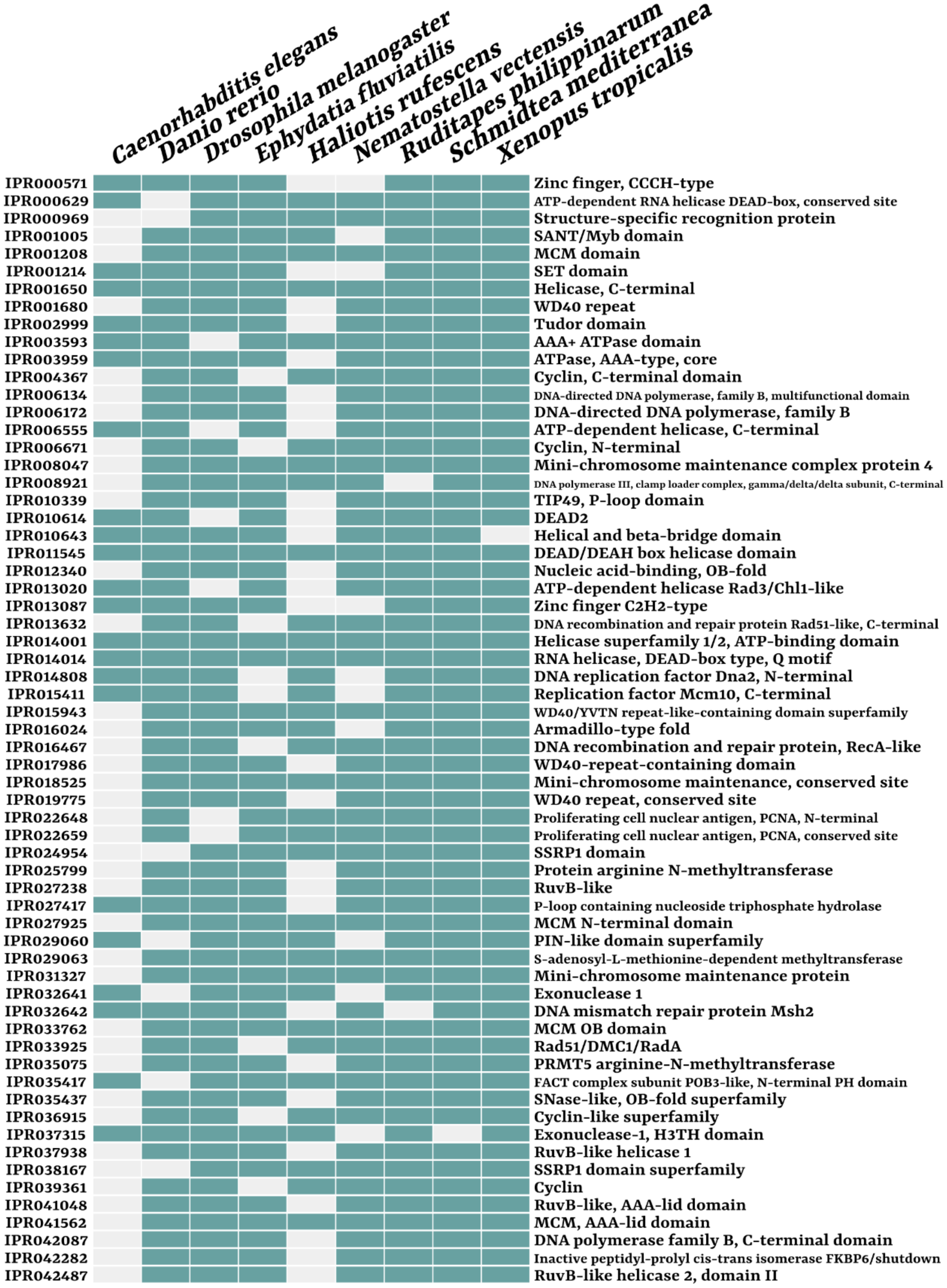

Additional file 1: Figure S5. Co-enriched IPR codes in 7 or more species. The table represents presence (light blue) or absence (light grey) in different species (columns). For DNA replication and repair activity information on the codes, refer to Table 2 of the main text

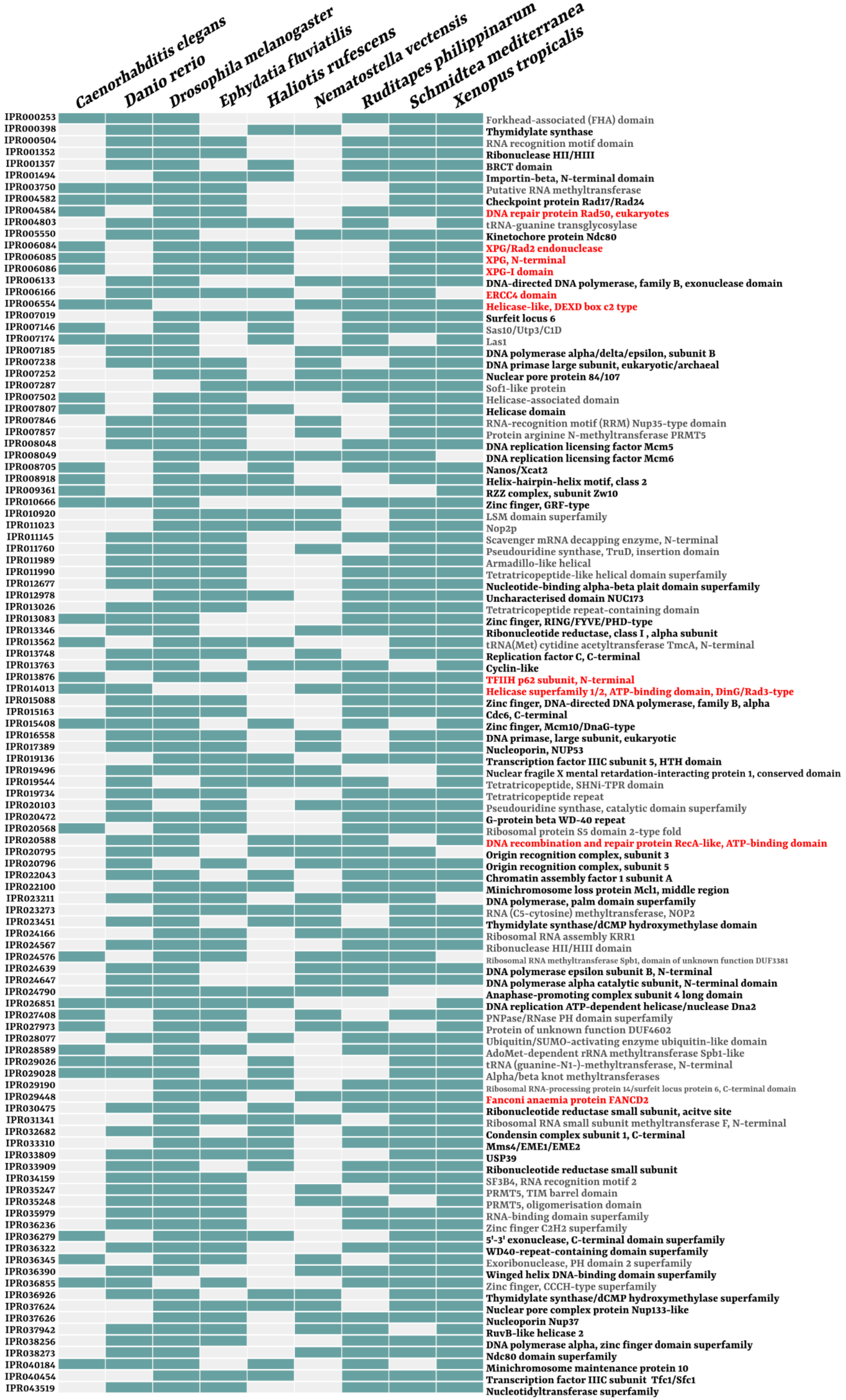
Caenorhabditis elegans
Danio rerio
Drosophila melanogaster
Ephydatia fluviatilis
Haliotis rufescens
Nematostella vectensis
Ruditapes philippinarum
Schmidtea mediterranea
Xenopus tropicalis
IPR000253 Forkhead-associated (FHA) domain
IPR000398 Thymidylate synthase
IPR000504 RNA recognition motif domain
IPR001352 Ribonuclease HII/HIII
IPR001357 BRCT domain
IPR001494 Importin-beta, N-terminal domain
IPR003750 Putative RNA methyltransferase
IPR004582 Checkpoint protein Rad17/Rad24
IPR004584 DNA repair protein Rad50, eukaryotes
IPR004803 tRNA-guanine transglycosylase
IPR005550 Kinetochore protein Ndc80
IPR006084 XPG/Rad2 endonuclease
IPR006085 XPG, N-terminal
IPR006086 XPG-I domain
IPR006133 DNA-directed DNA polymerase, family B, exonuclease domain
IPR006166 ERCC4 domain
IPR006554 Helicase-like, DEXD box c2 type
IPR007019 Surfeit locus 6
IPR007146 Sas10/Utp3/C1D
IPR007174 Las1
IPR007185 DNA polymerase alpha/delta/epsilon, subunit B
IPR007238 DNA primase large subunit, eukaryotic/archaeal
IPR007252 Nuclear pore protein 84/107
IPR007287 Sof1-like protein
IPR007502 Helicase-associated domain
IPR007807 Helicase domain
IPR007846 RNA-recognition motif (RRM) Nup35-type domain
IPR007857 Protein arginine N-methyltransferase PRMT5
IPR008048 DNA replication licensing factor Mcm5
IPR008049 DNA replication licensing factor Mcm6
IPR008705 Nanos/Xcat2
IPR008918 Helix-hairpin-helix motif, class 2
IPR009361 RZZ complex, subunit Zw10
IPR010666 Zinc finger, GRF-type
IPR010920 LSM domain superfamily
IPR011023 Nop2p
IPR011145 Scavenger mRNA decapping enzyme, N-terminal
IPR011760 Pseudouridine synthase, TruD, insertion domain
IPR011989 Armadillo-like helical
IPR011990 Tetratricopeptide-like helical domain superfamily
IPR012677 Nucleotide-binding alpha-beta plait domain superfamily
IPR012978 Uncharacterised domain NUC173
IPR013026 Tetratricopeptide repeat-containing domain
IPR013083 Zinc finger, RING/FYVE/PHD-type
IPR013346 Ribonucleotide reductase, class I , alpha subunit
IPR013562 tRNA(Met) cytidine acetyltransferase TmcA, N-terminal
IPR013748 Replication factor C, C-terminal
IPR013763 Cyclin-like
IPR013876 TFIIH p62 subunit, N-terminal
IPR014013 Helicase superfamily 1/2, ATP-binding domain, DinG/Rad3-type
IPR015088 Zinc finger, DNA-directed DNA polymerase, family B, alpha
IPR015163 Cdc6, C-terminal
IPR015408 Zinc finger, Mcm10/DnaG-type
IPR016558 DNA primase, large subunit, eukaryotic
IPR017389 Nucleoporin, NUP53
IPR019136 Transcription factor IIIC subunit 5, HTH domain
IPR019496 Nuclear fragile X mental retardation-interacting protein 1, conserved domain
IPR019544 Tetratricopeptide, SHNi-TPR domain
IPR019734 Tetratricopeptide repeat
IPR020103 Pseudouridine synthase, catalytic domain superfamily
IPR020472 G-protein beta WD-40 repeat
IPR020568 Ribosomal protein S5 domain 2-type fold
IPR020588 DNA recombination and repair protein RecA-like, ATP-binding domain
IPR020795 Origin recognition complex, subunit 3
IPR020796 Origin recognition complex, subunit 5
IPR022043 Chromatin assembly factor 1 subunit A
IPR022100 Minichromosome loss protein Mcl1, middle region
IPR023211 DNA polymerase, palm domain superfamily
IPR023273 RNA (C5-cytosine) methyltransferase, NOP2
IPR023451 Thymidylate synthase/dCMP hydroxymethylase domain
IPR024166 Ribosomal RNA assembly KRR1
IPR024567 Ribonuclease HII/HIII domain
IPR024576 Ribosomal RNA methyltransferase Spb1, domain of unknown function DUF3381
IPR024639 DNA polymerase epsilon subunit B, N-terminal
IPR024647 DNA polymerase alpha catalytic subunit, N-terminal domain
IPR024790 Anaphase-promoting complex subunit 4 long domain
IPR026851 DNA replication ATP-dependent helicase/nuclease Dna2
IPR027408 PNPase/RNase PH domain superfamily
IPR027973 Protein of unknown function DUF4602
IPR028077 Ubiquitin/SUMO-activating enzyme ubiquitin-like domain
IPR028589 AdoMet-dependent rRNA methyltransferase Spb1-like
IPR029026 tRNA (guanine-N1-)-methyltransferase, N-terminal
IPR029028 Alpha/beta knot methyltransferases
IPR029190 Ribosomal RNA-processing protein 14/surfeit locus protein 6, C-terminal domain
IPR029448 Fanconi anaemia protein FANCD2
IPR030475 Ribonucleotide reductase small subunit, acitve site
IPR031341 Ribosomal RNA small subunit methyltransferase F, N-terminal
IPR032682 Condensin complex subunit 1, C-terminal
IPR033310 Mms4/EME1/EME2
IPR033809 USP39
IPR033909 Ribonucleotide reductase small subunit
IPR034159 SF3B4, RNA recognition motif 2
IPR035247 PRMT5, TIM barrel domain
IPR035248 PRMT5, oligomerisation domain
IPR035979 RNA-binding domain superfamily
IPR036236 Zinc finger C2H2 superfamily
IPR036279 5'-3' exonuclease, C-terminal domain superfamily
IPR036322 WD40-repeat-containing domain superfamily
IPR036345 Exoribonuclease, PH domain 2 superfamily
IPR036390 Winged helix DNA-binding domain superfamily
IPR036855 Zinc finger, CCCH-type superfamily
IPR036926 Thymidylate synthase/dCMP hydroxymethylase superfamily
IPR037624 Nuclear pore complex protein Nup133-like
IPR037626 Nucleoporin Nup37
IPR037942 RuvB-like helicase 2
IPR038256 DNA polymerase alpha, zinc finger domain superfamily
IPR038273 Ndc80 domain superfamily
IPR040184 Minichromosome maintenance protein 10
IPR040454 Transcription factor IIIC subunit Tfc1/Sfc1
IPR043519 Nucleotidyltransferase superfamily

Additional file 1: Figure S6 (figure in previous page). Co-enriched IPR codes in 6 species. The table represents presence (light blue) or absence (light grey) in different species (columns). Code annotation that refer to strictly DNA-related or proliferative activities are highlighted in bold. Codes associated to DNA repair are highlighted in red.

Additional file 1: Table S1. Species in the data set and accession codes of Genome assemblies. When the proteome was retrieved from an online source different from NCBI, the whole accession link is present. Phyla are in alphabetical order, with the four non-Metazoa phyla at the bottom of the table.

Phylum	Species	Genome code / database
**ANNELIDA**	*Capitella teleta*	GCA_000328365.1
	Dimorphilus gyrociliatus	GCA_904063045.1
	Helobdella robusta	GCF_000326865.1
	Owenia fusiformis	GCA_903813345.1
**ARTHROPODA**	*Aphis gossypii*	GCF_004010815.1
	Apis mellifera	GCF_003254395.2
	Centruroides sculpturatus	GCF_000671375.1
	Cloeon dipterum	GCA_902829235.1
	Cryptotermes secundus	GCF_002891405.2
	Ctenocephalides felis	GCF_003426905.1
	Daphnia magna	GCF_003990815.1
	Dermatophagoides pteronyssinus	GCF_001901225.1
	Drosophila melanogaster	GCF_000001215.4
	Eurytemora affinis	GCF_000591075.1
	Folsomia candida	GCF_002217175.1
	Hyalella azteca	GCF_000764305.1
	Ixodes scapularis	GCF_002892825.2
	Lepeophtheirus salmonis	GCF_016086655.3
	Limulus polyphemus	GCF_000517525.1
	Nymphon striatum	GCA_016618385.1
	Parasteatoda tepidariorum	GCF_000365465.2
	Penaeus vannamei	GCF_003789085.1
	Sitophilus oryzae	GCF_002938485.1
	Tetranychus urticae	GCF_000239435.1
	Varroa destructor	GCF_002443255.1
**BRACHIOPODA**	*Lingula anatina*	GCF_001039355.2

**BRIOZOA**	*Bugula neritina*	GCA_010799875.2
**CHORDATA**	*Acipenser ruthenus*	GCF_010645085.1
	Amblyraja radiata	GCF_010909765.1
	Branchiostoma floridae	GCF_000003815.2
	Ciona intestinalis	GCF_000224145.3
	Danio rerio	GCF_000002035.6
	Gallus gallus	GCF_000002315.6
	Gekko japonicus	GCF_001447785.1
	Homo sapiens	GCF_000001405.39
	Oikopleura dioica	GCA_000209555.1
	Petromyzon marinus	GCF_010993605.1
	Styela clava	GCF_013122585.1
	Xenopus tropicalis	GCF_000004195.4
**CNIDARIA**	*Acropora digitifera*	GCF_000222465.1
	Actinia tenebrosa	GCF_009602425.1
	Aurelia aurita	https://marinegenomics.oist.jp/aurelia_aurita/
	Clytia hemisphaerica	http://ftp.ensemblgenomes.org/pub/metazoa/ release-52/fasta/clytia_hemisphaerica_gca902728285
	Dendronephthya gigantea	GCF_004324835.1
	Exaiptasia diaphana	GCF_001417965.1
	Henneguya salminicola	GCA_009887335.1
	Hydra vulgaris	GCF_000004095.1
	Morbakka virulenta	https://marinegenomics.oist.jp/morbakka_virulenta/
	Myxobolus squamalis	GCA_010108815.1
	Nematostella vectensis	GCF_000209225.1
	Orbicella faveolata	GCF_002042975.1
	Pocillopora damicornis	GCF_003704095.1
	Stylophora pistillata	GCF_002571385.1
	Thelohanellus kitauei	GCA_000827895.1
**CTENOPHORA**	*Hormiphora californiensis*	https://github.com/conchoecia/hormiphora
	Mnemiopsis leidyi	https://research.nhgri.nih.gov/mnemiopsis
**ECHINODERMATA**	*Anneissia japonica*	GCF_011630105.1
	Apostichopus japonicus	GCA_002754855.1
	Asterias rubens	GCF_902459465.1
	Strongylocentrotus purpuratus	GCF_000002235.5
**HEMICHORDATA**	*Ptychodera flava*	https://marinegenomics.oist.jp/acornworm/
	Saccoglossus kowalevskii	GCF_000003605.2
**MOLLUSCA**	*Aplysia californica*	GCF_000002075.1
	Biomphalaria glabrata	GCF_000457365.1
	Crassostrea gigas	GCF_902806645.1
	Lottia gigantea	GCF_000327385.1
	Mizuhopecten yessoensis	GCF_002113885.1
	Octopus bimaculoides	GCF_001194135.1
	Pomacea canaliculata	GCF_003073045.1
**NEMATODA**	*Aphelenchus avenae*	GCA_020875895.1
	Brugia malayi	GCF_000002995.3

	Bursaphelenchus okinawaensis	GCA_904066225.2
	Caenorhabditis elegans	GCF_000002985.6
	Loa loa	GCF_000183805.1
	Necator americanus	GCF_000507365.1
	Plectus sambesii	GCA_002796945.1
	Strongyloides ratti	GCF_001040885.1
	Trichinella spiralis	GCF_000181795.1
**NEMERTEA**	*Notospermus geniculatus*	https://marinegenomics.oist.jp/nge_v2/
**ORTHONECTIDA**	*Intoshia linei*	GCA_001642005.1
**PHORONIDA**	*Phoronis australis*	https://marinegenomics.oist.jp/pau_v2/
**PLACOZOA**	*Trichoplax adhaerens*	GCF_000150275.1
	Trichoplax spH2	GCA_003344405.1
**PLATYHELMINTHES**	*Dibothriocephalus latus*	GCA_900617775.1
	Echinococcus granulosus	GCF_000524195.1
	Fasciola hepatica	GCA_002763495.2
	Macrostomum lignano	GCA_002269645.1
	Opisthorchis viverrini	GCF_000715545.1
	Protopolystoma xenopodis	GCA_900617795.1
	Schistosoma mansoni	GCF_000237925.1
	Schmidtea mediterranea	(SMSG.1)
**PORIFERA**	*Amphimedon queenslandica*	GCF_000090795.1
	Ephydatia muelleri	https://spaces.facsci.ualberta.ca/ephybase/
**PRIAPULIDA**	*Priapulus caudatus*	GCF_000485595.1
**MESOZOA**	*Dicyema japonicum*	GCA_011109175.1
**ROTIFERA**	*Adineta ricciae*	GCA_905250095.1
	Brachionus calyciflorus	GCA_905250105.1
	Didymodactylos carnosus	GCA_905250885.1
	Rotaria socialis	GCA_905332285.1
**TARDIGRADA**	*Hypsibius dujardini*	GCA_002082055.1
	Ramazzottius varieornatus	GCA_001949185.1
**XENACOELOMORPHA**	*Praesagittifera naikaiensis*	http://gigadb.org/dataset/100564
	Xenoturbella bocki	https://figshare.com/articles/dataset/ Genome_of_Xenoturbella_bocki/
**CHOANOFLAGELLATA**	*Monosiga brevicollis*	http://ftp.ensemblgenomes.org/pub/protists/ release-52/fasta/protists_choanoflagellida1_collection/ monosiga_brevicollis_mx1_gca_000002865/
	Salpingoeca rosetta	GCA_000188695.1
**FILASTEREA**	*Capsaspora owczarzaki*	GCF_000151315.2
**ICHTHYOSPOREA**	*Chromosphaera perkinsii*	https://figshare.com/articles/dataset/ Genome_-_Chromosphaera_perkinsii/
	Ichthyophonus hoferi	https://figshare.com/articles/dataset/ Genome_-_Ichthyophonus_hoferi/
	Pirum gemmata	https://figshare.com/articles/dataset/ Genome_-_Pirum_gemmata/
	Sphaeroforma arctica	GCF_001186125.1
**PLURIFORMEA**	*Corallochytrium limacisporum*	https://figshare.com/articles/dataset/ Genome_-_Corallochytrium_limacisporum/
